# Supplementary material for: Modeling of Protein–Protein Interactions in Cytokinin Signal Transduction
Source: Int J Mol Sci. 2019 Apr 28;20(9):2096. doi: 10.3390/ijms20092096 (PMC6539988; doi:10.3390/ijms20092096)
Supplement: Supplementary file 1 [file ijms-20-02096-s001.zip › Supplementary_dataset_2.docx]

**Supplementary dataset 2**

to the paper " Modeling of protein-protein interactions in cytokinin signal transduction" by Dmitry V. Arkhipov, Sergey N. Lomin, Yulia A. Myakushina, Ekaterina M. Savelieva, Dmitry I. Osolodkin, and Georgy A. Romanov. Lists of possible hydrogen bonds and salt bridges for all modeled complexes discussed in this article according to PISA results.

**HKsm-HKsm complexes**

| **AHK2sm-AHK2sm** | | | |  | **AHK2sm-AHK3sm** | | | |
| --- | --- | --- | --- | --- | --- | --- | --- | --- |
|  |  |  |  |  |  |  |  |  |
| Hydrogen bonds | | | |  | Hydrogen bonds | | | |
|  | Monomer 1 | Length | Monomer 2 |  |  | Monomer 1 | Length | Monomer 2 |
| 1 | A:HIS 286 [ HE2] | 2,11 | B:ASN 285 [ OD1] |  | 1 | A:TYR 314 [ HH ] | 2,01 | B:ALA 150 [ O  ] |
| 2 | A:THR 296 [ HG1] | 1,67 | B:GLU 313 [ OE2] |  | 2 | A:ARG 317 [HH21] | 2,46 | B:ILE 153 [ O  ] |
| 3 | A:LYS 301 [ HZ2] | 1,80 | B:ALA 305 [ O  ] |  | 3 | A:LYS 301 [ HZ2] | 1,82 | B:THR 171 [ OG1] |
| 4 | A:TYR 314 [ HH ] | 1,98 | B:ALA 289 [ O  ] |  | 4 | A:THR 296 [ HG1] | 1,63 | B:GLU 174 [ OE2] |
| 5 | A:ARG 317 [HH22] | 1,91 | B:SER 295 [ OG ] |  | 5 | A:SER 295 [ OG ] | 1,96 | B:ARG 178 [HH12] |
| 6 | A:SER 295 [ OG ] | 1,86 | B:ARG 317 [HH12] |  | 6 | A:SER 295 [ OG ] | 2,32 | B:ARG 178 [HH22] |
| 7 | A:GLY 300 [ O  ] | 2,10 | B:ARG 309 [HH22] |  | 7 | A:ALA 305 [ O  ] | 1,93 | B:LYS 162 [ HZ3] |
| 8 | A:GLY 300 [ O  ] | 1,82 | B:ARG 309 [HH12] |  | 8 | A:THR 310 [ OG1] | 1,85 | B:LYS 162 [ HZ1] |
| 9 | A:ALA 305 [ O  ] | 2,45 | B:LYS 301 [ HZ1] |  | 9 | A:GLU 313 [ OE2] | 1,58 | B:THR 157 [ HG1] |
| 10 | A:GLU 313 [ OE2] | 1,55 | B:THR 296 [ HG1] |  | 10 | A:GLU 321 [ OE1] | 1,74 | B:ASN 146 [HD21] |
|  |  |  |  |  |  |  |  |  |
| Salt bridges | | | |  | Salt bridges | | | |
|  | Monomer 1 | Length | Monomer 2 |  |  | Monomer 1 | Length | Monomer 2 |
| 1 | A:LYS 301 [ NZ ] | 2,67 | B:ASP 307 [ OD2] |  | 1 | A:LYS 301 [ NZ ] | 3,60 | B:ASP 168 [ OD1] |
| 2 | A:ASP 307 [ OD1] | 2,75 | B:LYS 301 [ NZ ] |  | 2 | A:LYS 301 [ NZ ] | 2,67 | B:ASP 168 [ OD2] |
|  |  |  |  |  | 3 | A:ASP 307 [ OD2] | 2,72 | B:LYS 162 [ NZ ] |
|  |  |  |  |  |  |  |  |  |
|  |  |  |  |  |  |  |  |  |
| **AHK2sm-AHK4sm** | | | |  | **AHK3sm-AHK3sm** | | | |
|  |  |  |  |  |  |  |  |  |
| Hydrogen bonds | | | |  | Hydrogen bonds | | | |
|  | Monomer 1 | Length | Monomer 2 |  |  | Monomer 1 | Length | Monomer 2 |
| 1 | A:HIS 286 [ HE2] | 2,34 | B:ASN 181 [ OD1] |  | 1 | A:ARG 178 [HH12] | 2,02 | B:SER 156 [ OG ] |
| 2 | A:ARG 317 [HH12] | 1,95 | B:SER 191 [ OG ] |  | 2 | A:ARG 178 [HH22] | 2,01 | B:SER 156 [ O  ] |
| 3 | A:LYS 301 [ HZ2] | 2,07 | B:THR 206 [ OG1] |  | 3 | A:ASN 146 [HD21] | 1,74 | B:GLU 182 [ OE1] |
| 4 | A:THR 296 [ HG1] | 1,57 | B:GLU 209 [ OE1] |  | 4 | A:ALA 150 [ O  ] | 2,23 | B:TYR 175 [ HH ] |
| 5 | A:ALA 289 [ O  ] | 1,98 | B:TYR 210 [ HH ] |  | 5 | A:SER 156 [ OG ] | 1,87 | B:ARG 178 [HH22] |
| 6 | A:SER 295 [ OG ] | 1,90 | B:ARG 213 [HH11] |  | 6 | A:SER 156 [ O  ] | 1,92 | B:ARG 178 [HH12] |
| 7 | A:ALA 305 [ O  ] | 2,36 | B:LYS 197 [ HZ1] |  | 7 | A:ALA 166 [ O  ] | 2,25 | B:LYS 162 [ HZ1] |
| 8 | A:ALA 305 [ O  ] | 2,49 | B:LYS 197 [ HZ3] |  | 8 | A:THR 171 [ OG1] | 1,89 | B:LYS 162 [ HZ2] |
| 9 | A:GLU 313 [ OE2] | 1,67 | B:THR 192 [ HG1] |  | 9 | A:GLU 174 [ O  ] | 2,47 | B:THR 157 [ HG1] |
|  |  |  |  |  | 10 | A:GLU 182 [ OE1] | 1,88 | B:ASN 146 [HD21] |
| Salt bridges | | | |  | 11 | A:GLU 182 [ OE2] | 2,47 | B:ALA 150 [ H  ] |
|  | Monomer 1 | Length | Monomer 2 |  |  |  |  |  |
| 1 | A:LYS 301 [ NZ ] | 2,68 | B:ASP 203 [ OD2] |  | Salt bridges | | | |
| 2 | A:ASP 307 [ OD1] | 2,88 | B:LYS 197 [ NZ ] |  |  | Monomer 1 | Length | Monomer 2 |
|  |  |  |  |  | 1 | A:LYS 162 [ NZ ] | 2,67 | B:ASP 168 [ OD2] |
|  |  |  |  |  | 2 | A:ARG 303 [ NH1] | 3,49 | B:GLU 174 [ OE1] |
|  |  |  |  |  | 3 | A:ARG 303 [ NH1] | 3,18 | B:GLU 174 [ OE2] |
|  |  |  |  |  | 4 | A:ASP 168 [ OD1] | 3,92 | B:LYS 162 [ NZ ] |
|  |  |  |  |  | 5 | A:ASP 168 [ OD2] | 2,72 | B:LYS 162 [ NZ ] |

| **AHK3sm-AHK4sm** | | | |  | **AHK4sm-AHK4sm** | | | |
| --- | --- | --- | --- | --- | --- | --- | --- | --- |
|  |  |  |  |  |  |  |  |  |
| Hydrogen bonds | | | |  | Hydrogen bonds | | | |
|  | Monomer 1 | Length | Monomer 2 |  |  | Monomer 1 | Length | Monomer 2 |
| 1 | A:ARG 178 [HH21] | 1,81 | B:SER 191 [ OG ] |  | 1 | A:TYR 210 [ HH ] | 2,00 | B:ALA 185 [ O  ] |
| 2 | A:THR 157 [ HG1] | 1,61 | B:GLU 209 [ OE2] |  | 2 | A:ARG 213 [HH21] | 1,86 | B:SER 191 [ OG ] |
| 3 | A:ALA 150 [ O  ] | 1,94 | B:TYR 210 [ HH ] |  | 3 | A:LYS 197 [ HZ3] | 2,20 | B:ALA 201 [ O  ] |
| 4 | A:SER 156 [ OG ] | 1,84 | B:ARG 213 [HH22] |  | 4 | A:LYS 197 [ HZ2] | 2,37 | B:THR 206 [ OG1] |
| 5 | A:SER 156 [ O  ] | 2,06 | B:ARG 213 [HH21] |  | 5 | A:ASN 181 [HD21] | 1,74 | B:GLU 217 [ OE1] |
| 6 | A:ALA 166 [ O  ] | 2,32 | B:LYS 197 [ HZ1] |  | 6 | A:ALA 185 [ O  ] | 1,92 | B:TYR 210 [ HH ] |
| 7 | A:THR 171 [ OG1] | 1,87 | B:LYS 197 [ HZ1] |  | 7 | A:SER 191 [ OG ] | 1,88 | B:ARG 213 [HH11] |
| 8 | A:GLU 174 [ OE2] | 1,64 | B:THR 192 [ HG1] |  | 8 | A:GLU 209 [ OE1] | 1,57 | B:THR 192 [ HG1] |
|  |  |  |  |  |  |  |  |  |
| Salt bridges | | | |  | Salt bridges | | | |
|  | Monomer 1 | Length | Monomer 2 |  |  | Monomer 1 | Length | Monomer 2 |
| 1 | A:LYS 162 [ NZ ] | 3,78 | B:ASP 203 [ OD1] |  | 1 | A:LYS 197 [ NZ ] | 3,52 | B:ASP 203 [ OD1] |
| 2 | A:LYS 162 [ NZ ] | 2,64 | B:ASP 203 [ OD2] |  | 2 | A:LYS 197 [ NZ ] | 2,68 | B:ASP 203 [ OD2] |
|  |  |  |  |  | 3 | A:ASP 203 [ OD1] | 2,84 | B:LYS 197 [ NZ ] |
|  |  |  |  |  | 4 | A:ASP 203 [ OD2] | 2,68 | B:LYS 197 [ NZ ] |
|  |  |  |  |  | 5 | A:GLU 205 [ OE2] | 2,70 | B:LYS 197 [ NZ ] |
|  |  |  |  |  |  |  |  |  |
| **StHK2sm-StHK2sm** | | | |  | **StHK3sm-StHK3sm** | | | |
|  |  |  |  |  |  |  |  |  |
| Hydrogen bonds | | | |  | Hydrogen bonds | | | |
|  | Monomer 1 | Length | Monomer 2 |  |  | Monomer 1 | Length | Monomer 2 |
| 1 | A:TYR 392 [ HH ] | 2,01 | B:ALA 367 [ O  ] |  | 1 | A:ARG 164 [HH12] | 1,98 | B:SER 142 [ OG ] |
| 2 | A:ARG 395 [HH22] | 1,85 | B:SER 373 [ OG ] |  | 2 | A:ARG 164 [HH21] | 1,91 | B:SER 142 [ OG ] |
| 3 | A:ARG 395 [HH12] | 2,32 | B:SER 373 [ OG ] |  | 3 | A:ARG 148 [ HE ] | 2,06 | B:THR 157 [ OG1] |
| 4 | A:THR 374 [ HG1] | 1,63 | B:GLU 391 [ OE1] |  | 4 | A:ALA 136 [ O  ] | 2,29 | B:TYR 161 [ HH ] |
| 5 | A:ASN 363 [HD22] | 1,79 | B:GLU 399 [ OE1] |  | 5 | A:SER 142 [ OG ] | 1,96 | B:ARG 164 [HH12] |
| 6 | A:GLU 391 [ OE2] | 1,57 | B:THR 374 [ HG1] |  | 6 | A:SER 142 [ O  ] | 2,46 | B:ARG 164 [HH12] |
| 7 | A:ALA 383 [ O  ] | 2,06 | B:LYS 379 [ HZ1] |  | 7 | A:SER 142 [ O  ] | 2,12 | B:ARG 164 [HH22] |
| 8 | A:THR 388 [ OG1] | 1,81 | B:LYS 379 [ HZ3] |  | 8 | A:THR 143 [ OG1] | 1,82 | B:SER 160 [ HG ] |
| 9 | A:ALA 367 [ O  ] | 2,05 | B:TYR 392 [ HH ] |  | 9 | A:CYS 156 [ SG ] | 2,46 | B:ARG 148 [HH11] |
| 10 | A:SER 373 [ OG ] | 1,84 | B:ARG 395 [HH11] |  | 10 | A:THR 157 [ OG1] | 1,93 | B:ARG 148 [HH12] |
|  |  |  |  |  |  |  |  |  |
| Salt bridges | | | |  | Salt bridges | | | |
| No salt bridges found | | | |  |  | Monomer 1 | Length | Monomer 2 |
|  |  |  |  |  | 1 | A:ARG 148 [ NE ] | 3,49 | B:ASP 154 [ OD2] |
|  |  |  |  |  | 2 | A:ARG 148 [ NH2] | 2,71 | B:ASP 154 [ OD2] |

| **StHK4sm-StHK4sm** | | | |
| --- | --- | --- | --- |
|  |  |  |  |
| Hydrogen bonds | | | |
|  | Monomer 1 | Length | Monomer 2 |
| 1 | A:ARG 120 [HH22] | 1,91 | B:SER  98 [ OG ] |
| 2 | A:THR  99 [ HG1] | 1,56 | B:GLU 116 [ OE1] |
| 3 | A:SER  98 [ OG ] | 1,83 | B:ARG 120 [HH12] |
| 4 | A:ALA 108 [ O  ] | 2,50 | B:LYS 104 [ HZ1] |
| 5 | A:THR 113 [ OG1] | 1,83 | B:LYS 104 [ HZ3] |
|  |  |  |  |
| Salt bridges | | | |
|  | Monomer 1 | Length | Monomer 2 |
| 1 | A:ASP 110 [ OD2] | 3,76 | B:LYS 104 [ NZ ] |

**HK_HisKA_-HK_HisKA_ complexes**

| **AHK2_HisKA_-AHK2_HisKA_** | | | |  | **AHK2_HisKA_-AHK3_HisKA_** | | | |
| --- | --- | --- | --- | --- | --- | --- | --- | --- |
|  |  |  |  |  |  |  |  |  |
| Hydrogen bonds | | | |  | Hydrogen bonds | | | |
|  | Monomer 1 | Length | Monomer 2 |  |  | Monomer 1 | Length | Monomer 2 |
| 1 | A:THR 616 [ HG1] | 1,88 | B:GLN 622 [ OE1] |  | 1 | A:LYS 588 [ HZ1] | 2,12 | B:ASP 448 [ O  ] |
| 2 | A:GLN 622 [HE21] | 1,90 | B:ASP 617 [ O  ] |  | 2 | A:GLN 622 [HE21] | 1,89 | B:THR 479 [ OG1] |
| 3 | A:GLN 622 [HE22] | 2,03 | B:THR 616 [ OG1] |  | 3 | A:GLN 622 [HE22] | 1,86 | B:GLU 480 [ O  ] |
| 4 | A:LYS 588 [ O  ] | 2,23 | B:GLN 646 [HE21] |  | 4 | A:TYR 625 [ HH ] | 2,43 | B:MET 475 [ SD ] |
|  |  |  |  |  | 5 | A:LYS 648 [ O  ] | 1,93 | B:LYS 451 [ HZ3] |
| Salt bridges | | | |  | 6 | A:THR 616 [ OG1] | 2,01 | B:GLN 485 [HE21] |
|  | Monomer 1 | Length | Monomer 2 |  | 7 | A:ASP 617 [ O  ] | 1,99 | B:GLN 485 [HE22] |
| 1 | A:ARG 563 [ NH1] | 3,36 | B:GLU 568 [ OE2] |  | 8 | A:LYS 588 [ O  ] | 2,42 | B:GLN 509 [HE22] |
| 2 | A:ARG 563 [ NH2] | 2,69 | B:GLU 568 [ OE2] |  |  |  |  |  |
| 3 | A:LYS 588 [ NZ ] | 2,67 | B:ASP 585 [ OD1] |  | Salt bridges | | | |
| 4 | A:LYS 588 [ NZ ] | 3,75 | B:ASP 585 [ OD2] |  |  | Monomer 1 | Length | Monomer 2 |
| 5 | A:GLU 568 [ OE2] | 3,86 | B:ARG 563 [ NE ] |  | 1 | A:ARG 563 [ NE ] | 2,80 | B:GLU 431 [ OE1] |
| 6 | A:GLU 568 [ OE1] | 2,78 | B:ARG 563 [ NH2] |  | 2 | A:ARG 563 [ NH2] | 2,82 | B:GLU 431 [ OE1] |
| 7 | A:GLU 568 [ OE2] | 2,68 | B:ARG 563 [ NH2] |  | 3 | A:ARG 563 [ NH2] | 2,75 | B:GLU 431 [ OE2] |
| 8 | A:GLU 650 [ OE1] | 2,62 | B:LYS 588 [ NZ ] |  | 4 | A:LYS 588 [ NZ ] | 2,71 | B:GLU 513 [ OE2] |
| 9 | A:GLU 650 [ OE2] | 3,31 | B:LYS 588 [ NZ ] |  | 5 | A:LYS 588 [ NZ ] | 2,70 | B:ASP 448 [ OD1] |
|  |  |  |  |  | 6 | A:GLU 568 [ OE1] | 3,36 | B:ARG 426 [ NH1] |
|  |  |  |  |  | 7 | A:GLU 650 [ OE2] | 2,72 | B:LYS 451 [ NZ ] |
|  |  |  |  |  |  |  |  |  |
| **AHK2_HisKA_-AHK4_HisKA_** | | | |  | **AHK3_HisKA_-AHK3_HisKA_** | | | |
|  |  |  |  |  |  |  |  |  |
| Hydrogen bonds | | | |  | Hydrogen bonds | | | |
|  | Monomer 1 | Length | Monomer 2 |  |  | Monomer 1 | Length | Monomer 2 |
| 1 | A:LYS 588 [ HZ1] | 2,33 | B:ASP 470 [ O  ] |  | 1 | A:GLN 485 [HE21] | 2,10 | B:THR 479 [ OG1] |
| 2 | A:GLN 622 [HE21] | 1,90 | B:THR 501 [ OG1] |  | 2 | A:GLN 485 [HE22] | 1,94 | B:GLU 480 [ O  ] |
| 3 | A:GLN 622 [HE22] | 1,91 | B:GLU 502 [ O  ] |  | 3 | A:GLN 509 [ O  ] | 1,85 | B:LYS 451 [ HZ1] |
| 4 | A:SER 632 [ HG ] | 1,97 | B:PRO 487 [ O  ] |  | 4 | A:LYS 511 [ O  ] | 1,91 | B:LYS 451 [ HZ2] |
| 5 | A:LYS 648 [ O  ] | 1,70 | B:LYS 473 [ HZ3] |  | 5 | A:GLU 480 [ O  ] | 2,04 | B:GLN 485 [HE22] |
| 6 | A:ASP 617 [ O  ] | 1,93 | B:GLN 507 [HE22] |  | 6 | A:THR 479 [ OG1] | 1,99 | B:GLN 485 [HE21] |
| 7 | A:THR 616 [ OG1] | 1,90 | B:GLN 507 [HE21] |  | 7 | A:PRO 465 [ O  ] | 1,93 | B:SER 495 [ HG ] |
|  |  |  |  |  |  |  |  |  |
| Salt bridges | | | |  | Salt bridges | | | |
|  | Monomer 1 | Length | Monomer 2 |  |  | Monomer 1 | Length | Monomer 2 |
| 1 | A:ARG 563 [ NE ] | 2,99 | B:GLU 453 [ OE2] |  | 1 | A:LYS 429 [ NZ ] | 2,67 | B:GLU 431 [ OE2] |
| 2 | A:ARG 563 [ NH2] | 2,76 | B:GLU 453 [ OE1] |  | 2 | A:LYS 451 [ NZ ] | 2,68 | B:GLU 513 [ OE2] |
| 3 | A:ARG 563 [ NH2] | 2,73 | B:GLU 453 [ OE2] |  | 3 | A:LYS 451 [ NZ ] | 2,85 | B:ASP 448 [ OD1] |
| 4 | A:ARG 575 [ NE ] | 3,64 | B:ASP 455 [ OD1] |  | 4 | A:LYS 451 [ NZ ] | 2,76 | B:ASP 448 [ OD2] |
| 5 | A:ARG 575 [ NH2] | 2,76 | B:ASP 455 [ OD1] |  | 5 | A:GLU 431 [ OE1] | 2,71 | B:LYS 429 [ NZ ] |
| 6 | A:ARG 575 [ NH2] | 3,34 | B:ASP 455 [ OD2] |  | 6 | A:GLU 513 [ OE1] | 2,71 | B:LYS 451 [ NZ ] |
| 7 | A:LYS 578 [ NZ ] | 2,69 | B:GLU 458 [ OE2] |  |  |  |  |  |
| 8 | A:LYS 588 [ NZ ] | 2,69 | B:ASP 470 [ OD1] |  |  |  |  |  |
| 9 | A:LYS 588 [ NZ ] | 3,60 | B:GLU 535 [ OE2] |  |  |  |  |  |
| 10 | A:GLU 568 [ OE1] | 2,74 | B:HIS 448 [ NE2] |  |  |  |  |  |
| 11 | A:GLU 650 [ OE2] | 2,72 | B:LYS 473 [ NZ ] |  |  |  |  |  |
| 12 | A:GLU 650 [ OE1] | 2,79 | B:LYS 473 [ NZ ] |  |  |  |  |  |

| **AHK3_HisKA_-AHK4_HisKA_** | | | |  | **AHK4_HisKA_-AHK4_HisKA_** | | | |
| --- | --- | --- | --- | --- | --- | --- | --- | --- |
|  |  |  |  |  |  |  |  |  |
| Hydrogen bonds | | | |  | Hydrogen bonds | | | |
|  | Monomer 1 | Length | Monomer 2 |  |  | Monomer 1 | Length | Monomer 2 |
| 1 | A:LYS 451 [ HZ1] | 2,15 | B:SER 474 [ OG ] |  | 1 | A:GLN 507 [HE21] | 1,85 | B:THR 501 [ OG1] |
| 2 | A:GLN 485 [HE22] | 2,45 | B:GLU 502 [ O  ] |  | 2 | A:GLN 507 [HE22] | 1,86 | B:GLU 502 [ O  ] |
| 3 | A:THR 479 [ OG1] | 1,90 | B:GLN 507 [HE21] |  | 3 | A:GLU 453 [ OE1] | 2,10 | B:PHE 436 [ H  ] |
| 4 | A:GLU 480 [ O  ] | 1,87 | B:GLN 507 [HE22] |  | 4 | A:ARG 531 [ O  ] | 1,80 | B:LYS 473 [ HZ3] |
|  |  |  |  |  | 5 | A:LYS 533 [ O  ] | 1,78 | B:LYS 473 [ HZ2] |
| Salt bridges | | | |  | 6 | A:GLU 502 [ O  ] | 1,94 | B:GLN 507 [HE22] |
|  | Monomer 1 | Length | Monomer 2 |  | 7 | A:THR 501 [ OG1] | 1,84 | B:GLN 507 [HE21] |
| 1 | A:ARG 426 [ NE ] | 2,86 | B:GLU 453 [ OE2] |  |  |  |  |  |
| 2 | A:ARG 426 [ NH2] | 2,80 | B:GLU 453 [ OE1] |  | Salt bridges | | | |
| 3 | A:ARG 426 [ NH2] | 2,70 | B:GLU 453 [ OE2] |  |  | Monomer 1 | Length | Monomer 2 |
| 4 | A:LYS 443 [ NZ ] | 3,84 | B:GLU 535 [ OE2] |  | 1 | A:HIS 448 [ NE2] | 2,80 | B:GLU 453 [ OE2] |
| 5 | A:LYS 451 [ NZ ] | 2,92 | B:ASP 470 [ OD1] |  | 2 | A:HIS 448 [ NE2] | 3,47 | B:GLU 453 [ OE1] |
| 6 | A:LYS 451 [ NZ ] | 2,68 | B:ASP 470 [ OD2] |  | 3 | A:LYS 473 [ NZ ] | 3,25 | B:ASP 470 [ OD2] |
|  |  |  |  |  | 4 | A:LYS 473 [ NZ ] | 2,71 | B:ASP 470 [ OD1] |
|  |  |  |  |  |  |  |  |  |
| **StHK2_HisKA_-StHK2_HisKA_** | | | |  | **StHK3_HisKA_-StHK3_HisKA_** | | | |
|  |  |  |  |  |  |  |  |  |
| Hydrogen bonds | | | |  | Hydrogen bonds | | | |
|  | Monomer 1 | Length | Monomer 2 |  |  | Monomer 1 | Length | Monomer 2 |
| 1 | A:LYS 666 [ HZ1] | 1,83 | B:SER 667 [ OG ] |  | 1 | A:GLN 472 [HE21] | 1,97 | B:THR 466 [ OG1] |
| 2 | A:GLN 700 [HE22] | 1,92 | B:THR 694 [ OG1] |  | 2 | A:GLN 472 [HE22] | 1,97 | B:ASN 467 [ O  ] |
| 3 | A:GLN 700 [HE21] | 2,02 | B:ASN 695 [ O  ] |  | 3 | A:THR 466 [ HG1] | 1,86 | B:GLN 472 [ OE1] |
| 4 | A:LYS 726 [ O  ] | 2,43 | B:LYS 666 [ HZ2] |  | 4 | A:LYS 438 [ HZ2] | 2,19 | B:GLN 496 [ OE1] |
| 5 | A:SER 729 [ OG ] | 2,30 | B:LYS 666 [ HZ1] |  | 5 | A:MET 424 [ SD ] | 2,33 | B:LYS 428 [ HZ3] |
| 6 | A:ASN 695 [ O  ] | 2,24 | B:GLN 700 [HE21] |  | 6 | A:GLN 496 [ O  ] | 1,93 | B:LYS 438 [ HZ2] |
|  |  |  |  |  | 7 | A:LYS 498 [ O  ] | 1,75 | B:LYS 438 [ HZ3] |
| Salt bridges | | | |  |  |  |  |  |
|  | Monomer 1 | Length | Monomer 2 |  | Salt bridges | | | |
| 1 | A:LYS 666 [ NZ ] | 2,68 | B:ASP 663 [ OD2] |  |  | Monomer 1 | Length | Monomer 2 |
| 2 | A:LYS 666 [ NZ ] | 3,81 | B:GLU 728 [ OE1] |  | 1 | A:LYS 438 [ NZ ] | 2,69 | B:ASP 435 [ OD1] |
| 3 | A:LYS 666 [ NZ ] | 2,76 | B:GLU 728 [ OE2] |  | 2 | A:GLU 418 [ OE2] | 3,86 | B:ARG 413 [ NE ] |
| 4 | A:LYS 666 [ NZ ] | 2,73 | B:ASP 663 [ OD1] |  | 3 | A:GLU 418 [ OE1] | 2,76 | B:ARG 413 [ NE ] |
| 5 | A:GLU 646 [ OE2] | 3,77 | B:ARG 641 [ NE ] |  | 4 | A:GLU 418 [ OE2] | 2,71 | B:ARG 413 [ NH2] |
| 6 | A:GLU 646 [ OE1] | 3,03 | B:ARG 641 [ NH1] |  | 5 | A:GLU 418 [ OE1] | 3,04 | B:ARG 413 [ NH2] |
| 7 | A:GLU 646 [ OE2] | 2,74 | B:ARG 641 [ NH1] |  | 6 | A:GLU 423 [ OE2] | 3,52 | B:LYS 428 [ NZ ] |
| 8 | A:GLU 728 [ OE1] | 2,66 | B:LYS 666 [ NZ ] |  | 7 | A:GLY 502 [ O  ] | 2,83 | B:ARG 430 [ NE ] |
|  |  |  |  |  | 8 | A:GLY 502 [ OXT] | 3,45 | B:ARG 430 [ NE ] |
|  |  |  |  |  | 9 | A:GLY 502 [ O  ] | 3,92 | B:ARG 430 [ NH1] |
|  |  |  |  |  | 10 | A:GLY 502 [ OXT] | 3,10 | B:ARG 430 [ NH1] |
|  |  |  |  |  | 11 | A:GLY 502 [ O  ] | 2,73 | B:ARG 430 [ NH2] |
|  |  |  |  |  | 12 | A:GLY 502 [ OXT] | 2,81 | B:ARG 430 [ NH2] |
|  |  |  |  |  | 13 | A:GLU 500 [ OE1] | 2,74 | B:LYS 438 [ NZ ] |
|  |  |  |  |  | 14 | A:GLU 500 [ OE2] | 2,94 | B:LYS 438 [ NZ ] |

| **StHK4_HisKA_-StHK4_HisKA_** | | | |
| --- | --- | --- | --- |
|  |  |  |  |
| Hydrogen bonds | | | |
|  | Monomer 1 | Length | Monomer 2 |
| 1 | A:GLN 413 [HE21] | 1,89 | B:THR 407 [ OG1] |
| 2 | A:GLN 413 [HE22] | 1,96 | B:ASP 408 [ O  ] |
| 3 | A:LYS 439 [ O  ] | 1,79 | B:LYS 379 [ HZ3] |
| 4 | A:ASP 408 [ O  ] | 1,93 | B:GLN 413 [HE22] |
| 5 | A:THR 407 [ OG1] | 1,84 | B:GLN 413 [HE21] |
|  |  |  |  |
| Salt bridges | | | |
|  | Monomer 1 | Length | Monomer 2 |
| 1 | A:HIS 354 [ NE2] | 2,95 | B:GLU 359 [ OE1] |
| 2 | A:LYS 379 [ NZ ] | 2,76 | B:ASP 376 [ OD1] |
| 3 | A:LYS 379 [ NZ ] | 2,77 | B:ASP 376 [ OD2] |
| 4 | A:GLU 359 [ OE1] | 2,73 | B:HIS 354 [ NE2] |
| 5 | A:GLU 441 [ OE1] | 2,69 | B:LYS 379 [ NZ ] |
| 6 | A:GLU 441 [ OE2] | 2,79 | B:LYS 379 [ NZ ] |
| 7 | A:GLU 389 [ OE1] | 3,54 | B:ARG 429 [ NH1] |
| 8 | A:GLU 389 [ OE2] | 2,71 | B:ARG 429 [ NH1] |
| 9 | A:GLU 389 [ OE1] | 2,85 | B:ARG 429 [ NH2] |
| 10 | A:GLU 389 [ OE2] | 3,53 | B:ARG 429 [ NH2] |

**HKrd-HPt complexes**

| **AHK2rd-AHP1** | | | |  | **AHK2rd-AHP2** | | | |
| --- | --- | --- | --- | --- | --- | --- | --- | --- |
|  |  |  |  |  |  |  |  |  |
| Hydrogen bonds | | | |  | Hydrogen bonds | | | |
|  | Monomer 1 | Length | Monomer 2 |  |  | Monomer 1 | Length | Monomer 2 |
| 1 | A:ASN1043 [HD21] | 1,89 | B:GLN  80 [ OE1] |  | 1 | A:ASN1043 [HD21] | 2,02 | B:GLN  83 [ OE1] |
| 2 | A:ASN1043 [HD22] | 1,88 | B:SER  84 [ OG ] |  | 2 | A:ASN1043 [HD22] | 1,87 | B:SER  87 [ OG ] |
| 3 | A:ASN1046 [HD21] | 2,06 | B:GLY  83 [ O  ] |  | 3 | A:ASN1046 [HD22] | 1,99 | B:GLY  86 [ O  ] |
| 4 | A:ARG1048 [HH21] | 2,06 | B:GLN  44 [ OE1] |  | 4 | A:LYS1056 [ HZ1] | 1,95 | B:LEU  35 [ O  ] |
| 5 | A:LYS1056 [ HZ1] | 2,43 | B:GLN  32 [ O  ] |  | 5 | A:LYS1056 [ HZ3] | 2,27 | B:SER  40 [ OG ] |
| 6 | A:LYS1056 [ HZ1] | 2,13 | B:LEU  33 [ O  ] |  | 6 | A:ALA1137 [ O  ] | 1,89 | B:LYS  85 [ HZ3] |
| 7 | A:GLU1162 [ H  ] | 1,91 | B:GLN  29 [ OE1] |  | 7 | A:ASP1138 [ O  ] | 2,20 | B:LYS  85 [ HZ2] |
| 8 | A:ASN1046 [ OD1] | 1,64 | B:SER  87 [ HG ] |  | 8 | A:PHE1160 [ O  ] | 1,93 | B:GLN  28 [HE21] |
| 9 | A:ALA1137 [ O  ] | 1,85 | B:LYS  82 [ HZ1] |  |  |  |  |  |
| 10 | A:ASP1138 [ O  ] | 2,05 | B:LYS  82 [ HZ3] |  | Salt bridges | | | |
| 11 | A:GLN1141 [ OE1] | 1,83 | B:ARG 101 [HH12] |  |  | Monomer 1 | Length | Monomer 2 |
| 12 | A:PHE1160 [ O  ] | 1,91 | B:GLN  26 [HE22] |  | 1 | A:ARG1048 [ NE ] | 3,79 | B:GLU  46 [ OE1] |
| 13 | A:GLU1162 [ OE2] | 1,84 | B:GLN  32 [HE22] |  | 2 | A:ARG1048 [ NE ] | 2,73 | B:GLU  46 [ OE2] |
|  |  |  |  |  | 3 | A:ARG1048 [ NH2] | 2,73 | B:GLU  46 [ OE1] |
| Salt bridges | | | |  | 4 | A:ARG1048 [ NH2] | 3,21 | B:GLU  46 [ OE2] |
|  | Monomer 1 | Length | Monomer 2 |  | 5 | A:LYS1056 [ NZ ] | 2,71 | B:ASP  37 [ OD2] |
| 1 | A:LYS1056 [ NZ ] | 2,65 | B:ASP  35 [ OD2] |  | 6 | A:ASP1138 [ OD2] | 2,73 | B:LYS  85 [ NZ ] |
| 2 | A:ASP1138 [ OD2] | 2,67 | B:LYS  82 [ NZ ] |  | 7 | A:GLU1162 [ OE2] | 2,77 | B:LYS  34 [ NZ ] |
|  |  |  |  |  |  |  |  |  |
| **AHK2rd-AHP3** | | | |  | **AHK3rd-AHP1** | | | |
|  |  |  |  |  |  |  |  |  |
| Hydrogen bonds | | | |  | Hydrogen bonds | | | |
|  | Monomer 1 | Length | Monomer 2 |  |  | Monomer 1 | Length | Monomer 2 |
| 1 | A:ASN1043 [HD21] | 1,95 | B:GLN  83 [ OE1] |  | 1 | A:ASN 901 [HD22] | 2,21 | B:SER  87 [ OG ] |
| 2 | A:ASN1043 [HD22] | 1,88 | B:SER  87 [ OG ] |  | 2 | A:LYS 911 [ HZ1] | 2,22 | B:LEU  33 [ O  ] |
| 3 | A:ASN1046 [HD22] | 1,89 | B:GLY  86 [ O  ] |  | 3 | A:ALA1017 [ H  ] | 1,90 | B:GLN  29 [ OE1] |
| 4 | A:LYS1056 [ HZ1] | 1,78 | B:LEU  35 [ O  ] |  | 4 | A:GLN 943 [ O  ] | 2,18 | B:HIS  79 [ HE2] |
| 5 | A:GLU1162 [ H  ] | 1,95 | B:GLU  31 [ OE1] |  | 5 | A:ALA 992 [ O  ] | 1,82 | B:LYS  82 [ HZ2] |
| 6 | A:ALA1137 [ O  ] | 1,92 | B:LYS  85 [ HZ2] |  | 6 | A:ASP 993 [ O  ] | 2,14 | B:LYS  82 [ HZ1] |
| 7 | A:ASP1138 [ O  ] | 2,45 | B:LYS  85 [ HZ1] |  | 7 | A:LYS1013 [ O  ] | 1,99 | B:SER  87 [ HG ] |
| 8 | A:ASP1138 [ O  ] | 2,31 | B:LYS  85 [ HZ3] |  | 8 | A:PHE1015 [ O  ] | 1,87 | B:GLN  26 [HE21] |
| 9 | A:PHE1160 [ O  ] | 1,87 | B:GLN  28 [HE22] |  |  |  |  |  |
|  |  |  |  |  | Salt bridges | | | |
| Salt bridges | | | |  |  | Monomer 1 | Length | Monomer 2 |
|  | Monomer 1 | Length | Monomer 2 |  | 1 | A:ASP 993 [ OD2] | 3,22 | B:LYS  82 [ NZ ] |
| 1 | A:ARG1048 [ NE ] | 3,58 | B:GLU  46 [ OE1] |  |  |  |  |  |
| 2 | A:ARG1048 [ NE ] | 3,41 | B:GLU  46 [ OE2] |  |  |  |  |  |
| 3 | A:ARG1048 [ NH2] | 3,25 | B:GLU  46 [ OE1] |  |  |  |  |  |
| 4 | A:ARG1048 [ NH2] | 2,81 | B:GLU  46 [ OE2] |  |  |  |  |  |
| 5 | A:LYS1056 [ NZ ] | 2,64 | B:ASP  37 [ OD2] |  |  |  |  |  |
| 6 | A:LYS1056 [ NZ ] | 3,41 | B:ASP  37 [ OD1] |  |  |  |  |  |
| 7 | A:ASP1138 [ OD1] | 2,68 | B:LYS  85 [ NZ ] |  |  |  |  |  |
| 8 | A:ASP1138 [ OD2] | 2,78 | B:LYS  85 [ NZ ] |  |  |  |  |  |
| 9 | A:GLU1162 [ OE1] | 2,68 | B:LYS  34 [ NZ ] |  |  |  |  |  |

| **AHK3rd-AHP2** | | | |  | **AHK3rd-AHP3** | | | |
| --- | --- | --- | --- | --- | --- | --- | --- | --- |
|  |  |  |  |  |  |  |  |  |
| Hydrogen bonds | | | |  | Hydrogen bonds | | | |
|  | Monomer 1 | Length | Monomer 2 |  |  | Monomer 1 | Length | Monomer 2 |
| 1 | A:ASN 898 [HD22] | 1,91 | B:GLN  83 [ OE1] |  | 1 | A:ASN 898 [HD22] | 1,89 | B:GLN  83 [ OE1] |
| 2 | A:ASN 898 [HD21] | 1,86 | B:SER  87 [ OG ] |  | 2 | A:ASN 898 [HD21] | 1,88 | B:SER  87 [ OG ] |
| 3 | A:ASN 899 [HD21] | 1,87 | B:ASP  54 [ OD2] |  | 3 | A:ASN 901 [HD22] | 2,04 | B:GLY  86 [ O  ] |
| 4 | A:ASN 901 [HD22] | 2,25 | B:SER  90 [ OG ] |  | 4 | A:LYS 911 [ HZ2] | 2,28 | B:LEU  35 [ O  ] |
| 5 | A:ASN 901 [HD21] | 1,92 | B:GLY  86 [ O  ] |  | 5 | A:LYS 911 [ HZ2] | 1,95 | B:SER  40 [ OG ] |
| 6 | A:ALA1017 [ H  ] | 2,16 | B:GLU  31 [ OE1] |  | 6 | A:ALA1017 [ H  ] | 1,86 | B:GLU  31 [ OE2] |
| 7 | A:ALA 992 [ O  ] | 1,87 | B:LYS  85 [ HZ3] |  | 7 | A:ASN 901 [ OD1] | 1,68 | B:SER  90 [ HG ] |
| 8 | A:ASP 993 [ O  ] | 2,15 | B:LYS  85 [ HZ2] |  | 8 | A:ALA 992 [ O  ] | 1,78 | B:LYS  85 [ HZ3] |
| 9 | A:PHE1015 [ O  ] | 1,85 | B:GLN  28 [HE22] |  | 9 | A:PHE1015 [ O  ] | 1,90 | B:GLN  28 [HE22] |
|  |  |  |  |  |  |  |  |  |
| Salt bridges | | | |  | Salt bridges | | | |
|  | Monomer 1 | Length | Monomer 2 |  |  | Monomer 1 | Length | Monomer 2 |
| 1 | A:ARG 903 [ NH1] | 3,60 | B:GLU  46 [ OE2] |  | 1 | A:ARG 903 [ NE ] | 3,81 | B:GLU  46 [ OE1] |
| 2 | A:ARG 903 [ NH1] | 2,71 | B:GLU  46 [ OE1] |  | 2 | A:ARG 903 [ NH1] | 2,70 | B:GLU  46 [ OE1] |
| 3 | A:ARG 903 [ NH2] | 2,68 | B:GLU  46 [ OE2] |  | 3 | A:ARG 903 [ NH1] | 2,68 | B:GLU  46 [ OE2] |
| 4 | A:ARG 903 [ NH2] | 3,35 | B:GLU  46 [ OE1] |  | 4 | A:ARG 903 [ NH2] | 3,19 | B:GLU  46 [ OE2] |
| 5 | A:LYS 911 [ NZ ] | 2,62 | B:ASP  37 [ OD2] |  | 5 | A:LYS 911 [ NZ ] | 2,83 | B:ASP  37 [ OD1] |
| 6 | A:ASP 993 [ OD2] | 2,72 | B:LYS  85 [ NZ ] |  | 6 | A:LYS 911 [ NZ ] | 2,72 | B:ASP  37 [ OD2] |
|  |  |  |  |  | 7 | A:ASP 993 [ OD2] | 2,67 | B:LYS  85 [ NZ ] |
|  |  |  |  |  |  |  |  |  |
| **AHK4rd-AHP1** | | | |  | **AHK4rd-AHP2** | | | |
|  |  |  |  |  |  |  |  |  |
| Hydrogen bonds | | | |  | Hydrogen bonds | | | |
|  | Monomer 1 | Length | Monomer 2 |  |  | Monomer 1 | Length | Monomer 2 |
| 1 | A:ASN 953 [HD21] | 1,94 | B:GLN  80 [ OE1] |  | 1 | A:ASN 953 [HD22] | 1,85 | B:SER  87 [ OG ] |
| 2 | A:ASN 953 [HD22] | 1,88 | B:SER  84 [ OG ] |  | 2 | A:ASN 956 [HD22] | 2,26 | B:GLY  86 [ O  ] |
| 3 | A:ASN 956 [HD22] | 1,95 | B:GLY  83 [ O  ] |  | 3 | A:ALA1035 [ O  ] | 1,95 | B:LYS  85 [ HZ2] |
| 4 | A:ARG 958 [HH12] | 1,85 | B:GLN  44 [ OE1] |  | 4 | A:PHE1058 [ O  ] | 1,89 | B:GLN  28 [HE21] |
| 5 | A:ARG 958 [HH11] | 1,79 | B:GLN  44 [ O  ] |  | 5 | A:GLU1060 [ OE2] | 2,27 | B:ASN 156 [HD22] |
| 6 | A:LYS 966 [ HZ3] | 1,97 | B:LEU  33 [ O  ] |  |  |  |  |  |
| 7 | A:GLU1060 [ H  ] | 2,46 | B:GLN  29 [ OE1] |  | Salt bridges | | | |
| 8 | A:ALA1035 [ O  ] | 1,72 | B:LYS  82 [ HZ1] |  |  | Monomer 1 | Length | Monomer 2 |
| 9 | A:ASP1036 [ O  ] | 2,06 | B:LYS  82 [ HZ2] |  | 1 | A:ARG 958 [ NE ] | 3,72 | B:GLU  46 [ OE2] |
| 10 | A:PHE1058 [ O  ] | 1,86 | B:GLN  26 [HE22] |  | 2 | A:ARG 958 [ NE ] | 2,75 | B:GLU  46 [ OE1] |
| 11 | A:GLU1059 [ OE2] | 2,04 | B:GLN  29 [HE22] |  | 3 | A:ARG 958 [ NH2] | 2,74 | B:GLU  46 [ OE2] |
|  |  |  |  |  | 4 | A:ARG 958 [ NH2] | 2,73 | B:GLU  46 [ OE1] |
| Salt bridges | | | |  | 5 | A:LYS 966 [ NZ ] | 2,72 | B:ASP  37 [ OD1] |
|  | Monomer 1 | Length | Monomer 2 |  | 6 | A:LYS 966 [ NZ ] | 2,79 | B:ASP  37 [ OD2] |
| 1 | A:LYS 966 [ NZ ] | 2,62 | B:ASP  35 [ OD1] |  |  |  |  |  |

| **AHK4rd-AHP3** | | | |  | **AHK5(CKI1)rd-AHP1** | | | |
| --- | --- | --- | --- | --- | --- | --- | --- | --- |
|  |  |  |  |  |  |  |  |  |
| Hydrogen bonds | | | |  | Hydrogen bonds | | | |
|  | Monomer 1 | Length | Monomer 2 |  |  | Monomer 1 | Length | Monomer 2 |
| 1 | A:ASN 953 [HD22] | 1,84 | B:SER  87 [ OG ] |  | 1 | A:ASN 786 [HD21] | 1,91 | B:SER  84 [ OG ] |
| 2 | A:ASN 953 [HD21] | 1,93 | B:GLN  83 [ OE1] |  | 2 | A:ASN 786 [HD22] | 1,89 | B:GLN  80 [ OE1] |
| 3 | A:ASN 956 [HD22] | 1,87 | B:GLY  86 [ O  ] |  | 3 | A:ASN 789 [HD22] | 2,20 | B:GLY  83 [ O  ] |
| 4 | A:LYS 966 [ HZ3] | 1,87 | B:LEU  35 [ O  ] |  | 4 | A:GLN 799 [HE22] | 1,90 | B:GLN  32 [ O  ] |
| 5 | A:GLU1060 [ H  ] | 1,89 | B:GLU  31 [ OE2] |  | 5 | A:LEU 910 [ H  ] | 1,85 | B:GLN  29 [ OE1] |
| 6 | A:ASN 956 [ OD1] | 1,73 | B:SER  90 [ HG ] |  | 6 | A:ASN 789 [ OD1] | 1,61 | B:SER  87 [ HG ] |
| 7 | A:ALA1035 [ O  ] | 2,07 | B:LYS  85 [ HZ3] |  | 7 | A:ALA 885 [ O  ] | 1,93 | B:LYS  82 [ HZ3] |
| 8 | A:ASP1036 [ O  ] | 1,82 | B:LYS  85 [ HZ2] |  | 8 | A:ASN 886 [ O  ] | 1,76 | B:LYS  82 [ HZ1] |
| 9 | A:PHE1058 [ O  ] | 1,84 | B:GLN  28 [HE22] |  | 9 | A:VAL 908 [ O  ] | 1,97 | B:GLN  26 [HE21] |
|  |  |  |  |  |  |  |  |  |
| Salt bridges | | | |  | Salt bridges | | | |
|  | Monomer 1 | Length | Monomer 2 |  |  | Monomer 1 | Length | Monomer 2 |
| 1 | A:ARG 958 [ NE ] | 3,82 | B:GLU  46 [ OE2] |  | 1 | A:GLU 890 [ OE2] | 3,64 | B:LYS  82 [ NZ ] |
| 2 | A:ARG 958 [ NH1] | 2,66 | B:GLU  46 [ OE2] |  |  |  |  |  |
| 3 | A:LYS 966 [ NZ ] | 3,15 | B:ASP  37 [ OD1] |  |  |  |  |  |
| 4 | A:LYS 966 [ NZ ] | 2,74 | B:ASP  37 [ OD2] |  |  |  |  |  |
| 5 | A:ASP1036 [ OD2] | 2,72 | B:LYS  85 [ NZ ] |  |  |  |  |  |
| 6 | A:GLU1060 [ OE1] | 2,74 | B:LYS  34 [ NZ ] |  |  |  |  |  |
| 7 | A:GLU1061 [ OE1] | 3,12 | B:LYS  34 [ NZ ] |  |  |  |  |  |
| 8 | A:GLU1061 [ OE2] | 2,73 | B:LYS  34 [ NZ ] |  |  |  |  |  |
|  |  |  |  |  |  |  |  |  |
| **StHK2rd-StHP1a** | | | |  | **StHK3rd-StHP1a** | | | |
|  |  |  |  |  |  |  |  |  |
| Hydrogen bonds | | | |  | Hydrogen bonds | | | |
|  | Monomer 1 | Length | Monomer 2 |  |  | Monomer 1 | Length | Monomer 2 |
| 1 | A:ASN1130 [HD22] | 1,89 | B:SER  84 [ OG ] |  | 1 | A:ASN 894 [HD21] | 1,93 | B:GLN  80 [ OE1] |
| 2 | A:ASN1130 [HD21] | 1,89 | B:GLN  80 [ OE1] |  | 2 | A:ASN 894 [HD22] | 1,93 | B:SER  84 [ OG ] |
| 3 | A:ASN1133 [HD22] | 2,02 | B:GLY  83 [ O  ] |  | 3 | A:ASN 897 [HD22] | 2,10 | B:GLY  83 [ O  ] |
| 4 | A:LYS1142 [ HZ2] | 2,38 | B:ASN  38 [ OD1] |  | 4 | A:LYS 907 [ HZ2] | 2,12 | B:GLN  32 [ O  ] |
| 5 | A:LYS1143 [ HZ1] | 2,03 | B:SER  37 [ OG ] |  | 5 | A:LYS 907 [ HZ3] | 1,81 | B:LEU  33 [ O  ] |
| 6 | A:LYS1249 [ HZ3] | 1,77 | B:GLN  32 [ O  ] |  | 6 | A:ALA 988 [ O  ] | 1,89 | B:LYS  82 [ HZ3] |
| 7 | A:GLN1175 [ O  ] | 2,03 | B:HIS  79 [ HE2] |  | 7 | A:ASP 989 [ O  ] | 2,24 | B:LYS  82 [ HZ2] |
| 8 | A:ALA1224 [ O  ] | 1,83 | B:LYS  82 [ HZ1] |  | 8 | A:PHE1011 [ O  ] | 1,90 | B:GLN  26 [HE22] |
| 9 | A:ASP1225 [ O  ] | 2,38 | B:LYS  82 [ HZ1] |  |  |  |  |  |
| 10 | A:ASP1225 [ O  ] | 2,24 | B:LYS  82 [ HZ3] |  | Salt bridges | | | |
| 11 | A:PHE1247 [ O  ] | 1,91 | B:GLN  26 [HE22] |  |  | Monomer 1 | Length | Monomer 2 |
|  |  |  |  |  | 1 | A:ARG 899 [ NH1] | 2,66 | B:GLU  44 [ OE1] |
| Salt bridges | | | |  | 2 | A:ARG 899 [ NH1] | 2,86 | B:GLU  44 [ OE2] |
|  | Monomer 1 | Length | Monomer 2 |  | 3 | A:ARG 899 [ NH2] | 3,80 | B:GLU  44 [ OE1] |
| 1 | A:ARG1135 [ NH1] | 3,68 | B:GLU  44 [ OE1] |  | 4 | A:ARG 899 [ NH2] | 2,72 | B:GLU  44 [ OE2] |
| 2 | A:ARG1135 [ NH1] | 2,69 | B:GLU  44 [ OE2] |  | 5 | A:LYS 907 [ NZ ] | 2,66 | B:ASP  35 [ OD2] |
| 3 | A:ARG1135 [ NH2] | 2,75 | B:GLU  44 [ OE1] |  | 6 | A:LYS 907 [ NZ ] | 3,93 | B:ASP  35 [ OD1] |
| 4 | A:ARG1135 [ NH2] | 2,94 | B:GLU  44 [ OE2] |  | 7 | A:ASP 989 [ OD2] | 2,70 | B:LYS  82 [ NZ ] |
| 5 | A:LYS1143 [ NZ ] | 2,71 | B:ASP  35 [ OD1] |  |  |  |  |  |
| 6 | A:LYS1143 [ NZ ] | 2,63 | B:ASP  35 [ OD2] |  |  |  |  |  |
| 7 | A:ASP1225 [ OD2] | 2,72 | B:LYS  82 [ NZ ] |  |  |  |  |  |

| **StHK4rd-StHP1a** | | | |
| --- | --- | --- | --- |
|  |  |  |  |
| Hydrogen bonds | | | |
|  | Monomer 1 | Length | Monomer 2 |
| 1 | A:ASN 855 [HD22] | 1,96 | B:SER  84 [ OG ] |
| 2 | A:ASN 855 [HD21] | 1,89 | B:GLN  80 [ OE1] |
| 3 | A:ASN 858 [HD22] | 1,86 | B:GLY  83 [ O  ] |
| 4 | A:LYS 868 [ HZ1] | 1,86 | B:LEU  33 [ O  ] |
| 5 | A:GLU 973 [ H  ] | 2,01 | B:GLN  29 [ OE1] |
| 6 | A:PHE 971 [ O  ] | 1,82 | B:GLN  26 [HE22] |
| 7 | A:GLU 972 [ OE1] | 1,80 | B:GLN  29 [HE21] |
|  |  |  |  |
| Salt bridges | | | |
|  | Monomer 1 | Length | Monomer 2 |
| 1 | A:ARG 856 [ NH1] | 2,67 | B:GLU  51 [ OE1] |
| 2 | A:ARG 856 [ NH1] | 3,89 | B:ASP  52 [ OD1] |
| 3 | A:ARG 856 [ NH2] | 2,77 | B:GLU  51 [ OE1] |
| 4 | A:ARG 856 [ NH2] | 3,14 | B:GLU  51 [ OE2] |
| 5 | A:ARG 860 [ NH1] | 2,69 | B:GLU  44 [ OE1] |
| 6 | A:ARG 860 [ NH1] | 2,82 | B:GLU  44 [ OE2] |
| 7 | A:ARG 860 [ NH2] | 3,52 | B:GLU  44 [ OE1] |
| 8 | A:LYS 868 [ NZ ] | 2,71 | B:ASP  35 [ OD2] |
| 9 | A:LYS 868 [ NZ ] | 3,13 | B:ASP  35 [ OD1] |

**HPt-HPt complexes**

| **AHP1-AHP1** | | | |  | **AHP1-AHP2** | | | |
| --- | --- | --- | --- | --- | --- | --- | --- | --- |
|  |  |  |  |  |  |  |  |  |
| Hydrogen bonds | | | |  | Hydrogen bonds | | | |
|  | Monomer 1 | Length | Monomer 2 |  |  | Monomer 1 | Length | Monomer 2 |
| 1 | A:ARG 101 [HH11] | 2,02 | B:LEU  33 [ O  ] |  | 1 | A:SER  37 [ HG ] | 1,82 | B:PHE  74 [ O  ] |
| 2 | A:ARG 101 [HH21] | 1,87 | B:LEU  33 [ O  ] |  | 2 | A:GLN  44 [HE22] | 1,97 | B:ASP  73 [ OD2] |
| 3 | A:GLN  80 [HE22] | 1,79 | B:ASP  52 [ OD2] |  | 3 | A:ARG 101 [HH22] | 1,94 | B:LEU  35 [ O  ] |
| 4 | A:GLN  44 [HE22] | 2,11 | B:ASP  70 [ OD2] |  | 4 | A:ASP  40 [ OD2] | 1,69 | B:SER  75 [ HG ] |
| 5 | A:ASN  38 [HD21] | 2,19 | B:CYS 104 [ O  ] |  | 5 | A:ASP  52 [ OD2] | 1,77 | B:GLN  83 [HE22] |
| 6 | A:GLU 105 [ OE1] | 1,92 | B:SER  37 [ H  ] |  | 6 | A:LEU  33 [ O  ] | 2,21 | B:LYS 104 [ HZ2] |
| 7 | A:GLU 105 [ OE2] | 1,82 | B:ASN  38 [HD22] |  | 7 | A:SER  37 [ O  ] | 1,76 | B:LYS 110 [ HZ1] |
| 8 | A:ASN  38 [ OD1] | 1,74 | B:LYS  72 [ HZ2] |  |  |  |  |  |
| 9 | A:ASP  52 [ OD2] | 2,06 | B:GLN  80 [HE22] |  | Salt bridges | | | |
| 10 | A:LEU  33 [ O  ] | 2,26 | B:ARG 101 [HH11] |  |  | Monomer 1 | Length | Monomer 2 |
| 11 | A:LEU  33 [ O  ] | 1,77 | B:ARG 101 [HH22] |  | 1 | A:ARG  55 [ NH1] | 3,90 | B:ASP  54 [ OD1] |
|  |  |  |  |  | 2 | A:ARG  55 [ NH2] | 2,73 | B:ASP  54 [ OD1] |
| Salt bridges | | | |  | 3 | A:LYS  73 [ NZ ] | 2,67 | B:GLU  46 [ OE2] |
|  | Monomer 1 | Length | Monomer 2 |  | 4 | A:LYS  73 [ NZ ] | 2,69 | B:GLU  46 [ OE1] |
| 1 | A:ARG 101 [ NE ] | 3,76 | B:GLU  36 [ OE1] |  | 5 | A:ARG 101 [ NE ] | 3,12 | B:ASP  38 [ OD2] |
| 2 | A:ARG 101 [ NH1] | 2,68 | B:GLU  36 [ OE1] |  | 6 | A:ARG 101 [ NH2] | 2,69 | B:ASP  38 [ OD2] |
| 3 | A:ARG  55 [ NH2] | 3,95 | B:ASP  52 [ OD1] |  | 7 | A:ASP  52 [ OD1] | 3,97 | B:LYS  57 [ NZ ] |
| 4 | A:ARG  55 [ NH1] | 3,68 | B:ASP  52 [ OD1] |  | 8 | A:ASP  52 [ OD2] | 2,71 | B:LYS  57 [ NZ ] |
| 5 | A:ARG  55 [ NH2] | 3,80 | B:ASP  52 [ OD2] |  | 9 | A:GLU  36 [ OE2] | 2,69 | B:LYS 104 [ NZ ] |
| 6 | A:ARG  55 [ NH1] | 2,70 | B:ASP  52 [ OD2] |  |  |  |  |  |
|  |  |  |  |  |  |  |  |  |
| **AHP1-AHP3** | | | |  | **AHP2-AHP2** | | | |
|  |  |  |  |  |  |  |  |  |
| Hydrogen bonds | | | |  | Hydrogen bonds | | | |
|  | Monomer 1 | Length | Monomer 2 |  |  | Monomer 1 | Length | Monomer 2 |
| 1 | A:SER  37 [ H  ] | 1,83 | B:ASP 108 [ OD1] |  | 1 | A:SER  75 [ HG ] | 1,58 | B:GLU  46 [ OE2] |
| 2 | A:GLN  44 [HE21] | 1,86 | B:ASP  73 [ OD2] |  | 2 | A:GLN  76 [HE21] | 1,81 | B:GLU  46 [ OE1] |
| 3 | A:LYS  72 [ HZ2] | 2,03 | B:SER  40 [ OG ] |  | 3 | A:GLN  83 [HE21] | 1,84 | B:ASP  54 [ OD2] |
| 4 | A:GLN  80 [HE22] | 1,87 | B:ASP  54 [ OD2] |  | 4 | A:GLN  83 [HE22] | 2,20 | B:LEU  50 [ O  ] |
| 5 | A:ARG 101 [HH22] | 1,88 | B:LEU  35 [ O  ] |  | 5 | A:LYS 104 [ HZ1] | 1,95 | B:LEU  35 [ O  ] |
| 6 | A:ASN  38 [ O  ] | 2,29 | B:LYS  75 [ HZ2] |  | 6 | A:LYS 104 [ HZ3] | 2,13 | B:ASP  38 [ O  ] |
| 7 | A:GLU  36 [ O  ] | 1,89 | B:LYS  75 [ HZ2] |  | 7 | A:GLU 108 [ OE1] | 2,30 | B:GLY  39 [ H  ] |
| 8 | A:GLN  44 [ O  ] | 1,91 | B:SER  79 [ HG ] |  | 8 | A:ASP  54 [ OD1] | 2,16 | B:GLN  83 [HE21] |
| 9 | A:ASP  52 [ OD2] | 1,80 | B:GLN  83 [HE22] |  | 9 | A:ASP  54 [ OD2] | 2,05 | B:GLN  83 [HE22] |
| 10 | A:GLN  80 [ OE1] | 2,33 | B:SER  87 [ HG ] |  | 10 | A:LEU  35 [ O  ] | 2,09 | B:LYS 104 [ HZ1] |
|  |  |  |  |  | 11 | A:GLY  39 [ O  ] | 1,96 | B:LYS 110 [ HZ1] |
| Salt bridges | | | |  |  |  |  |  |
|  | Monomer 1 | Length | Monomer 2 |  | Salt bridges | | | |
| 1 | A:ARG  55 [ NH1] | 3,03 | B:ASP  54 [ OD1] |  |  | Monomer 1 | Length | Monomer 2 |
| 2 | A:ARG  55 [ NH1] | 2,86 | B:ASP  54 [ OD2] |  | 1 | A:LYS 104 [ NZ ] | 2,64 | B:ASP  38 [ OD2] |
| 3 | A:ARG  55 [ NH2] | 3,45 | B:ASP  54 [ OD1] |  | 2 | A:GLU  31 [ OE2] | 3,81 | B:LYS  85 [ NZ ] |
| 4 | A:ARG  55 [ NH2] | 2,96 | B:ASP  54 [ OD2] |  | 3 | A:ASP  38 [ OD2] | 2,99 | B:LYS 104 [ NZ ] |
| 5 | A:LYS  72 [ NZ ] | 3,29 | B:ASP  42 [ OD2] |  |  |  |  |  |
| 6 | A:LYS  73 [ NZ ] | 2,68 | B:GLU  46 [ OE2] |  |  |  |  |  |
| 7 | A:LYS  73 [ NZ ] | 2,66 | B:GLU  46 [ OE1] |  |  |  |  |  |
| 8 | A:ARG 101 [ NH1] | 2,66 | B:GLU  38 [ OE1] |  |  |  |  |  |
| 9 | A:ARG 101 [ NH2] | 3,92 | B:GLU  38 [ OE1] |  |  |  |  |  |
| 10 | A:GLU  36 [ OE1] | 3,64 | B:LYS  75 [ NZ ] |  |  |  |  |  |
| 11 | A:GLU  36 [ OE2] | 2,68 | B:LYS  75 [ NZ ] |  |  |  |  |  |
| 12 | A:GLU  36 [ OE1] | 2,65 | B:LYS 104 [ NZ ] |  |  |  |  |  |

| **AHP2-AHP3** | | | |  | **AHP3-AHP3** | | | |
| --- | --- | --- | --- | --- | --- | --- | --- | --- |
|  |  |  |  |  |  |  |  |  |
| Hydrogen bonds | | | |  | Hydrogen bonds | | | |
|  | Monomer 1 | Length | Monomer 2 |  |  | Monomer 1 | Length | Monomer 2 |
| 1 | A:GLY  39 [ H  ] | 1,82 | B:ASP 108 [ OD2] |  | 1 | A:CYS  39 [ H  ] | 1,80 | B:ASP 108 [ OD1] |
| 2 | A:SER  75 [ HG ] | 1,58 | B:ASP  42 [ OD2] |  | 2 | A:GLN  83 [HE22] | 1,87 | B:ASP  54 [ OD2] |
| 3 | A:GLN  83 [HE22] | 1,82 | B:ASP  54 [ OD2] |  | 3 | A:ASP 108 [ OD1] | 1,82 | B:SER  40 [ H  ] |
| 4 | A:LYS 104 [ HZ3] | 2,07 | B:LEU  35 [ O  ] |  | 4 | A:GLU  46 [ O  ] | 2,02 | B:SER  79 [ HG ] |
| 5 | A:GLU  46 [ O  ] | 1,78 | B:SER  79 [ HG ] |  | 5 | A:ASP  54 [ OD2] | 1,82 | B:GLN  83 [HE21] |
| 6 | A:ASP  54 [ OD2] | 1,71 | B:GLN  83 [HE21] |  |  |  |  |  |
| 7 | A:LEU  35 [ O  ] | 2,45 | B:LYS 104 [ HZ2] |  | Salt bridges | | | |
| 8 | A:GLY  39 [ O  ] | 2,01 | B:GLN 110 [HE21] |  |  | Monomer 1 | Length | Monomer 2 |
|  |  |  |  |  | 1 | A:LYS  57 [ NZ ] | 2,82 | B:ASP  54 [ OD1] |
| Salt bridges | | | |  | 2 | A:LYS  57 [ NZ ] | 2,84 | B:ASP  54 [ OD2] |
|  | Monomer 1 | Length | Monomer 2 |  | 3 | A:LYS  75 [ NZ ] | 3,79 | B:ASP  42 [ OD2] |
| 1 | A:LYS 104 [ NZ ] | 2,66 | B:GLU  38 [ OE1] |  | 4 | A:ASP  54 [ OD1] | 2,76 | B:LYS  57 [ NZ ] |
| 2 | A:ASP  54 [ OD1] | 2,77 | B:LYS  57 [ NZ ] |  | 5 | A:ASP  54 [ OD2] | 2,80 | B:LYS  57 [ NZ ] |
| 3 | A:ASP  54 [ OD2] | 3,11 | B:LYS  57 [ NZ ] |  | 6 | A:ASP  42 [ OD2] | 2,73 | B:LYS  75 [ NZ ] |
| 4 | A:ASP  42 [ OD2] | 3,23 | B:LYS  75 [ NZ ] |  | 7 | A:GLU  46 [ OE2] | 2,66 | B:LYS  75 [ NZ ] |
| 5 | A:ASP  38 [ OD2] | 2,80 | B:LYS 104 [ NZ ] |  | 8 | A:ASP  42 [ OD1] | 3,66 | B:LYS  75 [ NZ ] |
| 6 | A:ASP  38 [ OD1] | 2,75 | B:LYS 104 [ NZ ] |  | 9 | A:GLU  38 [ OE2] | 2,69 | B:LYS 104 [ NZ ] |
|  |  |  |  |  | 10 | A:GLU  38 [ OE1] | 2,70 | B:LYS 104 [ NZ ] |
|  |  |  |  |  |  |  |  |  |
|  |  |  |  |  |  |  |  |  |
| **StHP1a-StHP1a** | | | |  |  |  |  |  |
|  |  |  |  |  |  |  |  |  |
| Hydrogen bonds | | | |  |  |  |  |  |
|  | Monomer 1 | Length | Monomer 2 |  |  |  |  |  |
| 1 | A:SER  37 [ HG ] | 1,75 | B:GLU 249 [ O  ] |  |  |  |  |  |
| 2 | A:ASN  38 [HD21] | 1,84 | B:ASP 214 [ OD1] |  |  |  |  |  |
| 3 | A:GLN  73 [HE22] | 1,72 | B:GLU 188 [ OE1] |  |  |  |  |  |
| 4 | A:GLU 105 [ OE1] | 1,85 | B:SER 181 [ H  ] |  |  |  |  |  |
| 5 | A:GLU 105 [ OE1] | 1,66 | B:SER 181 [ HG ] |  |  |  |  |  |
| 6 | A:CYS 104 [ O  ] | 1,90 | B:ASN 182 [HD22] |  |  |  |  |  |
| 7 | A:GLU  44 [ OE1] | 1,86 | B:GLN 217 [HE21] |  |  |  |  |  |
| 8 | A:ASP  52 [ OD2] | 1,78 | B:GLN 224 [HE21] |  |  |  |  |  |
| 9 | A:LEU  33 [ O  ] | 2,13 | B:ARG 245 [HH22] |  |  |  |  |  |
|  |  |  |  |  |  |  |  |  |
| Salt bridges | | | |  |  |  |  |  |
|  | Monomer 1 | Length | Monomer 2 |  |  |  |  |  |
| 1 | A:LYS  72 [ NZ ] | 3,60 | B:ASP 184 [ OD1] |  |  |  |  |  |
| 2 | A:LYS  72 [ NZ ] | 2,68 | B:ASP 184 [ OD2] |  |  |  |  |  |
| 3 | A:ARG 101 [ NE ] | 2,74 | B:ASP 180 [ OD2] |  |  |  |  |  |
| 4 | A:ARG 101 [ NH2] | 2,80 | B:ASP 180 [ OD2] |  |  |  |  |  |
| 5 | A:ASP  40 [ OD2] | 2,76 | B:LYS 216 [ NZ ] |  |  |  |  |  |
| 6 | A:ASP  36 [ OD1] | 3,74 | B:ARG 245 [ NE ] |  |  |  |  |  |
| 7 | A:ASP  36 [ OD2] | 2,74 | B:ARG 245 [ NH1] |  |  |  |  |  |
| 8 | A:ASP  36 [ OD1] | 3,52 | B:ARG 245 [ NH1] |  |  |  |  |  |
| 9 | A:ASP  36 [ OD2] | 2,86 | B:ARG 245 [ NH2] |  |  |  |  |  |
| 10 | A:ASP  36 [ OD1] | 2,65 | B:ARG 245 [ NH2] |  |  |  |  |  |

**RRrd-HPt complexes**

| **ARR1-AHP2** | | | |  | **ARR2-AHP2** | | | |
| --- | --- | --- | --- | --- | --- | --- | --- | --- |
|  |  |  |  |  |  |  |  |  |
| Hydrogen bonds | | | |  | Hydrogen bonds | | | |
|  | Monomer 1 | Length | Monomer 2 |  |  | Monomer 1 | Length | Monomer 2 |
| 1 | A:ARG  54 [ HE ] | 2,47 | B:SER  40 [ OG ] |  | 1 | A:THR  49 [ HG1] | 1,69 | B:ASP  37 [ OD2] |
| 2 | A:ARG  57 [HH21] | 2,44 | B:GLY  39 [ O  ] |  | 2 | A:ILE 133 [ H  ] | 1,75 | B:GLU  31 [ OE1] |
| 3 | A:MET 142 [ H  ] | 1,78 | B:GLU  31 [ OE1] |  | 3 | A:CYS  39 [ SG ] | 2,07 | B:SER  90 [ HG ] |
| 4 | A:VAL 140 [ O  ] | 1,91 | B:GLN  28 [HE22] |  | 4 | A:VAL 131 [ O  ] | 1,80 | B:GLN  28 [HE22] |
| 5 | A:ASP  44 [ O  ] | 2,21 | B:GLN  83 [HE22] |  |  |  |  |  |
| 6 | A:ASP 118 [ O  ] | 2,11 | B:LYS  85 [ HZ1] |  | Salt bridges | | | |
| 7 | A:ASP 118 [ O  ] | 2,32 | B:LYS  85 [ HZ3] |  |  | Monomer 1 | Length | Monomer 2 |
| 8 | A:THR  47 [ OG1] | 1,75 | B:SER  87 [ HG ] |  | 1 | A:ARG  45 [ NE ] | 3,19 | B:GLU  46 [ OE2] |
| 9 | A:PRO 139 [ O  ] | 1,81 | B:SER  90 [ HG ] |  | 2 | A:ARG  45 [ NH2] | 2,77 | B:GLU  46 [ OE1] |
|  |  |  |  |  | 3 | A:ARG  45 [ NH2] | 2,82 | B:GLU  46 [ OE2] |
| Salt bridges | | | |  | 4 | A:ARG 132 [ NH1] | 3,31 | B:ASP  27 [ OD2] |
|  | Monomer 1 | Length | Monomer 2 |  | 5 | A:ARG 132 [ NH2] | 2,72 | B:ASP  27 [ OD2] |
| 1 | A:ARG  54 [ NE ] | 3,59 | B:GLU  46 [ OE1] |  | 6 | A:ASP 110 [ OD1] | 2,61 | B:LYS  85 [ NZ ] |
| 2 | A:ARG  54 [ NH2] | 2,72 | B:GLU  46 [ OE1] |  | 7 | A:ASP 110 [ OD2] | 2,85 | B:LYS  85 [ NZ ] |
| 3 | A:ARG  54 [ NH2] | 2,87 | B:GLU  46 [ OE2] |  | 8 | A:ASP 110 [ OD2] | 2,71 | B:LYS 104 [ NZ ] |
| 4 | A:ARG  57 [ NH1] | 3,29 | B:ASP  37 [ OD1] |  |  |  |  |  |
| 5 | A:ARG  57 [ NH1] | 2,74 | B:ASP  37 [ OD2] |  |  |  |  |  |
| 6 | A:ARG  57 [ NH2] | 3,47 | B:ASP  37 [ OD1] |  |  |  |  |  |
| 7 | A:ARG  57 [ NH2] | 2,85 | B:ASP  37 [ OD2] |  |  |  |  |  |
| 8 | A:ASP 119 [ OD2] | 2,69 | B:LYS  85 [ NZ ] |  |  |  |  |  |
| 9 | A:ASP 119 [ OD1] | 2,79 | B:LYS  97 [ NZ ] |  |  |  |  |  |
| 10 | A:ASP 119 [ OD2] | 2,75 | B:LYS  97 [ NZ ] |  |  |  |  |  |
|  |  |  |  |  |  |  |  |  |
|  |  |  |  |  |  |  |  |  |
| **ARR10-AHP2** | | | |  | **ARR11-AHP2** | | | |
|  |  |  |  |  |  |  |  |  |
| Hydrogen bonds | | | |  | Hydrogen bonds | | | |
|  | Monomer 1 | Length | Monomer 2 |  |  | Monomer 1 | Length | Monomer 2 |
| 1 | A:ILE 122 [ H  ] | 1,81 | B:GLU  31 [ OE1] |  | 1 | A:LYS  24 [ HZ3] | 1,79 | B:GLU  46 [ O  ] |
| 2 | A:VAL 120 [ O  ] | 1,91 | B:GLN  28 [HE22] |  | 2 | A:MET 116 [ H  ] | 1,87 | B:GLU  31 [ OE1] |
| 3 | A:HIS  98 [ O  ] | 1,85 | B:LYS  85 [ HZ1] |  | 3 | A:ASP  19 [ OD2] | 1,67 | B:SER  87 [ HG ] |
| 4 | A:SER  99 [ OG ] | 1,85 | B:LYS  85 [ HZ2] |  | 4 | A:ASP  92 [ O  ] | 1,74 | B:LYS  85 [ HZ3] |
| 5 | A:ASP  25 [ OD1] | 1,64 | B:SER  87 [ HG ] |  | 5 | A:ILE 114 [ O  ] | 1,90 | B:GLN  28 [HE22] |
| 6 | A:CYS  28 [ SG ] | 2,06 | B:SER  90 [ HG ] |  |  |  |  |  |
|  |  |  |  |  | Salt bridges | | | |
| Salt bridges | | | |  |  | Monomer 1 | Length | Monomer 2 |
|  | Monomer 1 | Length | Monomer 2 |  | 1 | A:LYS  28 [ NZ ] | 3,34 | B:GLU  46 [ OE1] |
| 1 | A:ARG  30 [ NH1] | 2,71 | B:GLU  46 [ OE1] |  | 2 | A:LYS  28 [ NZ ] | 2,72 | B:GLU  46 [ OE2] |
| 2 | A:ARG  30 [ NH1] | 3,49 | B:GLU  46 [ OE2] |  | 3 | A:LYS  32 [ NZ ] | 3,10 | B:ASP  37 [ OD1] |
| 3 | A:ARG  30 [ NH2] | 3,26 | B:GLU  46 [ OE1] |  | 4 | A:LYS  32 [ NZ ] | 2,67 | B:ASP  37 [ OD2] |
| 4 | A:ARG  30 [ NH2] | 2,76 | B:GLU  46 [ OE2] |  | 5 | A:ARG 115 [ NH1] | 2,72 | B:ASP  27 [ OD2] |
| 5 | A:ARG 121 [ NE ] | 2,81 | B:GLU  31 [ OE2] |  | 6 | A:LYS 117 [ NZ ] | 2,68 | B:GLU  31 [ OE2] |
| 6 | A:ARG 121 [ NH2] | 2,72 | B:GLU  31 [ OE2] |  | 7 | A:ASP  92 [ OD1] | 3,43 | B:LYS  85 [ NZ ] |
| 7 | A:ASP 100 [ OD2] | 3,66 | B:HIS  82 [ ND1] |  | 8 | A:ASP  92 [ OD2] | 2,73 | B:LYS  85 [ NZ ] |
| 8 | A:ASP  71 [ OD2] | 2,81 | B:HIS  82 [ NE2] |  | 9 | A:GLU  94 [ OE1] | 3,72 | B:HIS  82 [ NE2] |
| 9 | A:ASP 100 [ OD1] | 2,67 | B:LYS 104 [ NZ ] |  | 10 | A:GLU  94 [ OE2] | 2,82 | B:HIS  82 [ NE2] |
| 10 | A:ASP 100 [ OD2] | 2,83 | B:LYS 104 [ NZ ] |  |  |  |  |  |
|  |  |  |  |  |  |  |  |  |
|  |  |  |  |  |  |  |  |  |
| **StRR1a-StHP1a** | | | |  | **StRR11-StHP1a** | | | |
|  |  |  |  |  |  |  |  |  |
| Hydrogen bonds | | | |  | Hydrogen bonds | | | |
|  | Monomer 1 | Length | Monomer 2 |  |  | Monomer 1 | Length | Monomer 2 |
| 1 | A:LYS  46 [ HZ2] | 1,92 | B:ASN  38 [ OD1] |  | 1 | A:LYS  40 [ HZ3] | 2,20 | B:SER  37 [ O  ] |
| 2 | A:ARG 133 [HH22] | 2,07 | B:GLN  29 [ OE1] |  | 2 | A:ARG 127 [ HE ] | 1,85 | B:GLN  26 [ OE1] |
| 3 | A:ASP  37 [ OD2] | 1,70 | B:SER  84 [ HG ] |  | 3 | A:ARG 127 [HH21] | 2,11 | B:GLN  26 [ OE1] |
| 4 | A:THR  39 [ OG1] | 1,73 | B:SER  87 [ HG ] |  | 4 | A:MET 128 [ H  ] | 2,03 | B:GLN  29 [ OE1] |
| 5 | A:ASP 110 [ O  ] | 1,73 | B:LYS  82 [ HZ3] |  | 5 | A:ASP  31 [ OD2] | 1,75 | B:SER  84 [ HG ] |
| 6 | A:ASP 111 [ O  ] | 2,48 | B:LYS  82 [ HZ1] |  | 6 | A:ASP 104 [ O  ] | 2,43 | B:LYS  82 [ HZ1] |
| 7 | A:VAL 132 [ O  ] | 1,90 | B:GLN  26 [HE22] |  | 7 | A:ASP 104 [ O  ] | 2,09 | B:LYS  82 [ HZ3] |
|  |  |  |  |  | 8 | A:ILE 126 [ O  ] | 1,88 | B:GLN  26 [HE22] |
| Salt bridges | | | |  |  |  |  |  |
|  | Monomer 1 | Length | Monomer 2 |  | Salt bridges | | | |
| 1 | A:LYS  42 [ NZ ] | 2,90 | B:GLU  44 [ OE1] |  |  | Monomer 1 | Length | Monomer 2 |
| 2 | A:LYS  42 [ NZ ] | 2,70 | B:GLU  44 [ OE2] |  | 1 | A:LYS  36 [ NZ ] | 2,81 | B:GLU  44 [ OE1] |
|  |  |  |  |  | 2 | A:LYS  36 [ NZ ] | 2,76 | B:GLU  44 [ OE2] |
|  |  |  |  |  | 3 | A:LYS  40 [ NZ ] | 2,66 | B:ASP  35 [ OD1] |
|  |  |  |  |  | 4 | A:ARG 127 [ NH2] | 2,76 | B:ASP  24 [ OD1] |
|  |  |  |  |  | 5 | A:ASP 104 [ OD1] | 2,73 | B:LYS  82 [ NZ ] |
|  |  |  |  |  | 6 | A:ASP 104 [ OD2] | 2,70 | B:LYS  94 [ NZ ] |
