# Supplementary material for: Modeling of Protein–Protein Interactions in Cytokinin Signal Transduction
Source: Int J Mol Sci. 2019 Apr 28;20(9):2096. doi: 10.3390/ijms20092096 (PMC6539988; doi:10.3390/ijms20092096)
Supplement: Supplementary file 1 [file ijms-20-02096-s001.zip › Supplementary_tables_and_figures.docx]

Supplementary data

to the paper " Modeling of protein-protein interactions in cytokinin signal transduction" by Dmitry V. Arkhipov, Sergey N. Lomin, Yulia A. Myakushina, Ekaterina M. Savelieva, Dmitry I. Osolodkin, and Georgy A. Romanov

**Table S1**. Sequence identity of modeled proteins and respective templates.

| Domain / Protein family | Template | | Modelled protein | Identity, % | Reference |
| --- | --- | --- | --- | --- | --- |
|  | PDB ID | Protein |  |  |  |
| Sensory module | 3T4L_A | AHK4 | AHK2 | 61% | Hothorn et al., 2011 [4] |
|  |  |  | AHK3 | 61% |  |
|  |  |  | AHK4 | 99% |  |
|  |  |  | StHK2 | 65% |  |
|  |  |  | StHK3 | 65% |  |
|  |  |  | StHK4 | 80% |  |
|  | | | | | |
| HisKA domain | 4MT8_A | ERS1 | AHK2 | 37% | Mayerhofer et al., 2015 [6] |
|  |  |  | AHK3 | 36% |  |
|  |  |  | AHK4 | 41% |  |
|  |  |  | StHK2 | 37% |  |
|  |  |  | StHK3 | 35% |  |
|  |  |  | StHK4 | 38% |  |
|  | | | | | |
| Histidine kinase Receiver domain | 3MMN_A | CKI1 | AHK2 | 36% | Pekárová et al., 2011 [17] |
|  |  |  | AHK3 | 33% |  |
|  |  |  | AHK4 | 41% |  |
|  |  |  | StHK2 | 48% |  |
|  |  |  | StHK3 | 44% |  |
|  |  |  | StHK4 | 49% |  |
|  |  | | | | |
|  | 4EUK_A | AHK5/CKI2 | AHK2 | 32% | Bauer et al., 2013 [19] |
|  |  |  | AHK3 | 35% |  |
|  |  |  | AHK4 | 36% |  |
|  |  |  | StHK2 | 33% |  |
|  |  |  | StHK3 | 32% |  |
|  |  |  | StHK4 | 32% |  |
|  | | | | | |
| Histidine containing phosphotransfer protein | 4EUK_B | AHP1 | AHP1 | 100% | Bauer et al., 2013 [19] |
|  |  |  | AHP2 | 49% |  |
|  |  |  | AHP3 | 53% |  |
|  |  |  | StHP1a | 70% |  |
|  |  | | | | |
|  | 1YVI | OsHP1 | AHP1 | 51% | Bae et al., 2010; Levin et al., 2007 [23,24] |
|  |  |  | AHP2 | 43% |  |
|  |  |  | AHP3 | 44% |  |
|  |  |  | StHP1a | 49% |  |
|  | | | | | |
| Response regulator receiver domain complex with phosphotransmitter | 1CHN | CheY | ARR1 | 34% | Bellsolell et al., 1994 [39] |
|  |  |  | ARR2 | 32% |  |
|  |  |  | ARR10 | 28% |  |
|  |  |  | ARR11 | 33% |  |
|  |  |  | StRR1a | 32% |  |
|  |  |  | StRR11 | 32% |  |
|  |  | | | | |
|  | 4EUK_A | AHK5/CKI2 | ARR1 | 20% | Bauer et al., 2013 [19] |
|  |  |  | ARR2 | 20% |  |
|  |  |  | ARR10 | 20% |  |
|  |  |  | ARR11 | 19% |  |
|  |  |  | StRR1a | 20% |  |
|  |  |  | StRR11 | 19% |  |

**Table S2**. Main Ramachandran plot parameters of modeled structures upon minimization procedures.

| complex type | Modelled complex | Main Ramachandran plot parameters | | | |
| --- | --- | --- | --- | --- | --- |
|  |  | Most favored regions | Additional allowed regions | Generously allowed regions | Disallowed regions |
| Sensor module dimer | AHK2sm-AHK2sm | 88.1% | 11.1% | 0.4% | 0.4% |
|  | AHK2sm-AHK3sm | 87.3% | 11.9% | 0.2% | 0.6% |
|  | AHK2sm-AHK4sm | 89.3% | 9.9% | 0.6% | 0.2% |
|  | AHK3sm-AHK3sm | 87.5% | 10.2% | 1.6% | 0.8% |
|  | AHK3sm-AHK4sm | 87.7% | 9.9% | 1.6% | 0.8% |
|  | AHK4sm-AHK4sm | 89.3% | 9.9% | 0.4% | 0.4% |
|  | StHK2sm-StHK2sm | 87.3% | 11.3% | 0.8% | 0.6% |
|  | StHK3sm-StHK3sm | 88.5% | 10.1% | 0.6% | 0.8% |
|  | StHK4sm-StHK4sm | 89.0% | 10.4% | 0.6% | 0.0% |
|  | | | | | |
| HisKA domain dimer | AHK2hk-AHK2hk | 96.8% | 3.2% | 0.0% | 0.0% |
|  | AHK2hk-AHK3hk | 96.9% | 3.1% | 0.0% | 0.0% |
|  | AHK2hk-AHK4hk | 95.3% | 4.7% | 0.0% | 0.0% |
|  | AHK3hk-AHK3hk | 96.4% | 3.6% | 0.0% | 0.0% |
|  | AHK3hk-AHK4hk | 96.9% | 3.1% | 0.0% | 0.0% |
|  | AHK4hk-AHK4hk | 96.8% | 3.2% | 0.0% | 0.0% |
|  | StHK2hk-StHK2hk | 96.3% | 3.7% | 0.0% | 0.0% |
|  | StHK3hk-StHK3hk | 96.3% | 3.7% | 0.0% | 0.0% |
|  | StHK4hk-StHK4hk | 97.3% | 2.7% | 0.0% | 0.0% |
|  | | | | | |
| Receptor receiver domain complex with phosphotransmitter | AHK2rd-AHP1 | 90.4% | 9.2% | 0.0% | 0.4% |
|  | AHK2rd-AHP2 | 89.0% | 10.3% | 0.4% | 0.4% |
|  | AHK2rd-AHP3 | 88.9% | 9.2% | 1.1% | 0.7% |
|  | AHK3rd-AHP1 | 89.8% | 9.1% | 0.7% | 0.4% |
|  | AHK3rd-AHP2 | 90.9% | 8.0% | 0.4% | 0.0% |
|  | AHK3rd-AHP3 | 89.0% | 10.3% | 0.7% | 0.0% |
|  | AHK4rd-AHP1 | 90.2% | 9.8% | 0.0% | 0.0% |
|  | AHK4rd-AHP2 | 87.9% | 11.0% | 0.4% | 0.8% |
|  | AHK4rd-AHP3 | 88.2% | 11.4% | 0.4% | 0.0% |
|  | AHK5rd-AHP1 | 89.5% | 10.1% | 0.0% | 0.4% |
|  | StHK2rd-StHP1a | 90.2% | 9.1% | 0.8% | 0.0% |
|  | StHK3rd-StHP1a | 90.6% | 8.6% | 0.0% | 0.8% |
|  | StHK4rd-StHP1a | 89.1% | 9.8% | 0.0% | 1.1% |
|  | | | | | |
| Рhosphotransmitter dimer | AHP1-AHP1 | 95.2% | 4.8% | 0.0% | 0.0% |
|  | AHP1-AHP2 | 94.1% | 5.6% | 0.0% | 0.4% |
|  | AHP1-AHP3 | 93.0% | 6.6% | 0.0% | 0.4% |
|  | AHP2-AHP2 | 94.1% | 5.9% | 0.0% | 0.0% |
|  | AHP2-AHP3 | 94.5% | 5.5% | 0.0% | 0.0% |
|  | AHP3-AHP3 | 88.2% | 11.8% | 0.0% | 0.0% |
|  | StHP1a-StHP1a | 91.5% | 8.5% | 0.0% | 0.0% |
|  | | | | | |
| Response regulator receiver domain complex with phosphotransmitter | ARR1rd-AHP2 | 88.2% | 9.8% | 0.8% | 1.2% |
|  | ARR2rd-AHP2 | 88.6% | 10.6% | 0.0% | 0.8% |
|  | ARR10rd-AHP2 | 87.1% | 11.8% | 0.4% | 0.8% |
|  | ARR11rd-AHP2 | 91.7% | 7.5% | 0.4% | 0.4% |
|  | StRR1a_rd-StHP1a | 92.4% | 7.2% | 0.0% | 0.4% |
|  | StRR11rd-StHP1a | 90.8% | 8.8% | 0.0% | 0.4% |

**Table S3**. PISA Interface parameters of modeled structures.

| complex type | Modeled complex | PISA Interface parameters | | | | | | | | |
| --- | --- | --- | --- | --- | --- | --- | --- | --- | --- | --- |
|  |  | Interface area, Å² | Solvation Energy, kcal/mol | Solvation Energy, kJ/mol | Total Binding Energy, kcal/mol | Total Binding Energy, kJ/mol | Hydrophobic P-value | Number of Hydrogen Bonds | Number of Salt Bridges | Number of Disulphide Bonds |
| Sensor module dimer | AHK2sm-AHK2sm | 997 | -7.82 | -32.71 | -13.00 | -54.39 | 0.52 | 10 | 2 | 0 |
|  | AHK2sm-AHK3sm | 1013 | -9.21 | -38.53 | -14.76 | -61.76 | 0.38 | 10 | 3 | 0 |
|  | AHK2sm-AHK4sm | 999 | -10.14 | -42.43 | -14.88 | -62.26 | 0.34 | 9 | 2 | 0 |
|  | AHK3sm-AHK3sm | 1044 | -10.02 | -41.92 | -16.76 | -70.12 | 0.36 | 11 | 5 | 0 |
|  | AHK3sm-AHK4sm | 986 | -9.70 | -40.59 | -14.00 | -58.58 | 0.35 | 8 | 2 | 0 |
|  | AHK4sm-AHK4sm | 1009 | -8.57 | -35.84 | -13.98 | -58.49 | 0.42 | 8 | 5 | 0 |
|  | StHK2sm-StHK2sm | 1005 | -12.65 | -52.93 | -17.09 | -71.50 | 0.25 | 10 | 0 | 0 |
|  | StHK3sm-StHK3sm | 962 | -11.64 | -48.70 | -16.82 | -70.37 | 0.20 | 10 | 2 | 0 |
|  | StHK4sm-StHK4sm | 947 | -11.26 | -47.11 | -13.86 | -57.99 | 0.25 | 5 | 1 | 0 |
|  | | | | | | | | | | |
| HisKA domain dimer | AHK2hk-AHK2hk | 2127 | -51.92 | -217.23 | -57.04 | -238.66 | 0.09 | 4 | 9 | 0 |
|  | AHK2hk-AHK3hk | 2080 | -50.09 | -209.58 | -56.25 | -235.35 | 0.09 | 8 | 7 | 0 |
|  | AHK2hk-AHK4hk | 2242 | -52.75 | -220.71 | -60.32 | -252.38 | 0.19 | 7 | 12 | 0 |
|  | AHK3hk-AHK3hk | 2267 | -52.38 | -219.16 | -57.72 | -241.50 | 0.20 | 7 | 6 | 0 |
|  | AHK3hk-AHK4hk | 1977 | -52.28 | -218.74 | -56.29 | -235.52 | 0.06 | 4 | 6 | 0 |
|  | AHK4hk-AHK4hk | 2157 | -56.72 | -237.32 | -61.31 | -256.52 | 0.14 | 7 | 4 | 0 |
|  | StHK2hk-StHK2hk | 2033 | -47.71 | -199.62 | -53.35 | -223.22 | 0.10 | 6 | 8 | 0 |
|  | StHK3hk-StHK3hk | 2175 | -48.36 | -202.34 | -56.68 | -237.15 | 0.25 | 7 | 14 | 0 |
|  | StHK4hk-StHK4hk | 2097 | -50.31 | -210.50 | -56.25 | -235.35 | 0.07 | 5 | 10 | 0 |
|  | | | | | | | | | | |
| Receptor receiver domain complex with phosphotransmitter | AHK2rd-AHP1 | 828 | -3.16 | -13.20 | -9.67 | -40.47 | 0.80 | 13 | 2 | 0 |
|  | AHK2rd-AHP2 | 828 | -3.52 | -14.71 | -9.67 | -40.47 | 0.75 | 8 | 7 | 0 |
|  | AHK2rd-AHP3 | 826 | -3.69 | -15.43 | -11.03 | -46.15 | 0.78 | 9 | 9 | 0 |
|  | AHK3rd-AHP1 | 778 | -8.56 | -35.82 | -12.49 | -52.26 | 0.27 | 8 | 1 | 0 |
|  | AHK3rd-AHP2 | 818 | -3.44 | -14.38 | -9.67 | -40.45 | 0.59 | 9 | 6 | 0 |
|  | AHK3rd-AHP3 | 815 | -2.49 | -10.42 | -9.09 | -38.04 | 0.70 | 9 | 7 | 0 |
|  | AHK4rd-AHP1 | 791 | -5.22 | -21.84 | -10.48 | -43.85 | 0.71 | 11 | 1 | 0 |
|  | AHK4rd-AHP2 | 845 | -7.04 | -29.44 | -11.49 | -48.07 | 0.56 | 5 | 6 | 0 |
|  | AHK4rd-AHP3 | 873 | -3.42 | -14.31 | -10.39 | -43.47 | 0.72 | 9 | 8 | 0 |
|  | AHK5rd-AHP1 | 821 | -9.85 | -41.22 | -14.22 | -59.50 | 0.16 | 9 | 1 | 0 |
|  | StHK2rd-StHP1a | 942 | -3.63 | -15.19 | -11.12 | -46.53 | 0.75 | 11 | 7 | 0 |
|  | StHK3rd-StHP1a | 815 | -4.89 | -20.48 | -11.05 | -46.23 | 0.57 | 8 | 7 | 0 |
|  | StHK4rd-StHP1a | 907 | -2.15 | -9.01 | -8.61 | -36.03 | 0.81 | 7 | 9 | 0 |
|  | | | | | | | | | | |
| Рhosphotransmitter dimer | AHP1-AHP1 | 1253 | -5.12 | -21.44 | -12.24 | -51.21 | 0.68 | 11 | 6 | 0 |
|  | AHP1-AHP2 | 1127 | -7.28 | -30.44 | -13.73 | -57.45 | 0.65 | 7 | 9 | 0 |
|  | AHP1-AHP3 | 1259 | -7.05 | -29.48 | -15.95 | -66.73 | 0.58 | 10 | 12 | 0 |
|  | AHP2-AHP2 | 1151 | -9.59 | -40.12 | -15.59 | -65.23 | 0.45 | 11 | 3 | 0 |
|  | AHP2-AHP3 | 1117 | -11.37 | -47.57 | -17.16 | -71.80 | 0.39 | 8 | 6 | 0 |
|  | AHP3-AHP3 | 1169 | -11.50 | -48.12 | -17.44 | -72.97 | 0.38 | 5 | 10 | 0 |
|  | StHP1a-StHP1a | 1215 | -5.91 | -24.72 | -13.62 | -56.99 | 0.61 | 9 | 10 | 0 |
|  | | | | | | | | | | |
| Response regulator receiver domain complex with phosphotransmitter | ARR1-AHP2 | 969 | -11.21 | -46.90 | -18.93 | -79.20 | 0.24 | 9 | 10 | 0 |
|  | ARR2-AHP2 | 949 | -11.35 | -47.49 | -16.10 | -67.36 | 0.28 | 4 | 8 | 0 |
|  | ARR10-AHP2 | 930 | -6.49 | -27.14 | -12.87 | -53.85 | 0.48 | 6 | 10 | 0 |
|  | ARR11-AHP2 | 1013 | -7.82 | -32.72 | -13.76 | -57.57 | 0.47 | 5 | 10 | 0 |
|  | StRR1a-StHP1a | 881 | -9.45 | -39.54 | -13.30 | -55.65 | 0.24 | 7 | 2 | 0 |
|  | StRR11-StHP1a | 930 | -5.98 | -25.02 | -11.76 | -49.20 | 0.40 | 8 | 6 | 0 |

| complex type | Modelled complex | PRODIGY Interface parameters | | | | | | | | | | | |
| --- | --- | --- | --- | --- | --- | --- | --- | --- | --- | --- | --- | --- | --- |
|  |  | No. of intermolecular contacts | No. of charged-charged contacts | No. of charged-polar contacts | No. of charged-apolar contacts | No. of polar-polar contacts | No. of apolar-polar contacts | No. of apolar-apolar contacts | Percentage of apolar NIS residues | Percentage of charged NIS residues | Predicted binding affinity (kcal/mol) | Predicted binding affinity (kJ/mol) | Predicted dissociation constant (M) |
| Sensor module dimer | AHK2sm-AHK2sm | 81 | 8 | 16 | 19 | 2 | 12 | 24 | 37 | 31 | -9.8 | -41.00 | 6.8E-08 |
|  | AHK2sm-AHK3sm | 88 | 11 | 18 | 19 | 2 | 13 | 25 | 37 | 30 | -10.3 | -43.10 | 2.8E-08 |
|  | AHK2sm-AHK4sm | 82 | 12 | 16 | 15 | 2 | 11 | 26 | 39 | 31 | -9.1 | -38.07 | 2.0E-07 |
|  | AHK3sm-AHK3sm | 91 | 12 | 20 | 19 | 2 | 13 | 25 | 38 | 30 | -10.3 | -43.10 | 2.9E-08 |
|  | AHK3sm-AHK4sm | 82 | 11 | 15 | 17 | 2 | 13 | 24 | 39 | 31 | -9.7 | -40.58 | 7.2E-08 |
|  | AHK4sm-AHK4sm | 79 | 14 | 12 | 14 | 2 | 11 | 26 | 40 | 31 | -9 | -37.66 | 2.5E-07 |
|  | StHK2sm-StHK2sm | 89 | 9 | 17 | 22 | 2 | 13 | 26 | 39 | 29 | -10.3 | -43.10 | 2.9E-08 |
|  | StHK3sm-StHK3sm | 76 | 5 | 14 | 14 | 4 | 14 | 25 | 38 | 30 | -9 | -37.66 | 2.7E-07 |
|  | StHK4sm-StHK4sm | 73 | 10 | 13 | 13 | 1 | 10 | 26 | 40 | 31 | -8.6 | -35.98 | 4.7E-07 |
|  | | | | | | | | | | | | | |
| HisKA domain dimer | AHK2hk-AHK2hk | 138 | 8 | 6 | 26 | 3 | 17 | 78 | 42 | 30 | -10.5 | -43.93 | 1.9E-08 |
|  | AHK2hk-AHK3hk | 136 | 10 | 5 | 25 | 4 | 23 | 69 | 42 | 29 | -12 | -50.21 | 1.7E-09 |
|  | AHK2hk-AHK4hk | 146 | 14 | 5 | 33 | 3 | 14 | 77 | 45 | 28 | -10.8 | -45.19 | 1.2E-08 |
|  | AHK3hk-AHK3hk | 151 | 11 | 6 | 30 | 3 | 27 | 74 | 41 | 30 | -13.7 | -57.32 | 8.5E-11 |
|  | AHK3hk-AHK4hk | 133 | 9 | 4 | 27 | 3 | 14 | 76 | 45 | 27 | -9.8 | -41.00 | 6.0E-08 |
|  | AHK4hk-AHK4hk | 148 | 10 | 3 | 34 | 2 | 11 | 88 | 49 | 26 | -9.6 | -40.17 | 8.7E-08 |
|  | StHK2hk-StHK2hk | 128 | 8 | 5 | 25 | 6 | 19 | 65 | 45 | 23 | -10.7 | -44.77 | 1.4E-08 |
|  | StHK3hk-StHK3hk | 146 | 11 | 3 | 26 | 5 | 24 | 77 | 42 | 25 | -12.7 | -53.14 | 4.6E-10 |
|  | StHK4hk-StHK4hk | 137 | 12 | 3 | 29 | 3 | 18 | 72 | 38 | 29 | -12.4 | -51.88 | 8.4E-10 |
|  | | | | | | | | | | | | | |
| Receptor receiver domain complex with phosphotransmitter | AHK2rd-AHP1 | 63 | 2 | 16 | 14 | 4 | 14 | 13 | 36 | 33 | -8.8 | -36.82 | 3.6E-07 |
|  | AHK2rd-AHP2 | 62 | 9 | 9 | 15 | 4 | 12 | 13 | 37 | 34 | -8.7 | -36.40 | 3.9E-07 |
|  | AHK2rd-AHP3 | 65 | 10 | 7 | 18 | 5 | 11 | 14 | 37 | 34 | -8.6 | -35.98 | 4.8E-07 |
|  | AHK3rd-AHP1 | 60 | 4 | 11 | 12 | 5 | 15 | 13 | 35 | 32 | -8.9 | -37.24 | 3.1E-07 |
|  | AHK3rd-AHP2 | 65 | 7 | 9 | 17 | 8 | 12 | 12 | 36 | 35 | -8 | -33.47 | 1.3E-06 |
|  | AHK3rd-AHP3 | 62 | 8 | 11 | 14 | 5 | 12 | 12 | 35 | 34 | -8.6 | -35.98 | 5.2E-07 |
|  | AHK4rd-AHP1 | 60 | 2 | 14 | 11 | 4 | 14 | 15 | 36 | 31 | -8.6 | -35.98 | 4.7E-07 |
|  | AHK4rd-AHP2 | 63 | 10 | 7 | 14 | 5 | 13 | 14 | 39 | 31 | -8.7 | -36.40 | 4.0E-07 |
|  | AHK4rd-AHP3 | 72 | 13 | 9 | 19 | 5 | 13 | 13 | 37 | 31 | -9.8 | -41.00 | 6.1E-08 |
|  | AHK5rd-AHP1 | 65 | 4 | 10 | 5 | 9 | 22 | 15 | 33 | 28 | -10 | -41.84 | 4.3E-08 |
|  | StHK2rd-StHP1a | 69 | 4 | 15 | 17 | 5 | 15 | 13 | 36 | 30 | -9.7 | -40.58 | 7.8E-08 |
|  | StHK3rd-StHP1a | 58 | 5 | 9 | 11 | 7 | 14 | 12 | 39 | 29 | -8.2 | -34.31 | 9.6E-07 |
|  | StHK4rd-StHP1a | 67 | 8 | 17 | 11 | 5 | 14 | 12 | 37 | 32 | -8.7 | -36.40 | 4.2E-07 |
|  | | | | | | | | | | | | | |
| Рhosphotransmitter dimer | AHP1-AHP1 | 98 | 14 | 24 | 31 | 8 | 13 | 8 | 31 | 29 | -12 | -50.21 | 1.5E-09 |
|  | AHP1-AHP2 | 87 | 14 | 17 | 23 | 8 | 16 | 9 | 31 | 31 | -11.6 | -48.53 | 3.0E-09 |
|  | AHP1-AHP3 | 100 | 17 | 15 | 36 | 6 | 20 | 6 | 31 | 31 | -14.4 | -60.25 | 2.7E-11 |
|  | AHP2-AHP2 | 88 | 11 | 17 | 23 | 5 | 18 | 14 | 34 | 32 | -11.6 | -48.53 | 2.9E-09 |
|  | AHP2-AHP3 | 88 | 14 | 15 | 23 | 8 | 17 | 11 | 33 | 32 | -11.2 | -46.86 | 5.7E-09 |
|  | AHP3-AHP3 | 83 | 13 | 13 | 23 | 4 | 23 | 7 | 34 | 31 | -13.3 | -55.65 | 1.8E-10 |
|  | StHP1a-StHP1a | 91 | 18 | 18 | 27 | 7 | 14 | 7 | 32 | 29 | -12.1 | -50.63 | 1.4E-09 |
|  | | | | | | | | | | | | | |
| Response regulator receiver domain complex with phosphotransmitter | ARR1rd-AHP2 | 72 | 12 | 15 | 14 | 2 | 16 | 13 | 39 | 32 | -10.1 | -42.26 | 4.2E-08 |
|  | ARR2rd-AHP2 | 69 | 9 | 12 | 16 | 2 | 17 | 13 | 39 | 32 | -10.2 | -42.68 | 3.2E-08 |
|  | ARR10rd-AHP2 | 67 | 10 | 13 | 11 | 4 | 19 | 10 | 37 | 31 | -10.3 | -43.10 | 2.7E-08 |
|  | ARR11rd-AHP2 | 67 | 11 | 12 | 18 | 2 | 15 | 9 | 39 | 34 | -9.7 | -40.58 | 7.4E-08 |
|  | StRR1a_rd-StHP1a | 69 | 8 | 15 | 12 | 3 | 20 | 11 | 38 | 31 | -10.5 | -43.93 | 1.9E-08 |
|  | StRR11rd-StHP1a | 75 | 10 | 16 | 15 | 2 | 21 | 11 | 37 | 33 | -11.4 | -47.70 | 4.4E-09 |

**Table S4**. PRODIGY Interface parameters of the models.

|  | AHK2sm-AHK2sm | | AHK2sm-AHK3sm | | AHK2sm-AHK4sm | | AHK3sm-AHK3sm | | AHK3sm-AHK4sm | | AHK4sm-AHK4sm | | StHK2sm-StHK2sm | | StHK3sm-StHK3sm | | StHK4sm-StHK4sm | |
| --- | --- | --- | --- | --- | --- | --- | --- | --- | --- | --- | --- | --- | --- | --- | --- | --- | --- | --- |
|  | mutated residue | ΔΔG of complex (kJ/mol) | mutated residue | ΔΔG of complex (kJ/mol) | mutated residue | ΔΔG of complex (kJ/mol) | mutated residue | ΔΔG of complex (kJ/mol) | mutated residue | ΔΔG of complex (kJ/mol) | mutated residue | ΔΔG of complex (kJ/mol) | mutated residue | ΔΔG of complex (kJ/mol) | mutated residue | ΔΔG of complex (kJ/mol) | mutated residue | ΔΔG of complex (kJ/mol) |
| CHAIN A | V 282 | 0.17 | V 282 | 0.31 | V 282 | 0.17 | V 143 | 0.27 | V 143 | 0.17 | V 178 | 0.17 | V 360 | 0.26 | V 129 | 0.30 | V 85 | 0.21 |
|  | N 285 | 0.11 | N 285 | 0.06 | N 285 | 0 | N 146 | 1.93 | N 146 | 0.07 | N 181 | 1.87 | N 363 | 1.74 | N 132 | 0.02 | N 88 | 0.05 |
|  | H 286 | 0.9 | H 286 | 2.16 | H 286 | 1.89 | H 147 | 2 | H 147 | 2.94 | H 182 | 2.81 | H 364 | 1.08 | H 133 | 3.45 | H 89 | 1.88 |
|  | H 288 | 0.5 | H 288 | 0.49 | H 288 | 0.52 | Q 149 | 0.35 | Q 149 | 0.34 | H 184 | 0.51 | H 366 | 0.53 | Q 135 | 0.13 | H 91 | 0.49 |
|  | I 292 | 1.9 | I 292 | 1.9 | I 292 | 1.84 | I 153 | 1.87 | I 153 | 1.69 | I 188 | 1.86 | I 370 | 1.92 | I 139 | 1.89 | I 95 | 1.89 |
|  | L 293 | 1.22 | L 293 | 1.06 | L 293 | 1.24 | L 154 | 1.21 | L 154 | 1.01 | L 189 | 1.15 | L 371 | 1.11 | L 140 | 1.16 | L 96 | 1.14 |
|  | S 295 | 0.64 | S 295 | 0.81 | S 295 | 1.11 | S 156 | 1.15 | S 156 | 0.51 | S 191 | 0.63 | S 373 | 0.70 | S 142 | 0.64 | S 98 | 0.64 |
|  | T 296 | 0.44 | T 296 | 0.53 | T 296 | 0.48 | T 157 | 0.91 | T 157 | 0.69 | T 192 | 0.44 | T 374 | 0.49 | T 143 | 1.52 | T 99 | 0.30 |
|  | F 297 | 4.06 | F 297 | 3.73 | F 297 | 3.9 | F 158 | 4 | F 158 | 3.82 | F 193 | 4.05 | F 375 | 4.22 | F 144 | 3.91 | F 100 | 3.64 |
|  |  |  |  |  |  |  |  |  |  |  | H 196 | 0.52 |  |  |  |  |  |  |
|  | K 301 | 1.12 | K 301 | 2.33 | K 301 | 2.25 | K 162 | 0.75 | K 162 | 0.39 | K 197 | 1.61 | K 379 | 0.37 | R 148 | 1.78 | K 104 | 0.25 |
|  |  |  | I 306 | 0.25 | I 306 | 0.2 | I 167 | 0.29 | I 167 | 0.28 | I 202 | 0.3 |  |  |  |  | I 109 | 0.25 |
|  | D 307 | 0.29 | D 307 | 0.43 | D 307 | 0.11 | D 168 | 0.41 |  |  | D 203 | 0.98 |  |  |  |  | D 110 | -0.10 |
|  |  |  | R 309 | -0.13 |  |  | R 170 | -0.03 |  |  | E 205 | 0.43 |  |  | C 156 | -0.07 |  |  |
|  | T 310 | 2 | T 310 | 1.93 | T 310 | 1.58 | T 171 | 2.02 | T 171 | 2.07 | T 206 | 0.51 | T 388 | 2.43 | T 157 | 2.25 | T 113 | 2.26 |
|  | E 313 | -0.35 | E 313 | -0.44 | E 313 | -0.22 | E 174 | -0.09 | E 174 | -0.35 | E 209 | -0.42 | E 391 | -0.48 | S 160 | -0.05 | E 116 | -0.40 |
|  | Y 314 | 2.55 | Y 314 | 2.48 | Y 314 | 2.61 | Y 175 | 2.44 | Y 175 | 2.64 | Y 210 | 2.42 | Y 392 | 2.85 | Y 161 | 2.19 | Y 117 | 2.43 |
|  | R 317 | 2.23 | R 317 | 1.17 | R 317 | 2.07 | R 178 | 2.1 | R 178 | 1.67 | R 213 | 1.42 | R 395 | 1.52 | R 164 | 2.06 | R 120 | 0.79 |
|  | T 318 | 1.52 | T 318 | 0.06 | T 318 | 0.16 | T 179 | 0.75 | T 179 | 1.75 | T 214 | 0.46 | T 396 | 1.31 | T 165 | 0.08 | T 121 | 0.00 |
|  | F 320 | 1.02 | F 320 | 0.91 | F 320 | 1.26 | F 181 | 0.97 | F 181 | 1.29 | F 216 | 1.16 | F 398 | 1.25 | F 167 | 1.05 | F 123 | 1.32 |
|  | E 321 | 0.23 | E 321 | 2.48 | E 321 | 1.27 | E 182 | 2.85 | E 182 | 1.93 | E 217 | 1.86 | E 399 | 0.18 | E 168 | 2.30 | E 124 | 0.89 |
|  |  | | | | | | | | | | | | | | | | | |
| CHAIN B | V 282 | 0.17 | V 143 | 0.32 | V 178 | 0.14 | V 143 | 0.21 | V 178 | 0.18 | V 178 | 0.23 | V 360 | 0.26 | V 129 | 0.24 | V 85 | 0.25 |
|  | N 285 | 0.19 | N 146 | 1.89 | N 181 | 0.79 | N 146 | 2.16 | N 181 | 0.03 | N 181 | 0.01 | N 363 | 0.03 | N 132 | 0.92 | N 88 | 0.00 |
|  | H 286 | 1.05 | H 147 | 2.11 | H 182 | 1.87 | H 147 | 3.5 | H 182 | 3.2 | H 182 | 3.53 | H 364 | 1.98 | H 133 | 2.00 | H 89 | 3.30 |
|  | H 288 | 0.45 | Q 149 | 0.2 | H 184 | 0.51 | Q 149 | 0.07 | H 184 | 0.56 | H 184 | 0.46 | H 366 | 0.52 | Q 135 | 0.24 | H 91 | 0.53 |
|  | I 292 | 1.42 | I 153 | 1.84 | I 188 | 1.62 | I 153 | 1.7 | I 188 | 1.58 | I 188 | 1.67 | I 370 | 1.95 | I 139 | 1.92 | I 95 | 1.54 |
|  | L 293 | 1.14 | L 154 | 1.11 | L 189 | 1.19 | L 154 | 1.16 | L 189 | 1.27 | L 189 | 1.01 | L 371 | 1.18 | L 140 | 1.09 | L 96 | 1.09 |
|  | S 295 | 0.99 | S 156 | 0.27 | S 191 | 0.91 | S 156 | 0.54 | S 191 | 0.52 | S 191 | 0.94 | S 373 | 1.02 | S 142 | 1.14 | S 98 | 0.61 |
|  | T 296 | 1.01 | T 157 | 0.79 | T 192 | 0.29 | T 157 | 0.68 | T 192 | 0.52 | T 192 | 0.01 | T 374 | 0.49 | T 143 | 1.11 | T 99 | 0.11 |
|  | F 297 | 3.57 | F 158 | 3.33 | F 193 | 3.39 | F 158 | 3.81 | F 193 | 3.42 | F 193 | 3.66 | F 375 | 3.56 | F 144 | 3.60 | F 100 | 3.35 |
|  |  |  |  |  | H 196 | 0.7 |  |  | H 196 | 0.76 | H 196 | 0.38 |  |  |  |  | E 103 | 0.01 |
|  | K 301 | 2.51 | K 162 | 2.54 | K 197 | 1.74 | K 162 | 2.73 | K 197 | 2.24 | K 197 | 1.66 | K 379 | 1.90 | R 148 | 2.26 | K 104 | 2.35 |
|  | I 306 | 0.3 | I 167 | 0.26 | I 202 | 0.34 | I 167 | 0.37 | I 202 | 0.37 | I 202 | 0.3 | I 384 | 0.37 | I 153 | 0.25 | I 109 | 0.34 |
|  | D 307 | 0.26 | D 168 | 0.39 | D 203 | 0.14 | D 168 | 0.19 | D 203 | -0.13 | D 203 | 0.27 |  |  | D 154 | 0.54 |  |  |
|  | R 309 | 1.7 | R 170 | 0.01 |  |  |  |  |  |  | E 205 | -0.07 | K 387 | -0.15 |  |  |  |  |
|  | T 310 | 0.62 | T 171 | 2.26 | T 206 | 2.22 | T 171 | 0.5 | T 206 | 0.34 | T 206 | 1.17 | T 388 | 0.56 | T 157 | 1.33 | T 113 | 0.34 |
|  | E 313 | -0.05 | E 174 | -0.39 | E 209 | -0.47 | E 174 | 0.48 | E 209 | -0.07 | E 209 | -0.11 | E 391 | -0.39 | S 160 | 0.46 | E 116 | -0.50 |
|  | Y 314 | 2.66 | Y 175 | 2.46 | Y 210 | 2.95 | Y 175 | 2.81 | Y 210 | 2.89 | Y 210 | 2.72 | Y 392 | 2.86 | Y 161 | 2.79 | Y 117 | 2.56 |
|  | R 317 | 1.14 | R 178 | 1.48 | R 213 | 1.23 | R 178 | 2.34 | R 213 | 1.32 | R 213 | 1.41 | R 395 | 1.07 | R 164 | 2.11 | R 120 | 1.00 |
|  | T 318 | 2.22 | T 179 | 0.03 | T 214 | 1.78 | T 179 | 0.05 | T 214 | 1.66 | T 214 | 1.73 | T 396 | 1.77 | T 165 | 1.30 | T 121 | 2.36 |
|  | F 320 | 1.21 | F 181 | 1.22 | F 216 | 1.38 | F 181 | 1.08 | F 216 | 1.12 | F 216 | 1.18 | F 398 | 1.33 | F 167 | 1.01 | F 123 | 1.32 |
|  | E 321 | 0.13 | E 182 | 0.76 | E217 | 0.91 | E 182 | 3.95 | E217 | 2.1 | E 217 | 4.02 | E 399 | 2.43 | E 168 | 1.06 | E 124 | 2.15 |

**Table S5**. Virtual alanine scanning results for HK–HK sensor module models. Hot spots with ΔΔG > 4 kJ/mol colored yellow, hot spots with ΔΔG between 2 and 4 kJ/mol colored red.

| Chain | Residue | Number | KFC2-A | | KFC2-B | | ConSurf | | Rosetta | | PPCheck |
| --- | --- | --- | --- | --- | --- | --- | --- | --- | --- | --- | --- |
|  |  |  | Class | Confidence | Class | Confidence | Class | Value | Class | DDG | Class |
| A | VAL | 143 | ------- | -1.90 | ------- | -0.94 | Conserv | 8 | ------- | 0.27 | ------- |
|  | ASN | 146 | ------- | -1.00 | ------- | -0.88 | Conserv | 9 | ------- | 1.93 | ------- |
|  | HIS | 147 | Hotspot | 0.42 | Hotspot | 0.10 | Conserv | 9 | Hotspot | 2 | Hotspot |
|  | GLN | 149 | ------- | -1.70 | ------- | -0.95 | Conserv | 7 | ------- | 0.35 | ------- |
|  | ALA | 150 | Hotspot | 0.29 | ------- | -0.43 | Conserv | 9 | ------- | --- | ------- |
|  | SER | 152 | ------- | -1.97 | ------- | -0.77 | Conserv | 8 | ------- | --- | ------- |
|  | ILE | 153 | Hotspot | 0.70 | ------- | -0.05 | ------- | 6 | ------- | 1.87 | ------- |
|  | LEU | 154 | Hotspot | 1.22 | Hotspot | 0.27 | Conserv | 7 | ------- | 1.21 | ------- |
|  | SER | 156 | ------- | -0.58 | ------- | -0.75 | Conserv | 8 | ------- | 1.15 | ------- |
|  | THR | 157 | Hotspot | 1.26 | ------- | -0.07 | Conserv | 8 | ------- | 0.91 | ------- |
|  | PHE | 158 | Hotspot | 1.86 | Hotspot | 0.40 | Conserv | 7 | Hotspot | 4 | Hotspot |
|  | GLY | 161 | ------- | -1.78 | ------- | -0.69 | ------- | 1 | ------- | --- | ------- |
|  | LYS | 162 | Hotspot | 0.17 | ------- | -0.20 | ------- | 6 | ------- | 0.75 | ------- |
|  | SER | 165 | ------- | -2.05 | ------- | -0.81 | Conserv | 8 | ------- | --- | ------- |
|  | ALA | 166 | Hotspot | 0.23 | ------- | -0.54 | Conserv | 8 | ------- | --- | ------- |
|  | ILE | 167 | ------- | -1.16 | ------- | -0.89 | ------- | 6 | ------- | 0.29 | ------- |
|  | ASP | 168 | ------- | -1.98 | ------- | -1.00 | Conserv | 7 | ------- | 0.41 | ------- |
|  | ARG | 170 | ------- | -2.35 | ------- | -0.77 | ------- | 1 | ------- | -0.03 | ------- |
|  | THR | 171 | Hotspot | 0.89 | ------- | -0.20 | Conserv | 8 | Hotspot | 2.02 | ------- |
|  | PHE | 172 | ------- | -3.16 | ------- | -0.79 | Conserv | 8 | ------- | --- | ------- |
|  | GLU | 174 | ------- | -0.81 | ------- | -0.72 | ------- | 1 | ------- | -0.09 | ------- |
|  | TYR | 175 | Hotspot | 1.17 | Hotspot | 0.36 | Conserv | 7 | Hotspot | 2.44 | ------- |
|  | ARG | 178 | ------- | -0.36 | ------- | -0.34 | Conserv | 7 | Hotspot | 2.1 | Hotspot |
|  | THR | 179 | Hotspot | 0.40 | ------- | -0.17 | Conserv | 9 | ------- | 0.75 | ------- |
|  | PHE | 181 | ------- | -1.66 | ------- | -0.86 | Conserv | 9 | ------- | 0.97 | ------- |
|  | GLU | 182 | Hotspot | 0.75 | Hotspot | 0.01 | Conserv | 9 | Hotspot | 2.85 | ------- |
|  | ARG | 303 | ------- | -2.37 | ------- | -0.81 | ------- | 1 | ------- | --- | ------- |
|  | | | | | | | | | | | |
| Chain | Residue | Number | KFC2-A | | KFC2-B | | ConSurf | | Rosetta | | PPCheck |
|  |  |  | Class | Confidence | Class | Confidence | Class | Value | Class | DDG | Class |
| B | VAL | 143 | ------- | -2.23 | ------- | -0.99 | Conserv | 8 | ------- | 0.21 | ------- |
|  | ASN | 146 | ------- | -1.20 | ------- | -0.83 | Conserv | 9 | Hotspot | 2.16 | ------- |
|  | HIS | 147 | Hotspot | 0.42 | Hotspot | 0.09 | Conserv | 9 | Hotspot | 3.5 | Hotspot |
|  | GLN | 149 | ------- | -2.19 | ------- | -0.97 | Conserv | 7 | ------- | 0.07 | ------- |
|  | ALA | 150 | Hotspot | 0.37 | ------- | -0.53 | Conserv | 9 | ------- | --- | ------- |
|  | ILE | 153 | Hotspot | 0.66 | ------- | -0.20 | ------- | 6 | ------- | 1.7 | ------- |
|  | LEU | 154 | Hotspot | 0.76 | Hotspot | 0.18 | Conserv | 7 | ------- | 1.16 | Hotspot |
|  | SER | 156 | ------- | -0.99 | ------- | -0.77 | Conserv | 8 | ------- | 0.54 | ------- |
|  | THR | 157 | Hotspot | 1.49 | ------- | -0.07 | Conserv | 8 | ------- | 0.68 | Hotspot |
|  | PHE | 158 | Hotspot | 1.88 | Hotspot | 0.39 | Conserv | 7 | Hotspot | 3.81 | Hotspot |
|  | GLY | 161 | ------- | -1.09 | ------- | -0.69 | ------- | 1 | ------- | --- | ------- |
|  | LYS | 162 | ------- | -0.14 | ------- | -0.33 | ------- | 6 | Hotspot | 2.73 | ------- |
|  | SER | 165 | ------- | -1.95 | ------- | -0.82 | Conserv | 8 | ------- | --- | ------- |
|  | ALA | 166 | ------- | -0.46 | ------- | -0.70 | Conserv | 8 | ------- | --- | ------- |
|  | ILE | 167 | ------- | -1.44 | ------- | -0.86 | ------- | 6 | ------- | 0.37 | ------- |
|  | ASP | 168 | ------- | -2.13 | ------- | -1.00 | Conserv | 7 | ------- | 0.19 | ------- |
|  | ARG | 170 | ------- | -2.46 | ------- | -0.75 | ------- | 1 | ------- | --- | ------- |
|  | THR | 171 | Hotspot | 0.87 | ------- | -0.25 | Conserv | 8 | ------- | 0.5 | ------- |
|  | GLU | 174 | ------- | -0.80 | ------- | -0.64 | ------- | 1 | ------- | 0.48 | ------- |
|  | TYR | 175 | Hotspot | 1.56 | Hotspot | 0.38 | Conserv | 7 | Hotspot | 2.81 | Hotspot |
|  | ARG | 178 | ------- | -0.71 | ------- | -0.37 | Conserv | 7 | Hotspot | 2.34 | Hotspot |
|  | THR | 179 | Hotspot | 0.04 | ------- | -0.24 | Conserv | 9 | ------- | 0.05 | ------- |
|  | PHE | 181 | ------- | -1.56 | ------- | -0.81 | Conserv | 9 | ------- | 1.08 | ------- |
|  | GLU | 182 | Hotspot | 0.55 | Hotspot | 0.02 | Conserv | 9 | Hotspot | 3.95 | ------- |
|  | ARG | 303 | ------- | -2.52 | ------- | -0.80 | ------- | 1 | ------- | --- | ------- |

**Table S6**. Comparison of KFC-2 server hot spot prediction with Rosetta and PPCheck results for AHK3 sensor module homodimer with addition of ConSurf conservation scores.

**Table S7**. Involvement in Hydrogen bond, Salt bridge, Disulphide bond, or Covalent link (HSDC), ASA - Accessible Surface Area (Å²), BSA - Buried Surface Area (Å²), Buried area percentage (% of BSA) and ΔiG - Solvation energy effect (kcal/mol) of interface residues of AHK3 sensor module homodimer.

| Chain A | HSDC | ASA | BSA | % of BSA | ΔiG |
| --- | --- | --- | --- | --- | --- |
| A:VAL 143 |  | 67.95 | 26.95 | 40 | 0.43 |
| A:ASN 146 | H | 93.95 | 45.13 | 48 | -0.07 |
| A:HIS 147 |  | 61.56 | 55.61 | 90 | 1.13 |
| A:GLN 149 |  | 76.58 | 22.71 | 30 | -0.12 |
| A:ALA 150 | H | 50.50 | 42.2 | 84 | 0.48 |
| A:SER 152 |  | 11.69 | 0.24 | 2 | -0.00 |
| A:ILE 153 |  | 111.56 | 91.99 | 82 | 1.47 |
| A:LEU 154 |  | 33.25 | 32.75 | 98 | 0.52 |
| A:SER 156 | H | 43.68 | 27.3 | 63 | -0.19 |
| A:THR 157 |  | 71.74 | 69.48 | 97 | 0.75 |
| A:PHE 158 |  | 91.02 | 90.86 | 100 | 1.45 |
| A:GLY 161 |  | 37.64 | 5.01 | 13 | 0.08 |
| A:LYS 162 | HS | 115.00 | 73.27 | 64 | 0.31 |
| A:SER 165 |  | 50.22 | 0.37 | 1 | -0.00 |
| A:ALA 166 | H | 41.64 | 38.69 | 93 | 0.30 |
| A:ILE 167 |  | 13.42 | 4.01 | 30 | 0.06 |
| A:ASP 168 | HS | 71.22 | 12.28 | 17 | -0.21 |
| A:ARG 170 |  | 178.82 | 8.89 | 5 | -0.10 |
| A:THR 171 | H | 52.69 | 50.16 | 95 | 0.50 |
| A:GLU 174 |  | 80.28 | 36.69 | 46 | -0.08 |
| A:TYR 175 |  | 85.76 | 79.73 | 93 | 0.43 |
| A:ARG 178 | H | 169.22 | 85.02 | 50 | -1.21 |
| A:THR 179 |  | 25.27 | 22.59 | 89 | 0.36 |
| A:PHE 181 |  | 148.84 | 42.36 | 28 | 0.66 |
| A:GLU 182 | H | 47.37 | 46.52 | 98 | -0.38 |
| A:ARG 303 | S | 204.53 | 35.11 | 17 | -0.54 |
|  | | | | | |
| Chain B | HSDC | ASA | BSA | % of BSA | ΔiG |
| B:VAL 143 |  | 64.56 | 20.08 | 31 | 0.32 |
| B:ASN 146 | H | 97.05 | 52.01 | 54 | -0.05 |
| B:HIS 147 |  | 60.16 | 52.19 | 87 | 1.16 |
| B:GLN 149 |  | 71.02 | 17.24 | 24 | 0.16 |
| B:ALA 150 | H | 56.40 | 47.71 | 85 | 0.65 |
| B:ILE 153 |  | 110.78 | 82.67 | 75 | 1.32 |
| B:LEU 154 |  | 34.77 | 31.92 | 92 | 0.51 |
| B:SER 156 | H | 42.72 | 23.49 | 55 | -0.11 |
| B:THR 157 |  | 68.37 | 67.37 | 99 | 0.46 |
| B:PHE 158 |  | 89.12 | 88.50 | 99 | 1.42 |
| B:GLY 161 |  | 28.01 | 15.56 | 56 | 0.22 |
| B:LYS 162 | HS | 105.55 | 58.83 | 56 | -1.41 |
| B:SER 165 |  | 49.38 | 1.35 | 3 | -0.02 |
| B:ALA 166 |  | 48.23 | 35.52 | 74 | 0.17 |
| B:ILE 167 |  | 14.21 | 4.94 | 35 | 0.07 |
| B:ASP 168 | HS | 73.69 | 20.01 | 27 | -0.34 |
| B:ARG 170 |  | 177.26 | 6.11 | 3 | -0.12 |
| B:THR 171 |  | 66.9 | 60.82 | 91 | 0.58 |
| B:GLU 174 | S | 76.01 | 42.81 | 56 | -0.16 |
| B:TYR 175 | H | 100.41 | 93.69 | 93 | 0.60 |
| B:ARG 178 | H | 171.68 | 86.66 | 50 | -1.61 |
| B:THR 179 |  | 21.41 | 17.60 | 82 | -0.04 |
| B:PHE 181 |  | 148.26 | 51.03 | 34 | 0.82 |
| B:GLU 182 | H | 47.04 | 45.51 | 97 | -0.40 |
| B:ARG 303 |  | 180.28 | 18.27 | 10 | -0.21 |

**Table S8**. Sequence identity between *Arabidopsis thaliana* and *Solanum tuberosum* MSP components. Values for orthologs are in bold.

|  | StHK2sm | StHK3sm | StHK4sm |
| --- | --- | --- | --- |
| AHK2sm | **78%** | 65% | 64% |
| AHK3sm | 70% | **80%** | 63% |
| AHK4sm | 61% | 61% | **79%** |
|  | | | |
|  | StHK2hk | StHK3hk | StHK4hk |
| AHK2hk | **79%** | 73% | 67% |
| AHK3hk | 72% | **84%** | 65% |
| AHK4hk | 67% | 70% | **85%** |
|  | | | |
|  | StHK2rd | StHK3rd | StHK4rd |
| AHK2rd | **76%** | 66% | 71% |
| AHK3rd | 65% | **75%** | 70% |
| AHK4rd | 63% | 60% | **71%** |
|  | | | |
|  | StHP1a | | |
| AHP1 | **70%** | | |
| AHP2 | 59% | | |
| AHP3 | 60% | | |
|  | | | |
|  | StRR1a_rd | StRR11rd | |
| ARR1rd | **89%** | 71% | |
| ARR2rd | 88% | 72% | |
| ARR10rd | 72% | 67% | |
| ARR11rd | 73% | **93%** | |

|  | AHK2_HisKA_-AHK2_HisKA_ | | AHK2_HisKA_-AHK3_HisKA_ | | AHK2_HisKA_-AHK4_HisKA_ | | AHK3_HisKA_-AHK3_HisKA_ | | AHK3_HisKA_-AHK4_HisKA_ | | AHK4_HisKA_-AHK4_HisKA_ | | StHK2_HisKA_-StHK2_HisKA_ | | StHK3_HisKA_-StHK3_HisKA_ | | StHK4_HisKA_-StHK4_HisKA_ | |
| --- | --- | --- | --- | --- | --- | --- | --- | --- | --- | --- | --- | --- | --- | --- | --- | --- | --- | --- |
|  | mutated residue | ΔΔG of complex (kJ/mol) | mutated residue | ΔΔG of complex (kJ/mol) | mutated residue | ΔΔG of complex (kJ/mol) | mutated residue | ΔΔG of complex (kJ/mol) | mutated residue | ΔΔG of complex (kJ/mol) | mutated residue | ΔΔG of complex (kJ/mol) | mutated residue | ΔΔG of complex (kJ/mol) | mutated residue | ΔΔG of complex (kJ/mol) | mutated residue | ΔΔG of complex (kJ/mol) |
| CHAIN A |  |  |  |  |  |  | I 412 | 0.24 |  |  |  |  |  |  |  |  |  |  |
|  |  |  |  |  |  |  |  |  |  |  |  |  |  |  | L 401 | 0.92 |  |  |
|  | V 553 | 0.37 |  |  | V 553 | 0.57 | V 416 | 0.04 |  |  |  |  |  |  |  |  |  |  |
|  | I 556 | 0.26 | I 556 | 0.62 | I 556 | 0.55 | I 419 | 0.64 |  |  |  |  |  |  |  |  |  |  |
|  | L 557 | 1.04 | L 557 | 0.52 | L 557 | 0.47 | I 420 | 0.21 | I 420 | 0.54 | L 442 | 0.79 | F 635 | 0.52 | F 407 | 1.46 |  |  |
|  |  |  |  |  |  |  | T 423 | 0.48 | T 423 | 0.14 |  |  |  |  | T 410 | 1.07 | S 351 | -0.08 |
|  | I 561 | 0.08 | I 561 | 0.02 | I 561 | 0.03 |  |  | V 424 | 0.15 |  |  | I 639 | 0.02 | I 411 | 0.07 |  |  |
|  | R 563 | 0.84 | R 563 | 1.88 | R 563 | 1.23 | R 426 | 0.11 | R 426 | 1.32 | H 448 | 1.66 | R 641 | 0.07 | R 413 | 0.07 | H 354 | 1.18 |
|  | I 564 | 1.49 | I 564 | 1.53 | I 564 | 1.31 | I 427 | 1.45 | I 427 | 1.67 | I 449 | 1.73 | I 642 | 1.39 | I 414 | 1.71 | I 355 | 1.23 |
|  |  |  |  |  |  |  | K 429 | 0.34 | K 429 | 0 | K 451 | -0.08 |  |  |  |  | K 357 | -0.03 |
|  | V 567 | 1.15 | V 567 | 1 | V 567 | 1.13 | V 430 | 0.85 | V 430 | 1.1 | V 452 | 0.89 | V 645 | 0.98 | V 417 | 0.95 | V 358 | 0.85 |
|  | E 568 | 0.42 | E 568 | 0.34 | E 568 | 0.49 | E 431 | 0.23 | E 431 | -0.02 | E 453 | 1.51 | E 646 | 0.64 | E 418 | 1.34 | E 359 | 0.62 |
|  | D 570 | -0.18 | D 570 | -0.24 | D 570 | -0.09 | D 433 | -0.2 | D 433 | -0.1 | D 455 | -0.11 | Q 648 | 0.19 | D 420 | -0.07 | D 361 | -0.13 |
|  | C 571 | -0.11 | C 571 | -0.12 | C 571 | -0.12 | C 434 | -0.19 | C 434 | 0.04 | F 456 | 1.81 | Y 649 | 0.66 | Y 421 | 0.56 | F 362 | 1.32 |
|  |  |  |  |  |  |  |  |  |  |  | H 457 | 0.67 |  |  |  |  |  |  |
|  | K 573 | 0 |  |  |  |  | K 436 | -0.01 |  |  |  |  |  |  | E 423 | 0.14 |  |  |
|  | M 574 | 0.48 | M 574 | 0.44 | M 574 | 1.07 | M 437 | 0.71 | M 437 | 0.74 | M 459 | 1 | M 652 | 0.76 | M 424 | 1.03 | M 365 | 1.24 |
|  | R 575 | 0.14 | R 575 | 0.11 | R 575 | 0.52 | K 438 | -0.15 | K 438 | 0.04 | Q 460 | 0.13 | M 653 | 0.02 | M 425 | 0.06 | Q 366 | 0.09 |
|  | L 577 | 0.09 |  |  | L 577 | 0 | L 440 | -0.01 | L 440 | 0.01 | L 462 | 0.08 | L 655 | 0.13 | L 427 | 0.06 | L 368 | -0.03 |
|  | K 578 | 0.33 | K 578 | 0.43 | K 578 | 0.58 | K 441 | 0.59 | K 441 | 0.69 | K 463 | 1.23 | K 656 | 0.34 | K 428 | 0.41 | K 369 | 0.60 |
|  |  |  |  |  |  |  |  |  | K 443 | 0.17 |  |  |  |  |  |  |  |  |
|  | E 582 | -0.01 |  |  | E 582 | -0.03 | E 445 | -0.01 |  |  |  |  | E 660 | -0.01 |  |  |  |  |
|  |  |  |  |  | D 585 | -0.17 | D 448 | -0.26 | D 448 | -0.18 | D 470 | 0.75 | D 663 | -0.15 | D 435 | -0.02 | D 376 | 0.92 |
|  | K 588 | 1.51 | K 588 | 0.97 | K 588 | 0.75 | K 451 | 3.38 | K 451 | 2.38 | K 473 | 1.04 | K 666 | 4.77 | K 438 | 2.55 | K 379 | 3.48 |
|  | F 591 | 1.18 | F 591 | 1.41 | F 591 | 2 | F 454 | 1.99 | F 454 | 1.05 | F 476 | 1.47 | F 669 | 1.43 | F 441 | 1.59 | F 382 | 1.82 |
|  | L 592 | 0.26 | L 592 | 0.6 | L 592 | 0.64 | L 455 | 0.74 | L 455 | 0.62 | L 477 | 0.76 | L 670 | 0.87 | L 442 | 0.76 | L 383 | 0.70 |
|  | V 595 | 0.62 | V 595 | 0.64 | V 595 | 0.65 | V 458 | 0.88 | V 458 | 0.73 | V 480 | 0.89 | V 673 | 0.74 | V 445 | 0.64 | V 386 | 0.95 |
|  | E 598 | -0.01 |  |  | E 598 | -0.02 |  |  | E 461 | 0.01 | E 483 | -0.03 | E 676 | 0.01 |  |  | E 389 | 1.70 |
|  | I 599 | 1.21 | I 599 | 0.9 | I 599 | 1.38 | I 462 | 1.33 | I 462 | 0.41 | I 484 | 0.63 | I 677 | 1.24 | I 449 | 1.02 | I 390 | 1.26 |
|  | T 601 | 0 |  |  |  |  | T 464 | 0 |  |  |  |  |  |  |  |  | T 392 | 0.00 |
|  | M 603 | 0.32 | M 603 | 0.21 | M 603 | 0.58 | M 466 | 0.63 | M 466 | 0.28 | M 488 | 0.32 | M 681 | 0.21 | M 453 | 0.29 | M 394 | 0.26 |
|  | V 606 | 0.82 | V 606 | 0.68 | V 606 | 0.92 | V 469 | 0.69 | V 469 | 0.86 | I 491 | 1.84 | V 684 | 0.69 | V 456 | 0.72 | I 397 | 1.48 |
|  | M 609 | 1.02 | M 609 | 0.68 | M 609 | 0.75 | M 472 | 0.65 | M 472 | 0.87 | M 494 | 1.16 | M 687 | 0.98 | M 459 | 1.34 | M 400 | 0.85 |
|  | L 610 | 0.97 | L 610 | 0.68 | L 610 | 0.88 | L 473 | 0.6 | L 473 | 0.83 | L 495 | 0.97 | L 688 | 0.90 | L 460 | 0.63 | L 401 | 0.54 |
|  | M 612 | 0.12 | M 612 | 0.08 | M 612 | 0.05 | M 475 | 0.23 | M 475 | 0.17 | M 497 | 0.28 | M 690 | 0.12 | M 462 | 0.30 | L 403 | 0.35 |
|  | L 613 | 2.07 | L 613 | 1.61 | L 613 | 1.87 | L 476 | 1.41 | L 476 | 1.7 | L 498 | 1.48 | L 691 | 1.70 | L 463 | 1.64 | L 404 | 1.78 |
|  | T 616 | 1.01 | T 616 | 0.67 | T 616 | 0.71 | T 479 | 1.03 | T 479 | 0.82 | T 501 | 0.94 | T 694 | 0.57 | T 466 | 1.12 | T 407 | 1.41 |
|  | L 618 | 0.33 | L 618 | 0.64 | L 618 | 0.39 | L 481 | 0.57 | L 481 | 0.42 | L 503 | 0.52 | L 696 | 0.20 | L 468 | 0.72 | L 409 | 0.36 |
|  |  |  | D 619 | 1.26 | D 619 | 0.98 | D 482 | 1.61 |  |  | S 504 | 0 |  |  | D 469 | -0.01 | S 410 | 0.00 |
|  | K 621 | 0 | K 621 | 0.48 | K 621 | 0.16 |  |  | T 484 | 0.02 | T 506 | 0.17 | T 699 | 0.08 |  |  | T 412 | 0.05 |
|  | Q 622 | 2.34 | Q 622 | 2.47 | Q 622 | 2.31 | Q 485 | 2.37 | Q 485 | 1.32 | Q 507 | 2.32 | Q 700 | 2.49 | Q 472 | 2.40 | Q 413 | 2.24 |
|  | Y 625 | 1.4 | Y 625 | 2.05 | Y 625 | 1.22 | Y 488 | 1.69 | Y 488 | 1.02 | Y 510 | 1.29 | Y 703 | 0.90 | Y 475 | 1.24 | Y 416 | 1.54 |
|  |  |  |  |  |  |  | V 489 | 0.24 | V 489 | 0.26 |  |  |  |  | V 476 | 0.26 |  |  |
|  | T 628 | 0.03 | T 628 | 0.18 | T 628 | 0.04 | T 491 | 0.09 | T 491 | 0.13 | T 513 | 0.17 | T 706 | 0.14 | T 478 | 0.02 | T 419 | -0.03 |
|  | S 632 | -0.06 | S 632 | -0.07 | S 632 | -0.17 | S 495 | -0.13 | S 495 | -0.14 | C 517 | -0.13 | S 710 | -0.17 | S 482 | -0.13 | C 423 | -0.05 |
|  |  |  |  |  |  |  |  |  |  |  |  |  | D 713 | -0.08 |  |  |  |  |
|  | L 636 | 2.46 | L 636 | 1.92 | L 636 | 1.94 | L 499 | 1.88 | L 499 | 1.88 | L 521 | 2 | L 714 | 2.42 | L 486 | 2.04 | L 427 | 2.33 |
|  |  |  |  |  |  |  | V 500 | 0 |  |  |  |  |  |  |  |  |  |  |
|  | L 639 | 0.95 | L 639 | 0.28 | L 639 | 1.13 | L 502 | 0.76 | L 502 | 1.24 | L 524 | 1.3 | L 717 | 1.11 | L 489 | 0.64 | L 430 | 1.03 |
|  | I 640 | 0.5 | I 640 | 1.17 | I 640 | 0.78 | I 503 | 1.38 | I 503 | 1.07 | I 525 | 1.04 | I 718 | 1.32 | I 490 | 1.23 | I 431 | 1.22 |
|  | V 643 | 0.92 | V 643 | 1.17 | V 643 | 0.89 | V 506 | 1.01 | V 506 | 1.25 | V 528 | 1.26 | V 721 | 1.36 | V 493 | 1.29 | V 434 | 1.05 |
|  | L 644 | 0.87 | L 644 | 0.74 | L 644 | 0.86 | L 507 | 0.74 |  |  | L 529 | 0.98 | L 722 | 0.84 | L 494 | 0.90 | L 435 | 0.93 |
|  | Q 646 | 0.74 | Q 646 | 0.03 | Q 646 | 0.6 | Q 509 | 0.63 | Q 509 | 0.21 | R 531 | 0.08 | Q 724 | 0.64 | Q 496 | 0.38 | R 437 | 0.23 |
|  |  |  |  |  |  |  |  |  |  |  |  |  |  |  |  |  | K 439 | -0.05 |
|  | I 649 | 0.59 | I 649 | 0.76 | I 649 | 1.1 | I 512 | 0.81 | I 512 | 1.02 | I 534 | 1.12 | I 727 | 1.12 | I 499 | 0.86 | I 440 | 1.05 |
|  | E 650 | 0.62 | E 650 | 0.38 | E 650 | 0.61 | E 513 | 0.32 |  |  |  |  | E 728 | 0.24 | E 500 | 0.40 | E 441 | 0.73 |
|  |  |  |  |  |  |  | S 514 | 0.67 |  |  |  |  | S 729 | 0.57 | S 501 | -0.01 |  |  |

**Table S9 (beginning)**. Virtual alanine scanning results for chains A of modeled HisKA domain dimers. Hot spots with ΔΔG > 4 kJ/mol colored yellow, hot spots with ΔΔG between 2 and 4 kJ/mol are red.

**Table S9 (ending)**. Virtual alanine scanning results for chains B of modeled HisKA domain dimers. Hot spots with ΔΔG > 4 kJ/mol colored yellow, hot spots with ΔΔG between 2 and 4 kJ/mol are red.

|  | AHK2_HisKA_-AHK2_HisKA_ | | AHK2_HisKA_-AHK3_HisKA_ | | AHK2_HisKA_-AHK4_HisKA_ | | AHK3_HisKA_-AHK3_HisKA_ | | AHK3_HisKA_-AHK4_HisKA_ | | AHK4_HisKA_-AHK4_HisKA_ | | StHK2_HisKA_-StHK2_HisKA_ | | StHK3_HisKA_-StHK3_HisKA_ | | StHK4_HisKA_-StHK4_HisKA_ | |
| --- | --- | --- | --- | --- | --- | --- | --- | --- | --- | --- | --- | --- | --- | --- | --- | --- | --- | --- |
|  | mutated residue | ΔΔG of complex (kJ/mol) | mutated residue | ΔΔG of complex (kJ/mol) | mutated residue | ΔΔG of complex (kJ/mol) | mutated residue | ΔΔG of complex (kJ/mol) | mutated residue | ΔΔG of complex (kJ/mol) | mutated residue | ΔΔG of complex (kJ/mol) | mutated residue | ΔΔG of complex (kJ/mol) | mutated residue | ΔΔG of complex (kJ/mol) | mutated residue | ΔΔG of complex (kJ/mol) |
| CHAIN B |  |  |  |  |  |  | L 414 | 0.62 |  |  | F 436 | 1.12 |  |  |  |  |  |  |
|  | L 552 | 0.01 |  |  |  |  | L 415 | 0.54 |  |  |  |  |  |  |  |  |  |  |
|  | V 553 | 1.15 |  |  |  |  | V 416 | 0 |  |  |  |  |  |  |  |  |  |  |
|  |  |  |  |  | Y 440 | 0.88 |  |  |  |  | Y 440 | 1.49 |  |  |  |  |  |  |
|  | I 556 | 0.05 | I 419 | 0.23 | I 441 | 0.09 | I 419 | 0.46 |  |  |  |  | I 634 | 0.25 | I 406 | 0.31 | T 347 | 0.36 |
|  | L 557 | 1.52 | I 420 | 1.46 | L 442 | 1.54 | I 420 | 0.99 | L 442 | 0.86 | L 442 | 0.66 | F 635 | 1.08 | F 407 | 2.55 |  |  |
|  |  |  | T 423 | 0.44 |  |  | T 423 | 0.59 |  |  |  |  |  |  | T 410 | 1.33 | S 351 | -0.12 |
|  |  |  | V 424 | 0.12 |  |  |  |  |  |  |  |  |  |  | I 411 | 0.04 |  |  |
|  | R 563 | 0.51 | R 426 | 0.56 | H 448 | 1 | R 426 | 0.21 | H 448 | 0.08 | H 448 | 0.12 | R 641 | 0.93 | R 413 | 1.70 | H 354 | 0.98 |
|  | I 564 | 1.45 | I 427 | 1.85 | I 449 | 1.2 | I 427 | 1.16 | I 449 | 1.7 | I 449 | 1.56 | I 642 | 1.27 | I 414 | 1.30 | I 355 | 1.95 |
|  |  |  |  |  | K 451 | -0.15 | K 429 | 0.23 | K 451 | 0 |  |  |  |  |  |  |  |  |
|  | V 567 | 0.84 | V 430 | 0.76 | V 452 | 1.45 | V 430 | 0.71 | V 452 | 0.92 | V 452 | 0.95 | V 645 | 0.41 | V 417 | 0.70 | V 358 | 0.93 |
|  | E 568 | 0.95 | E 431 | 1.52 | E 453 | 1.22 | E 431 | 0.14 | E 453 | 1.31 | E 453 | 0.88 |  |  | E 418 | -0.08 | E 359 | 0.43 |
|  | D 570 | -0.07 | D 433 | -0.18 | D 455 | 0.25 | D 433 | -0.25 | D 455 | -0.06 | D 455 | -0.06 | Q 648 | 0.01 | D 420 | -0.05 | D 361 | -0.08 |
|  | C 571 | -0.09 | C 434 | -0.1 | F 456 | 1.43 | C 434 | -0.12 | F 456 | 1.54 | F 456 | 1.19 | Y 649 | 2.62 | Y 421 | 2.63 | F 362 | 1.68 |
|  |  |  |  |  |  |  | D 435 | -0.03 |  |  |  |  |  |  |  |  |  |  |
|  |  |  |  |  | E458 | 0.08 |  |  |  |  |  |  |  |  |  |  |  |  |
|  | M 574 | 0.79 | M 437 | 0.79 | M 459 | 0.3 | M 437 | 0.82 | M 459 | 0.83 | M 459 | 1.26 | M 652 | 0.29 | M 424 | 1.13 | M 365 | 1.00 |
|  | R 575 | 0.25 | K 438 | -0.01 | Q 460 | 0.2 | K 438 | 0.13 | Q 460 | 0.06 | Q 460 | 0.05 | M 653 | 0.19 | M 425 | 0.05 | Q 366 | 0.06 |
|  | L 577 | 0.03 | L 440 | 0.3 | L 462 | 0.41 | L 440 | -0.03 | L 462 | 0.11 | L 462 | 0 | L 655 | 0.04 | L 427 | 0.22 | L 368 | 0.04 |
|  | K 578 | 0.89 | K 441 | 0.66 | K 463 | 0.45 | K 441 | 0.9 | K 463 | 0.7 | K 463 | 0.96 | K 656 | 0.70 | K 428 | 0.79 | K 369 | 0.63 |
|  |  |  | K 443 | -0.04 |  |  | K 443 | 0.26 |  |  |  |  | R 658 | 0.04 | R 430 | 0.55 |  |  |
|  |  |  |  |  |  |  | E 445 | -0.06 |  |  | Q 467 | -0.07 |  |  | E 432 | -0.13 | E 373 | -0.10 |
|  | D 585 | 1.45 | D 448 | 0.63 | D 470 | 0.41 | D 448 | 2.93 | D 470 | 2.12 | D 470 | 1.97 | D 663 | 2.36 | D 435 | 0.46 | D 376 | 2.13 |
|  | K 588 | 1.63 | K 451 | 1.61 | K 473 | 1.87 | K 451 | 2.67 | K 473 | 0.8 | K 473 | 2.25 | K 666 | 1.62 | K 438 | 2.90 | K 379 | 2.92 |
|  | S 589 | -0.01 |  |  | S 474 | -0.03 | S 452 | 0.35 | S 474 | 1.32 | S 474 | 1.55 | S 667 | 1.71 | S 439 | -0.03 | S 380 | -0.01 |
|  |  |  |  |  | F 476 | 1.49 | F 454 | 0.78 | F 476 | 0.82 | F 476 | 1.13 | F 669 | 1.16 | F 441 | 1.19 | F 382 | 1.24 |
|  | L 592 | 1.1 | L 455 | 1.03 | L 477 | 1.39 | L 455 | 1.32 | L 477 | 1.5 | L 477 | 1.42 | L 670 | 1.15 | L 442 | 1.34 | L 383 | 1.16 |
|  | V 595 | 0.39 | V 458 | 0.28 | V 480 | 0.57 | V 458 | 0.52 | V 480 | 0.8 | V 480 | 0.43 | V 673 | 0.65 | V 445 | 0.49 | V 386 | 0.52 |
|  | E 598 | -0.03 | E 461 | 0 | E 483 | -0.01 | E 461 | 0.02 | E 483 | -0.01 | E 483 | -0.02 | E 676 | -0.01 | E 448 | 0.00 | E 389 | -0.01 |
|  | I 599 | 0.82 | I 462 | 1.75 | I 484 | 1.03 | I 462 | 1.16 | I 484 | 1.19 | I 484 | 1.44 | I 677 | 1.23 | I 449 | 1.99 | I 390 | 1.46 |
|  | M 603 | 0.66 | M 466 | 0.32 | M 488 | 0.53 | M 466 | 0.53 | M 488 | 0.52 | M 488 | 0.52 | M 681 | 0.27 | M 453 | 0.60 | M 394 | 0.41 |
|  | V 606 | 0.97 | V 469 | 0.76 | I 491 | 1.56 | V 469 | 1.09 | I 491 | 1.8 | I 491 | 1.95 | V 684 | 0.79 | V 456 | 0.81 | I 397 | 1.54 |
|  | M 609 | 0.72 | M 472 | 0.6 | M 494 | 1.18 | M 472 | 0.75 | M 494 | 1.3 | M 494 | 0.67 | M 687 | 1.53 | M 459 | 0.72 | M 400 | 0.84 |
|  | L 610 | 0.94 | L 473 | 0.81 | L 495 | 0.73 | L 473 | 0.18 | L 495 | 0.21 | L 495 | 0.94 | L 688 | 0.54 | L 460 | 0.88 | L 401 | 0.19 |
|  | M 612 | 0.17 | M 475 | 0.11 |  |  | M 475 | 0.15 | M 497 | 0.1 |  |  |  |  | M 462 | 0.15 | L 403 | 0.57 |
|  | L 613 | 1.63 | L 476 | 1.73 | L 498 | 1.72 | L 476 | 1.93 | L 498 | 1.62 | L 498 | 1.89 | L 691 | 1.25 | L 463 | 1.62 | L 404 | 1.54 |
|  | T 616 | 0.72 | T 479 | 0.95 | T 501 | 0.75 | T 479 | 0.61 | T 501 | 0.14 | T 501 | 0.68 | T 694 | 0.73 | T 466 | 0.90 | T 407 | 1.50 |
|  | L 618 | 0.34 | L 481 | 0.52 | L 503 | 0.5 | L 481 | 0.7 | L 503 | 0.34 | L 503 | 0.51 | L 696 | 0.32 | L 468 | 0.46 | L 409 | 0.41 |
|  | D 619 | -0.01 | D 482 | 1.03 |  |  | D 482 | -0.02 | S 504 | 0 | S 504 | -0.01 |  |  | D 469 | 2.23 | S 410 | 0.00 |
|  | K 621 | -0.08 | T 484 | 0.07 | T 506 | 0.04 | T 484 | 0 | T 506 | 0.06 | T 506 | 0.15 | T 699 | 0.13 | T 471 | 0.66 | T 412 | 0.03 |
|  | Q 622 | 1.32 | Q 485 | 2.45 | Q 507 | 2.34 | Q 485 | 2.22 | Q 507 | 2.43 | Q 507 | 2.25 | Q 700 | 1.33 | Q 472 | 2.31 | Q 413 | 2.23 |
|  | Y 625 | 1.66 | Y 488 | 0.86 | Y 510 | 1.21 | Y 488 | 1.12 | Y 510 | 1.48 | Y 510 | 1.02 | Y 703 | 1.73 | Y 475 | 0.96 | Y 416 | 1.60 |
|  |  |  | V 489 | 0.14 |  |  | V 489 | 0.25 |  |  |  |  |  |  | V 476 | 0.33 |  |  |
|  | T 628 | -0.01 | T 491 | -0.01 | T 513 | 0.02 | T 491 | -0.04 | T 513 | 0.09 | T 513 | 0.08 | T 706 | 0.01 | T 478 | 0.10 | T 419 | 0.15 |
|  | S 632 | -0.17 | S 495 | -0.13 | C 517 | -0.09 | S 495 | -0.17 | C 517 | -0.05 | C 517 | -0.14 | S 710 | -0.18 | S 482 | -0.13 | C 423 | -0.06 |
|  | D 635 | -0.05 |  |  |  |  |  |  |  |  |  |  | D 713 | -0.10 |  |  | S 426 | -0.02 |
|  | L 636 | 1.47 | L 499 | 1.7 | L 521 | 2.39 | L 499 | 1.87 | L 521 | 1.67 | L 521 | 2.43 | L 714 | 1.35 | L 486 | 2.23 | L 427 | 2.28 |
|  |  |  |  |  |  |  |  |  |  |  |  |  |  |  |  |  | R 429 | 1.67 |
|  | L 639 | 1.19 | L 502 | 1.15 | L 524 | 1.2 | L 502 | 1.05 | L 524 | 0.71 | L 524 | 1.39 | L 717 | 1.26 | L 489 | 2.02 | L 430 | 1.92 |
|  | I 640 | 0.27 | I 503 | 0.57 | I 525 | 0.51 | I 503 | 0.67 | I 525 | 0.47 | I 525 | 0.65 | I 718 | 0.63 | I 490 | 0.73 | I 431 | 0.57 |
|  | E 642 | -0.06 |  |  | E 527 | 0 | 505 | 0.15 | E 527 | 0.14 | E 527 | 0.01 |  |  | E 492 | 0.14 | E 433 | -0.02 |
|  | V 643 | 0.82 | V 506 | 0.73 | V 528 | 1.18 | V 506 | 1.06 | V 528 | 0.78 | V 528 | 1.1 | V 721 | 1.17 | V 493 | 0.78 | V 434 | 1.19 |
|  | Q 646 | 1.35 | Q 509 | 1.35 | R 531 | 0.92 | Q 509 | 1.12 | R 531 | 0.48 | R 531 | 2.46 | Q 724 | 0.99 | Q 496 | 2.02 | R 437 | 0.86 |
|  | I 649 | 0.12 | I 512 | 0.09 | I 534 | 0.03 |  |  | I 534 | -0.03 | I 534 | 0.11 | I 727 | 0.11 | I 499 | 0.14 | I 440 | 0.23 |
|  | E 650 | -0.14 | E 513 | 0.11 | E 535 | -0.27 | E 513 | 0.18 | E 535 | 0.17 |  |  | E 728 | 0.30 | E 500 | 0.53 | E 441 | -0.34 |

| Chain | Residue | Number | KFC2-A | | KFC2-B | | ConSurf | | Rosetta | | PPCheck |
| --- | --- | --- | --- | --- | --- | --- | --- | --- | --- | --- | --- |
|  |  |  | Class | Confidence | Class | Confidence | Class | Value | Class | ΔΔG | Class |
| A | ILE | 412 | ------- | -2.91 | ------- | -0.93 | Conserv | 7 | ------- | 0.24 |  |
|  | ALA | 413 | ------- | -1.99 | ------- | -0.84 | Conserv | 7 | ------- | --- |  |
|  | VAL | 416 | ------- | -3.07 | ------- | -0.84 | ------- | 4 | ------- | 0.04 |  |
|  | ILE | 419 | ------- | -0.79 | ------- | -0.37 | ------- | 1 | ------- | 0.64 |  |
|  | ILE | 420 | ------- | -2.54 | ------- | -0.99 | ------- | 1 | ------- | 0.21 |  |
|  | ALA | 422 | ------- | -2.98 | ------- | -0.78 | ------- | 2 | ------- | --- |  |
|  | THR | 423 | ------- | -0.08 | ------- | -0.46 | ------- | 1 | ------- | 0.48 |  |
|  | VAL | 424 | ------- | -2.39 | ------- | -0.98 | ------- | 1 | ------- | --- |  |
|  | ARG | 426 | ------- | -2.12 | ------- | -0.80 | ------- | 3 | ------- | 0.11 |  |
|  | ILE | 427 | ------- | -0.21 | ------- | -0.38 | ------- | 4 | ------- | 1.45 |  |
|  | LYS | 429 | ------- | -2.07 | ------- | -0.96 | ------- | 4 | ------- | 0.34 |  |
|  | VAL | 430 | Hotspot | 1.17 | ------- | -0.08 | ------- | 3 | ------- | 0.85 |  |
|  | GLU | 431 | ------- | -0.76 | ------- | -0.81 | ------- | 4 | ------- | 0.23 |  |
|  | ASP | 433 | ------- | -1.88 | ------- | -0.94 | ------- | 3 | ------- | -0.2 |  |
|  | CYS | 434 | Hotspot | 1.11 | ------- | -0.32 | ------- | 4 | ------- | -0.19 |  |
|  | LYS | 436 | ------- | --- | ------- | --- |  | 5 | ------- | -0.01 |  |
|  | MET | 437 | ------- | -0.15 | ------- | -0.25 | ------- | 5 | ------- | 0.71 |  |
|  | LYS | 438 | ------- | -1.72 | ------- | -0.93 | ------- | 3 | ------- | -0.15 |  |
|  | LEU | 440 | ------- | -1.90 | ------- | -0.97 | ------- | 6 | ------- | -0.01 |  |
|  | LYS | 441 | ------- | -0.84 | ------- | -0.42 | ------- | 4 | ------- | 0.59 |  |
|  | ALA | 444 | ------- | -0.84 | ------- | -0.77 | Conserv | 7 | ------- | --- |  |
|  | GLU | 445 | ------- | --- | ------- | --- | Conserv | 7 | ------- | -0.01 |  |
|  | ASP | 448 | ------- | -0.92 | ------- | -0.59 | ------- | 6 | ------- | -0.26 |  |
|  | LYS | 451 | Hotspot | 1.24 | Hotspot | 0.04 | Conserv | 9 | Hotspot | 3.38 | Hotspot |
|  | PHE | 454 | ------- | -0.20 | ------- | -0.33 | Conserv | 9 | ------- | 1.99 |  |
|  | LEU | 455 | Hotspot | 0.82 | Hotspot | 0.30 | Conserv | 8 | ------- | 0.74 |  |
|  | VAL | 458 | ------- | -0.19 | ------- | -0.33 | Conserv | 8 | ------- | 0.88 |  |
|  | GLU | 461 | ------- | -2.27 | ------- | -1.00 | Conserv | 9 | ------- | --- |  |
|  | ILE | 462 | Hotspot | 0.20 | ------- | -0.08 | Conserv | 8 | ------- | 1.33 |  |
|  | THR | 464 | ------- | --- | ------- | --- | Conserv | 9 | ------- | 0 |  |
|  | PRO | 465 | ------- | -1.05 | ------- | -0.83 | Conserv | 9 | ------- | --- |  |
|  | MET | 466 | Hotspot | 0.56 | Hotspot | 0.23 | Conserv | 8 | ------- | 0.63 |  |
|  | GLY | 468 | ------- | -2.41 | ------- | -0.72 | ------- | 6 | ------- | --- |  |
|  | VAL | 469 | Hotspot | 1.03 | ------- | -0.11 | Conserv | 8 | ------- | 0.69 | Hotspot |
|  | MET | 472 | ------- | -0.52 | ------- | -0.40 | ------- | 5 | ------- | 0.65 |  |
|  | LEU | 473 | Hotspot | 0.02 | Hotspot | 0.05 | ------- | 3 | ------- | 0.6 |  |
|  | MET | 475 | ------- | -1.47 | ------- | -0.91 | ------- | 5 | ------- | 0.23 |  |
|  | LEU | 476 | Hotspot | 0.86 | Hotspot | 0.11 | ------- | 6 | ------- | 1.41 |  |
|  | THR | 479 | ------- | -2.03 | ------- | -0.99 | ------- | 2 | ------- | 1.03 |  |
|  | GLU | 480 | ------- | -2.49 | ------- | -0.94 | ------- | 1 | ------- | --- |  |
|  | LEU | 481 | ------- | -2.07 | ------- | -0.84 | ------- | 1 | ------- | 0.57 |  |
|  | ASP | 482 | ------- | --- | ------- | --- |  | 1 | ------- | 1.61 |  |
|  | GLN | 485 | ------- | -0.33 | ------- | -0.27 | ------- | 4 | Hotspot | 2.37 |  |
|  | TYR | 488 | ------- | -0.58 | ------- | -0.40 | ------- | 2 | ------- | 1.69 |  |
|  | VAL | 489 | Hotspot | 0.67 | Hotspot | 0.02 | ------- | 4 | ------- | 0.24 |  |
|  | THR | 491 | ------- | -1.94 | ------- | -0.99 | ------- | 2 | ------- | 0.09 |  |
|  | ALA | 492 | Hotspot | 0.59 | ------- | -0.47 | Conserv | 7 | ------- | --- |  |
|  | SER | 495 | ------- | -0.64 | ------- | -0.80 | Conserv | 8 | ------- | -0.13 |  |
|  | GLY | 496 | ------- | -0.77 | ------- | -0.63 | ------- | 6 | ------- | --- |  |
|  | ALA | 498 | ------- | -2.72 | ------- | -0.76 | ------- | 6 | ------- | --- |  |
|  | LEU | 499 | Hotspot | 0.49 | Hotspot | 0.04 | Conserv | 8 | ------- | 1.88 | Hotspot |
|  | VAL | 500 | ------- | --- | ------- | --- |  | 4 | ------- | 0 |  |
|  | LEU | 502 | ------- | -1.33 | ------- | -0.87 | Conserv | 7 | ------- | 0.76 |  |
|  | ILE | 503 | Hotspot | 0.15 | Hotspot | 0.15 | Conserv | 8 | ------- | 1.38 | Hotspot |
|  | VAL | 506 | Hotspot | 0.34 | ------- | -0.30 | Conserv | 8 | ------- | 1.01 |  |
|  | LEU | 507 | Hotspot | 1.05 | Hotspot | 0.30 | Conserv | 9 | ------- | 0.74 |  |
|  | GLN | 509 | ------- | -1.11 | ------- | -0.83 | ------- | 5 | ------- | 0.63 |  |
|  | ALA | 510 | Hotspot | 0.28 | ------- | -0.54 | Conserv | 8 | ------- | --- |  |
|  | LYS | 511 | ------- | -1.77 | ------- | -0.90 | Conserv | 7 | ------- | --- |  |
|  | ILE | 512 | ------- | -0.23 | ------- | -0.36 | ------- | 5 | ------- | 0.81 |  |
|  | GLU | 513 | ------- | -2.70 | ------- | -0.99 | Conserv | 8 | ------- | 0.32 |  |
|  | SER | 514 | ------- | -1.09 | ------- | -0.83 | Conserv | 7 | ------- | 0.67 |  |
|  | GLY | 515 | ------- | -2.38 | ------- | -0.73 | Conserv | 9 | ------- | --- |  |

**Table S10 (beginning)**. Comparison of KFC-2 server hot spot prediction with Rosetta and PPCheck results for chains A of AHK3 HisKA domain homodimer with addition of ConSurf conservation scores.

| Chain | Residue | Number | KFC2-A | | KFC2-B | | ConSurf | | Rosetta | | PPCheck |
| --- | --- | --- | --- | --- | --- | --- | --- | --- | --- | --- | --- |
|  |  |  | Class | Confidence | Class | Confidence | Class | Value | Class | ΔΔG | Class |
| B | LEU | 414 | ------- | -1.97 | ------- | -0.98 | ------- | 6 | ------- | 0.62 |  |
|  | LEU | 415 | ------- | -1.82 | ------- | -0.86 | ------- | 5 | ------- | 0.54 |  |
|  | VAL | 416 | ------- | --- | ------- | --- | ------- | 4 | ------- | 0 |  |
|  | ALA | 417 | ------- | -2.99 | ------- | -0.89 | ------- | 1 | ------- | --- |  |
|  | ILE | 419 | ------- | -1.96 | ------- | -0.99 | ------- | 1 | ------- | 0.46 |  |
|  | ILE | 420 | ------- | -0.40 | ------- | -0.12 | ------- | 1 | ------- | 0.99 |  |
|  | THR | 423 | ------- | -0.23 | ------- | -0.59 | ------- | 1 | ------- | 0.59 |  |
|  | VAL | 424 | ------- | -2.00 | ------- | -0.97 | ------- | 1 | ------- | --- |  |
|  | ARG | 426 | ------- | -1.66 | ------- | -0.76 | ------- | 3 | ------- | 0.21 |  |
|  | ILE | 427 | ------- | -0.31 | ------- | -0.31 | ------- | 4 | ------- | 1.16 |  |
|  | LYS | 429 | ------- | -2.39 | ------- | -0.96 | ------- | 4 | ------- | 0.23 |  |
|  | VAL | 430 | Hotspot | 0.77 | ------- | -0.15 | ------- | 3 | ------- | 0.71 |  |
|  | GLU | 431 | ------- | -1.10 | ------- | -0.89 | ------- | 4 | ------- | 0.14 |  |
|  | ASP | 433 | ------- | -1.27 | ------- | -0.89 | ------- | 3 | ------- | -0.25 |  |
|  | CYS | 434 | ------- | -0.02 | ------- | -0.44 | ------- | 4 | ------- | -0.12 |  |
|  | ASP | 435 | ------- | --- | ------- | --- | ------- | 3 | ------- | -0.03 |  |
|  | MET | 437 | ------- | -0.08 | ------- | -0.30 | ------- | 5 | ------- | 0.82 |  |
|  | LYS | 438 | ------- | -1.62 | ------- | -0.95 | ------- | 3 | ------- | 0.13 |  |
|  | LEU | 440 | ------- | -2.07 | ------- | -0.96 | ------- | 6 | ------- | -0.03 |  |
|  | LYS | 441 | ------- | -0.27 | ------- | -0.32 | ------- | 4 | ------- | 0.9 | Hotspot |
|  | LYS | 443 | ------- | -2.41 | ------- | -0.96 | ------- | 3 | ------- | 0.26 |  |
|  | ALA | 444 | Hotspot | 0.58 | ------- | -0.53 | Conserv | 7 | ------- | --- |  |
|  | GLU | 445 | ------- | -2.44 | ------- | -0.93 | Conserv | 7 | ------- | -0.06 |  |
|  | ALA | 447 | ------- | -1.72 | ------- | -0.87 | Conserv | 7 | ------- | --- |  |
|  | ASP | 448 | Hotspot | 0.09 | ------- | -0.40 | ------- | 6 | Hotspot | 2.93 |  |
|  | LYS | 451 | Hotspot | 0.61 | ------- | -0.18 | Conserv | 9 | Hotspot | 2.67 |  |
|  | SER | 452 | ------- | -1.40 | ------- | -0.84 | Conserv | 7 | ------- | 0.35 |  |
|  | PHE | 454 | ------- | -1.57 | ------- | -0.86 | Conserv | 9 | ------- | 0.78 |  |
|  | LEU | 455 | Hotspot | 1.08 | Hotspot | 0.22 | Conserv | 8 | ------- | 1.32 |  |
|  | VAL | 458 | ------- | -1.44 | ------- | -0.74 | Conserv | 8 | ------- | 0.52 |  |
|  | GLU | 461 | ------- | -2.57 | ------- | -1.00 | Conserv | 9 | ------- | 0.02 |  |
|  | ILE | 462 | Hotspot | 0.84 | Hotspot | 0.06 | Conserv | 8 | ------- | 1.16 |  |
|  | PRO | 465 | ------- | -1.11 | ------- | -0.87 | Conserv | 9 | ------- | --- |  |
|  | MET | 466 | Hotspot | 0.85 | Hotspot | 0.31 | Conserv | 8 | ------- | 0.53 |  |
|  | GLY | 468 | ------- | -2.29 | ------- | -0.72 | ------- | 6 | ------- | --- |  |
|  | VAL | 469 | Hotspot | 1.21 | ------- | -0.02 | Conserv | 8 | ------- | 1.09 | Hotspot |
|  | MET | 472 | ------- | -0.51 | ------- | -0.43 | ------- | 5 | ------- | 0.75 |  |
|  | LEU | 473 | ------- | -0.30 | ------- | -0.02 | ------- | 3 | ------- | 0.18 |  |
|  | MET | 475 | ------- | -2.22 | ------- | -0.96 | ------- | 5 | ------- | 0.15 |  |
|  | LEU | 476 | Hotspot | 0.28 | ------- | 0.00 | ------- | 6 | ------- | 1.93 | Hotspot |
|  | THR | 479 | ------- | -2.21 | ------- | -0.99 | ------- | 2 | ------- | 0.61 |  |
|  | GLU | 480 | ------- | -2.39 | ------- | -0.94 | ------- | 1 | ------- | --- |  |
|  | LEU | 481 | ------- | -1.83 | ------- | -0.82 | ------- | 1 | ------- | 0.7 |  |
|  | THR | 484 | ------- | -2.78 | ------- | -0.91 | ------- | 2 | ------- | 0 |  |
|  | GLN | 485 | Hotspot | 0.26 | ------- | -0.23 | ------- | 4 | Hotspot | 2.22 |  |
|  | TYR | 488 | ------- | -0.49 | ------- | -0.44 | ------- | 2 | ------- | 1.12 |  |
|  | VAL | 489 | Hotspot | 0.42 | ------- | -0.14 | ------- | 4 | ------- | 0.25 |  |
|  | THR | 491 | ------- | -1.82 | ------- | -0.98 | ------- | 2 | ------- | -0.04 |  |
|  | ALA | 492 | Hotspot | 0.15 | ------- | -0.54 | Conserv | 7 | ------- | --- |  |
|  | SER | 495 | ------- | -0.13 | ------- | -0.57 | Conserv | 8 | ------- | -0.17 |  |
|  | GLY | 496 | ------- | -1.27 | ------- | -0.66 | ------- | 6 | ------- | --- |  |
|  | ALA | 498 | ------- | -1.90 | ------- | -0.89 | ------- | 6 | ------- | --- |  |
|  | LEU | 499 | Hotspot | 1.05 | Hotspot | 0.13 | Conserv | 8 | ------- | 1.87 | Hotspot |
|  | LEU | 502 | ------- | -0.28 | ------- | -0.51 | Conserv | 7 | ------- | 1.05 | Hotspot |
|  | ILE | 503 | Hotspot | 0.50 | Hotspot | 0.23 | Conserv | 8 | ------- | 0.67 |  |
|  | GLU | 505 | ------- | -1.62 | ------- | -0.97 | Conserv | 7 | ------- | 0.15 |  |
|  | VAL | 506 | Hotspot | 0.97 | ------- | -0.06 | Conserv | 8 | ------- | 1.06 |  |
|  | GLN | 509 | ------- | -0.30 | ------- | -0.39 | ------- | 5 | ------- | 1.12 |  |
|  | ILE | 512 | ------- | -2.82 | ------- | -0.98 | ------- | 5 | ------- | --- |  |
|  | GLU | 513 | ------- | -2.59 | ------- | -0.95 | Conserv | 8 | ------- | 0.18 |  |

**Table S10 (ending)**. Comparison of KFC-2 server hot spot prediction with Rosetta and PPCheck results for chains B of AHK3 HisKA domain homodimer with addition of ConSurf conservation scores.

|  | AHK2rd-AHP1 | | AHK2rd-AHP2 | | AHK2rd-AHP3 | | AHK3rd-AHP1 | | AHK3rd-AHP2 | | AHK3rd-AHP3 | | AHK4rd-AHP1 | | AHK4rd-AHP2 | | AHK4rd-AHP3 | | AHK5rd-AHP1 | | StHK2rd-StHP1a | | StHK3rd-StHP1a | | StHK4rd-StHP1a | |
| --- | --- | --- | --- | --- | --- | --- | --- | --- | --- | --- | --- | --- | --- | --- | --- | --- | --- | --- | --- | --- | --- | --- | --- | --- | --- | --- |
| reciever domain | mutated residue | ΔΔG of complex (kJ/mol) | mutated residue | ΔΔG of complex (kJ/mol) | mutated residue | ΔΔG of complex (kJ/mol) | mutated residue | ΔΔG of complex (kJ/mol) | mutated residue | ΔΔG of complex (kJ/mol) | mutated residue | ΔΔG of complex (kJ/mol) | mutated residue | ΔΔG of complex (kJ/mol) | mutated residue | ΔΔG of complex (kJ/mol) | mutated residue | ΔΔG of complex (kJ/mol) | mutated residue | ΔΔG of complex (kJ/mol) | mutated residue | ΔΔG of complex (kJ/mol) | mutated residue | ΔΔG of complex (kJ/mol) | mutated residue | ΔΔG of complex (kJ/mol) |
|  |  |  |  |  |  |  | D 897 | -0,14 | D 897 | -0,16 | D 897 | -0,18 |  |  | D 952 | -0,17 | D 952 | -0,1 |  |  |  |  |  |  | D 854 | -0,17 |
|  | N 1043 | 6.62 | N 1043 | 5.54 | N 1043 | 6.13 | N 898 | 2.02 | N 898 | 5.57 | N 898 | 10.07 | N 953 | 7.78 | N 953 | 8.77 | N 953 | 10.08 | N 786 | 8.99 | N 1130 | 8.82 | N 894 | 7.46 | N 855 | 5.54 |
|  | L 1044 | 0.41 | L 1044 | 0.4 | L 1044 | 0.17 | N 899 | 0 | N 899 | 1.05 | N 899 | -0.02 | I 954 | 0.22 | I 954 | 0.32 | I 954 | 0.64 | K 787 | 0.02 | N 1131 | 0.16 | N 895 | -0.01 | R 856 | 1.4 |
|  | V 1045 | 1.68 | V 1045 | 1.53 | V 1045 | 1.86 | V 900 | 1.44 | V 900 | 1.85 | V 900 | 1.91 | V 955 | 1.8 | V 955 | 1.67 | V 955 | 2.01 | I 788 | 2.49 | V 1132 | 2.05 | V 896 | 1.68 | V 857 | 1.73 |
|  | N 1046 | 5.35 | N 1046 | 1.32 | N 1046 | 4.34 | N 901 | 5.38 | N 901 | 4.96 | N 901 | 5.13 | N 956 | 5.88 | N 956 | 6 | N 956 | 6.08 | N 789 | 5.88 | N 1133 | 6.65 | N 897 | 5.11 | N 858 | 5.05 |
|  | R 1048 | 2.14 | R 1048 | 2.94 | R 1048 | 1.51 | R 903 | 0.24 | R 903 | 2.51 | R 903 | 0.91 | R 958 | 2.25 | R 958 | 1.44 | R 958 | 1.13 | M 791 | 1.28 | R 1135 | 2.26 | R 899 | 1.55 | R 860 | 1.02 |
|  | V 1049 | 1.31 | V 1049 | 1.24 | V 1049 | 1.16 | V 904 | 1.06 | V 904 | 1.06 | V 904 | 1.1 | V 959 | 1.19 | V 959 | 1.35 | V 959 | 1.24 | V 792 | 1.37 | V 1136 | 1.33 | V 900 | 1.34 | V 861 | 0.95 |
|  | E 1051 | 1.27 |  |  |  |  |  |  |  |  |  |  |  |  |  |  |  |  |  |  |  |  |  |  |  |  |
|  |  |  |  |  |  |  |  |  |  |  |  |  |  |  |  |  |  |  | S 795 | -0.28 |  |  |  |  |  |  |
|  |  |  |  |  |  |  |  |  |  |  |  |  |  |  |  |  |  |  | M 796 | 0.93 |  |  |  |  |  |  |
|  | K 1055 | 0.05 |  |  |  |  | K 910 | 0 | K 910 | 0.01 |  |  | K 965 | 0.03 | K 965 | 0.01 | K 965 | -0.01 | K 798 | -0.07 | K 1142 | 2.09 | K 906 | 0.03 | K 867 | 0.02 |
|  | K 1056 | 1.88 | K 1056 | 1.89 | K 1056 | 0.69 | K 911 | 0.29 | K 911 | 0.62 | K 911 | 1.74 | K 966 | 1 | K 966 | 0.68 | K 966 | 0.52 | Q 799 | 1.51 | K 1143 | 1.2 | K 907 | 1.96 | K 868 | 1.56 |
|  |  |  | Q 1088 | 0.09 | Q 1088 | 0.05 | Q 943 | 0.04 | Q 943 | 0.05 |  |  | Q 998 | 0.06 | Q 998 | 0.76 | Q 998 | 0.16 | С 830 | -0.09 | Q 1175 | 0.05 |  |  |  |  |
|  | D 1138 | -0.01 | D 1138 | 0.01 | D 1138 | 0.13 | D 993 | -0.01 | D 993 | 0.06 | D 993 | 0.2 | D 1036 | -0.07 | D 1036 | 0.09 | D 1036 | 0.04 | N 886 | -0.03 | D 1225 | 0.11 | D 989 | -0.06 | D 949 | -0.02 |
|  | V 1139 | 0.23 | V 1139 | 0.26 | V 1139 | 0.16 | V 994 | -0.07 | V 994 | 0.07 | V 994 | -0.01 | V 1037 | 0.22 | V 1037 | 0.24 | V 1037 | 0.21 | T 887 | 0.11 | V 1226 | -0.03 | V 990 | -0.03 | V 950 | 0.15 |
|  | Q 1141 | 1.25 |  |  |  |  |  |  |  |  | Q 996 | 0.22 |  |  |  |  |  |  |  |  |  |  |  |  |  |  |
|  |  |  |  |  |  |  |  |  |  |  |  |  |  |  |  |  |  |  | E 890 | -0.19 |  |  |  |  |  |  |
|  | K 1158 | 2.92 | K 1158 | 3.73 | K 1158 | 6.69 | K 1013 | 4.06 | K 1013 | 4.59 | K 1013 | 0.11 | K 1056 | 5.79 | K 1056 | 8.03 | K 1056 | 5.93 | K 906 | 2.07 | K 1245 | 4.87 | K 1009 | 6.04 | K 969 | 5.39 |
|  | F 1160 | 0.06 | F 1160 | 0.01 | F 1160 | 0 | F 1015 | 0.11 | F 1015 | 0.07 | F 1015 | 0.02 | F 1058 | 0.08 | F 1058 | 0.25 | F 1058 | 0.13 | V 908 | 0.01 | F 1247 | 0.04 | F 1011 | 0.2 | F 971 | 0.04 |
|  | E 1161 | -0.07 | E 1161 | -0.09 | E 1161 | 0 | E 1016 | -0.07 | E 1016 | 0.01 | E 1016 | 0.03 | E 1059 | 1.96 | E 1059 | 0.05 | E 1059 | 0.01 | T 909 | 0.09 | E 1248 | -0.03 |  |  | E 972 | 0.52 |
|  | E 1162 | 2.01 | E 1162 | 0.29 | E 1162 | 0.36 |  |  |  |  |  |  | E 1060 | -0.09 | E 1060 | 0.02 | E 1060 | 0.33 | L 910 | 0.33 | K 1249 | 1.51 |  |  | E 973 | 0.09 |
|  |  |  |  |  |  |  | E 1018 | -0.02 |  |  |  |  |  |  |  |  | E 1061 | 0.66 |  |  |  |  |  |  |  |  |
| phospho-transmitter | mutated residue | ΔΔG of complex (kJ/mol) | mutated residue | ΔΔG of complex (kJ/mol) | mutated residue | ΔΔG of complex (kJ/mol) | mutated residue | ΔΔG of complex (kJ/mol) | mutated residue | ΔΔG of complex (kJ/mol) | mutated residue | ΔΔG of complex (kJ/mol) | mutated residue | ΔΔG of complex (kJ/mol) | mutated residue | ΔΔG of complex (kJ/mol) | mutated residue | ΔΔG of complex (kJ/mol) | mutated residue | ΔΔG of complex (kJ/mol) | mutated residue | ΔΔG of complex (kJ/mol) | mutated residue | ΔΔG of complex (kJ/mol) | mutated residue | ΔΔG of complex (kJ/mol) |
|  | Q 26 | 1.93 | Q 28 | 1.64 | Q 28 | 1.67 | Q 26 | 2.06 | Q 28 | 1.79 | Q 28 | 1.95 | Q 26 | 1.85 | Q 28 | 1.56 | Q 28 | 1.87 | Q 26 | 1.77 | Q 26 | 1.85 | Q 26 | 1.83 | Q 26 | 1.82 |
|  | Q 29 | 1.61 | E 31 | 1.97 | E 31 | 0.77 | Q 29 | 1.62 | E 31 | 1 | E 31 | 1.07 | Q 29 | 3.47 | E 31 | 0.06 | E 31 | 0.81 | Q 29 | 1.68 | Q 29 | 0.67 | Q 29 | 0.21 | Q 29 | 2.13 |
|  | L 30 | 0.43 | L 32 | 0.39 | L 32 | 0.43 | L 30 | 0.33 | L 32 | 0.42 | L 32 | 0.33 | L 30 | 0.32 | L 32 | 0.36 | L 32 | 0.45 | L 30 | 0.49 | L 30 | 0.42 | L 30 | 0.42 | L 30 | 0.32 |
|  | Q 32 | 2.02 | K 34 | 1.31 | K 34 | 0.31 | Q 32 | 0.01 | K 34 | -0.26 | K 34 | -0.27 |  |  | K 34 | -0.1 | K 34 | 1.27 | Q 32 | 0.41 | Q 32 | 0.06 | Q 32 | 0.01 | Q 32 | 0.11 |
|  | L 33 | 1.92 | L 35 | 1.75 | L 35 | 1.91 | L 33 | 1.69 | L 35 | 1.68 | L 35 | 1.53 | L 33 | 1.6 | L 35 | 1.57 | L 35 | 1.61 | L 33 | 2.03 | L 33 | 2.13 | L 33 | 1.95 | L 33 | 1.62 |
|  | D 35 | 0.46 | D 37 | 0.38 | D 37 | 0.01 | D 35 | -0.05 | D 37 | 0.12 | D 37 | 0.42 | D 35 | 0.2 | D 37 | 0.51 | D 37 | 0.27 |  |  | D 35 | 0.39 | D 35 | 0.28 | D 35 | 0.22 |
|  | S 37 | -0.02 |  |  |  |  |  |  |  |  |  |  |  |  |  |  |  |  |  |  | S 37 | 0.43 | S 37 | -0.01 |  |  |
|  | N 38 | 0.01 | S 40 | 0.36 | S 40 | -0.01 |  |  | S 40 | -0.01 | S 40 | 0.65 |  |  | S 40 | -0.02 | S 40 | 0 | N 38 | 0.03 | N 38 | 0.76 |  |  | N 38 | 0 |
|  | F 41 | 1.25 | F 43 | 1.1 | F 43 | 1.11 | F 41 | 1.17 | F 43 | 1.46 | F 43 | 1.15 | F 41 | 1.2 | F 43 | 1.24 | F 43 | 1.19 | F 41 | 1.59 | F 41 | 1.52 | F 41 | 1.29 | F 41 | 1.22 |
|  | Q 44 | 0.57 | E 46 | 1.31 | E 46 | 1.11 |  |  | E 46 | 1.42 | E 46 | 0.32 | Q 44 | 0.72 | E 46 | 0.9 | E 46 | 0.53 | Q 44 | 0.08 | E 44 | 1.07 | E 44 | 1.04 | E 44 | 0.54 |
|  | V 45 | 0.72 | V 47 | 0.76 | V 47 | 0.78 | V 45 | 0.84 | V 47 | 0.78 | V 47 | 0.8 | V 45 | 0.8 | V 47 | 0.85 | V 47 | 0.55 | V 45 | 0.94 | V 45 | 0.78 | V 45 | 0.67 | V 45 | 0.67 |
|  | L 48 | 1.58 | L 50 | 1.53 | L 50 | 1.38 | L 48 | 1.29 | L 50 | 0.43 | L 50 | 1.3 | L 48 | 1.49 | L 50 | 1.11 | L 50 | 1.89 | L 48 | 0.92 | L 48 | 1.58 | L 48 | 1.06 | L 48 | 1.17 |
|  | F 49 | 0.11 | F 51 | 0.14 |  |  | F 49 | 0.12 | F 51 | 0.17 |  |  | F 49 | 0.11 |  |  | F 51 | 0.13 | F 49 | 0.18 | F 49 | 0.14 | F 49 | 0.13 | F 49 | 0.13 |
|  |  |  |  |  |  |  |  |  |  |  |  |  |  |  |  |  |  |  |  |  |  |  |  |  | E 51 | 1.33 |
|  |  |  | D 54 | 2.99 | D 54 | 2.75 | D 52 | -0.07 | D 54 | 3.55 | D 54 | 1.52 | D 52 | 1.57 |  |  | D 54 | 1.44 |  |  | D 52 | 1.46 |  |  |  |  |
|  |  |  |  |  |  |  |  |  |  |  | S 79 | 0 |  |  |  |  | S 79 | 0 |  |  |  |  |  |  |  |  |
|  | H 79 | 0.54 | H 82 | 1.82 | H 82 | 0.53 | H 79 | 2.05 | H 82 | 0.39 | H 82 | 1.61 | H 79 | 0.9 | H 82 | 0.66 | H 82 | 1.32 | H 79 | 0.69 | H 79 | 2.21 | H 79 | 1.62 | H 79 | 1.32 |
|  | Q 80 | 2.81 | Q 83 | 2.58 | Q 83 | 3.41 | Q 80 | 3.15 | Q 83 | 2.9 | Q 83 | 3.33 | Q 80 | 3.44 | Q 83 | 1.34 | Q 83 | 3.33 | Q 80 | 2.95 | Q 80 | 3.18 | Q 80 | 3.36 | Q 80 | 2.81 |
|  | K 82 | 1.75 | K 85 | 1.74 | K 85 | 2.06 | K 82 | 1.52 | K 85 | 1.73 | K 85 | 1.24 | K 82 | 1.22 | K 85 | 0.67 | K 85 | 1.83 | K 82 | 1.41 | K 82 | 1.62 | K 82 | 1.46 | K 82 | 0.99 |
|  | S 84 | 2.43 | S 87 | 5.71 | S 87 | 5.28 | S 84 | 2.33 | S 87 | 5.25 | S 87 | 5.23 | S 84 | 5.3 | S 87 | 5.92 | S 87 | 5.56 | S 84 | 5.54 | S 84 | 5.32 | S 84 | 5.68 | S 84 | 3.43 |
|  | S 86 | -0.07 | S 89 | 0.03 | S 89 | -0.11 | S 86 | -0.01 | S 89 | 0.49 | S 89 | -0.05 | S 86 | -0.05 | S 89 | -0.1 | S 89 | 2.85 | S 86 | 0.06 | S 86 | -0.09 | S 86 | -0.13 | S 86 | 0.91 |
|  | S 87 | 1.83 | S 90 | -0.18 | S 90 | 0.99 | S 87 | 1.87 | S 90 | 1.7 | S 90 | 2.14 | S 87 | 1.31 | S 90 | -0.18 | S 90 | 6.31 | S 87 | 2 | S 87 | 1.94 | S 87 | -0.18 | S 87 | 2.7 |
|  | I 88 | 0.34 | V 91 | 0.07 | V 91 | 0.3 | I 88 | 0.33 | V 91 | 0.22 | V 91 | 0.18 | I 88 | 0.34 | V 91 | 0.22 | V 91 | 0.09 | I 88 | 0.26 | V 88 | 0.06 | V 88 | 0.07 | V 88 | 0.21 |
|  | R 101 | 1.15 |  |  |  |  |  |  |  |  | K104 | 1.15 | R 101 | 0.43 |  |  |  |  | R 101 | 0.25 |  |  |  |  |  |  |
|  |  |  |  |  |  |  |  |  |  |  |  |  |  |  | N 156 | 0.58 |  |  |  |  |  |  |  |  |  |  |
|  |  |  |  |  |  |  | F 154 | 0.7 |  |  |  |  |  |  |  |  |  |  |  |  |  |  |  |  |  |  |

**Table S11**. Virtual alanine scanning for modeled HK(rd) –HPt complexes. Hot spots with ΔΔG > 4 kJ/mol colored yellow, hot spots with ΔΔG between 2 and 4 kJ/mol colored red.

| Chain | Residue | Number | KFC2-A | | KFC2-B | | ConSurf | | Rosetta | | PPCheck |
| --- | --- | --- | --- | --- | --- | --- | --- | --- | --- | --- | --- |
|  |  |  | Class | Confidence | Class | Confidence | Class | Value | Class | DDG | Class |
| A | ASP | 897 | ------- | -2.50 | ------- | -0.99 | Conserv | 9 | ------- | -0.16 |  |
|  | ASN | 898 | Hotspot | 0.31 | ------- | -0.17 | Conserv | 7 | Hotspot | 5.57 | Hotspot |
|  | ASN | 899 | ------- | -0.98 | ------- | -0.78 | ------- | 1 | ------- | 1.05 |  |
|  | VAL | 900 | Hotspot | 1.26 | ------- | -0.12 | ------- | 4 | ------- | 1.85 | Hotspot |
|  | ASN | 901 | Hotspot | 0.71 | Hotspot | 0.03 | Conserv | 8 | Hotspot | 4.96 | Hotspot |
|  | ARG | 903 | Hotspot | 0.84 | Hotspot | 0.03 | ------- | 3 | Hotspot | 2.51 | Hotspot |
|  | VAL | 904 | Hotspot | 1.55 | Hotspot | 0.06 | ------- | 5 | ------- | 1.06 | Hotspot |
|  | GLY | 907 | ------- | -0.77 | ------- | -0.63 | ------- | 1 | ------- | --- |  |
|  | ALA | 908 | ------- | -0.55 | ------- | -0.65 | ------- | 4 | ------- | --- |  |
|  | LYS | 910 | ------- | --- | ------- | --- | ------- | 3 | ------- | 0.01 |  |
|  | LYS | 911 | ------- | -1.35 | ------- | -0.81 | ------- | 4 | ------- | 0.62 |  |
|  | ASP | 941 | ------- | -2.62 | ------- | -0.95 | Conserv | 9 | ------- | --- |  |
|  | GLN | 943 | ------- | -2.33 | ------- | -0.93 | Conserv | 8 | ------- | 0.05 |  |
|  | ALA | 992 | ------- | -1.17 | ------- | -0.79 | Conserv | 9 | ------- | --- |  |
|  | ASP | 993 | ------- | -2.66 | ------- | -0.95 | ------- | 5 | ------- | 0.06 |  |
|  | VAL | 994 | ------- | -2.14 | ------- | -0.85 | Conserv | 7 | ------- | 0.07 |  |
|  | LYS | 1013 | ------- | -1.47 | ------- | -0.69 | Conserv | 9 | Hotspot | 4.59 |  |
|  | PRO | 1014 | ------- | -0.46 | ------- | -0.85 | Conserv | 9 | ------- | --- | Hotspot |
|  | PHE | 1015 | ------- | -0.50 | ------- | -0.28 | ------- | 5 | ------- | 0.07 |  |
|  | GLU | 1016 | ------- | -2.15 | ------- | -0.99 | ------- | 2 | ------- | 0.01 |  |
|  | ALA | 1017 | ------- | -0.64 | ------- | -0.71 | ------- | 2 | ------- | --- |  |
|  | TYR | 1021 | ------- | -3.17 | ------- | -0.80 | ------- | 3 | ------- | --- |  |
|  | | | | | | | | | | | |
| Chain | Residue | Number | KFC2-A | | KFC2-B | | ConSurf | | Rosetta | | PPCheck |
|  |  |  | Class | Confidence | Class | Confidence | Class | Value | Class | DDG | Class |
| B | GLN | 28 | ------- | -0.09 | ------- | -0.13 | Conserv | 9 | ------- | 1.79 |  |
|  | GLU | 31 | ------- | -0.82 | ------- | -0.30 | Conserv | 7 | ------- | 1 | Hotspot |
|  | LEU | 32 | Hotspot | 0.70 | Hotspot | 0.31 | Conserv | 8 | ------- | 0.42 |  |
|  | LYS | 34 | ------- | -2.41 | ------- | -0.96 | ------- | 4 | ------- | -0.26 |  |
|  | LEU | 35 | Hotspot | 0.60 | Hotspot | 0.09 | Conserv | 7 | ------- | 1.68 | Hotspot |
|  | ASP | 37 | ------- | -2.09 | ------- | -0.99 | ------- | 6 | ------- | 0.12 |  |
|  | SER | 40 | ------- | -2.78 | ------- | -0.88 | ------- | 4 | ------- | -0.01 |  |
|  | PHE | 43 | ------- | -0.32 | Hotspot | 0.01 | ------- | 4 | ------- | 1.46 |  |
|  | GLU | 46 | ------- | -2.21 | ------- | -1.00 | Conserv | 7 | ------- | 1.42 |  |
|  | VAL | 47 | Hotspot | 0.32 | ------- | -0.04 | Conserv | 7 | ------- | 0.78 |  |
|  | LEU | 50 | ------- | -1.10 | ------- | -0.76 | ------- | 6 | ------- | 0.43 |  |
|  | PHE | 51 | Hotspot | 0.28 | Hotspot | 0.32 | Conserv | 9 | ------- | 0.17 |  |
|  | ASP | 54 | ------- | -1.19 | ------- | -0.79 | Conserv | 9 | Hotspot | 3.55 |  |
|  | HIS | 82 | ------- | -1.16 | ------- | -0.70 | Conserv | 9 | ------- | 0.39 |  |
|  | GLN | 83 | ------- | -0.91 | ------- | -0.64 | Conserv | 9 | Hotspot | 2.9 |  |
|  | LYS | 85 | ------- | -0.01 | ------- | -0.04 | Conserv | 9 | ------- | 1.73 |  |
|  | GLY | 86 | ------- | -1.18 | ------- | -0.69 | Conserv | 9 | ------- | --- |  |
|  | SER | 87 | ------- | -0.33 | ------- | -0.58 | Conserv | 8 | Hotspot | 5.25 |  |
|  | SER | 89 | ------- | -0.82 | ------- | -0.72 | Conserv | 7 | ------- | 0.49 |  |
|  | SER | 90 | Hotspot | 1.02 | ------- | -0.37 | Conserv | 8 | ------- | 1.7 | Hotspot |
|  | VAL | 91 | Hotspot | 0.77 | ------- | -0.03 | Conserv | 8 | ------- | 0.22 |  |
|  | LYS | 97 | ------- | -2.33 | ------- | -0.95 | ------- | 6 | ------- | --- |  |
|  | VAL | 101 | ------- | -3.24 | ------- | -0.95 | Conserv | 7 | ------- | --- |  |
|  | LYS | 104 | ------- | -2.17 | ------- | -0.75 | ------- | 6 | ------- | --- |  |
|  | GLU | 108 | ------- | -3.37 | ------- | -0.86 | ------- | 6 | ------- | --- |  |

**Table S12**. Comparison of KFC-2 server hot spot prediction with Rosetta and PPCheck results for AHK3rd–AHP2 complex with addition of ConSurf conservation scores.

| Chain A - AHK3rd | HSDC | ASA | BSA | % of BSA | ΔiG |
| --- | --- | --- | --- | --- | --- |
| A:ASP 897 |  | 75.76 | 9.97 | 13 | -0.05 |
| A:ASN 898 | H | 55.45 | 52.42 | 95 | -0.47 |
| A:ASN 899 | H | 111.43 | 53.70 | 48 | -0.56 |
| A:VAL 900 |  | 94.98 | 94.81 | 100 | 1.52 |
| A:ASN 901 | H | 38.87 | 37.79 | 97 | -0.47 |
| A:ARG 903 | HS | 189.61 | 134.28 | 71 | -1.12 |
| A:VAL 904 |  | 80.70 | 77.47 | 96 | 1.17 |
| A:GLY 907 |  | 42.99 | 25.34 | 59 | 0.38 |
| A:ALA 908 |  | 24.67 | 19.67 | 80 | 0.24 |
| A:LYS 911 | HS | 167.62 | 72.26 | 43 | 0.17 |
| A:ASP 941 |  | 8.14 | 0.73 | 9 | -0.01 |
| A:GLN 943 |  | 68.73 | 19.53 | 28 | 0.31 |
| A:ALA 992 | H | 48.30 | 34.56 | 72 | 0.11 |
| A:ASP 993 | HS | 135.28 | 30.69 | 23 | -0.29 |
| A:VAL 994 |  | 83.58 | 40.76 | 49 | 0.65 |
| A:LYS1013 |  | 28.14 | 11.59 | 41 | 0.05 |
| A:PRO1014 |  | 101.23 | 51.41 | 51 | 0.81 |
| A:PHE1015 | H | 46.46 | 25.81 | 56 | -0.21 |
| A:GLU1016 |  | 88.02 | 20.07 | 23 | 0.30 |
| A:ALA1017 | H | 49.19 | 39.22 | 80 | 0.48 |
| A:TYR1021 |  | 118.12 | 0.78 | 1 | 0.01 |
|  | | | | | |
| Chain B - AHP2 | HSDC | ASA | BSA | % of BSA | ΔiG |
| B:GLN  28 | H | 78.31 | 63.73 | 81 | -0.68 |
| B:GLU  31 | H | 59.33 | 45.81 | 77 | 0.05 |
| B:LEU  32 |  | 11.72 | 11.72 | 100 | 0.19 |
| B:LYS  34 |  | 101.2 | 21.86 | 22 | -0.61 |
| B:LEU  35 |  | 87.14 | 80.31 | 92 | 1.09 |
| B:ASP  37 | HS | 81.03 | 28.57 | 35 | -0.33 |
| B:SER  40 |  | 35.28 | 0.49 | 1 | -0.01 |
| B:PHE  43 |  | 57.31 | 47.12 | 82 | 0.75 |
| B:GLU  46 | HS | 104.70 | 27.57 | 26 | -0.21 |
| B:VAL  47 |  | 36.30 | 35.96 | 99 | 0.53 |
| B:LEU  50 |  | 127.58 | 53.05 | 42 | 0.85 |
| B:PHE  51 |  | 7.13 | 7.13 | 100 | 0.07 |
| B:ASP  54 | H | 39.64 | 24.46 | 62 | 0.17 |
| B:HIS  82 |  | 106.65 | 57.20 | 54 | -0.28 |
| B:GLN  83 | H | 82.60 | 48.59 | 59 | -0.61 |
| B:LYS  85 | HS | 67.72 | 57.55 | 85 | -1.43 |
| B:GLY  86 | H | 43.86 | 25.04 | 57 | 0.31 |
| B:SER  87 | H | 23.02 | 19.93 | 87 | 0.13 |
| B:SER  89 |  | 42.18 | 29.88 | 71 | -0.10 |
| B:SER  90 | H | 58.60 | 58.12 | 99 | 0.55 |
| B:VAL  91 |  | 2.18 | 2.18 | 100 | 0.03 |
| B:LYS  97 |  | 39.23 | 5.08 | 13 | -0.17 |
| B:VAL 101 |  | 64.56 | 4.19 | 6 | 0.07 |
| B:LYS 104 |  | 49.41 | 23.49 | 48 | 0.09 |
| B:GLU 108 |  | 112.99 | 4.26 | 4 | -0.04 |

**Table S13**. Involvement in Hydrogen bond, Salt bridge, Disulphide bond, or Covalent link (HSDC), ASA - Accessible Surface Area (Å²), BSA - Buried Surface Area (Å²), Buried area percentage (% of BSA) and ΔiG - Solvation energy effect (kcal/mol) of interface residues of AHK3rd–AHP2 complex.

|  | AHP1-AHP1 | | AHP1-AHP2 | | AHP1-AHP3 | | AHP2-AHP2 | | AHP2-AHP3 | | AHP3-AHP3 | | StHP1a-StHP1a | |
| --- | --- | --- | --- | --- | --- | --- | --- | --- | --- | --- | --- | --- | --- | --- |
|  | mutated residue | ΔΔG of complex (kJ/mol) | mutated residue | ΔΔG of complex (kJ/mol) | mutated residue | ΔΔG of complex (kJ/mol) | mutated residue | ΔΔG of complex (kJ/mol) | mutated residue | ΔΔG of complex (kJ/mol) | mutated residue | ΔΔG of complex (kJ/mol) | mutated residue | ΔΔG of complex (kJ/mol) |
| CHAIN A |  |  |  |  |  |  | E 31 | 0.22 |  |  |  |  |  |  |
|  | L 30 | 0.14 | L 30 | 0.15 | L 30 | 0.21 | L 32 | 0.17 | L 32 | 0.21 | L 32 | 0.15 | L 30 | 0.17 |
|  | L 33 | 0.24 | L 33 | -0.01 | L 33 | 0.12 | L 35 | 0.2 | L 35 | 0.32 | L 35 | 0.46 | L 33 | 0.13 |
|  | E 36 | 1.75 | E 36 | 0.1 | E 36 | 0.24 | D 38 | 0.23 | D 38 | 1.15 | E 38 | 1.94 | D36 | -0.07 |
|  | S 37 | 0.15 | S 37 | 0.04 | S 37 | 0.78 |  |  |  |  | C 39 | -0.17 | S37 | 0.82 |
|  | N 38 | 3.73 | N 38 | 0.9 | N 38 | 0 | S 40 | -0.14 | S 40 | -0.1 | S 40 | 0 | N 38 | 0.71 |
|  | D 40 | -0.23 | D 40 | 0.14 | D 40 | -0.18 | D 42 | -0.13 | D 42 | -0.21 | D 42 | 1.9 | D 40 | 1.39 |
|  | F 41 | 1.8 | F 41 | 1.96 | F 41 | 1.62 | F 43 | 1.86 | F 43 | 1.92 | F 43 | 1.73 | F 41 | 2.13 |
|  |  |  |  |  | 43 | 0 | S 45 | 0 |  |  |  |  |  |  |
|  | Q 44 | 1.8 | Q 44 | 1.13 | Q 44 | 1.02 | E 46 | 0.05 | E 46 | 0.58 | E 46 | 1.69 | E 44 | 2.58 |
|  | V 45 | 0.55 | V 45 | 0.6 | V 45 | 0.58 | V 47 | 0.58 | V 47 | 0.51 | V 47 | 0.7 | V 45 | 0.61 |
|  |  |  | T 47 | 0 |  |  | S 49 | 0 |  |  | T 49 | 0.11 | S 47 | 0.55 |
|  | L 48 | 1.8 | L 48 | 1.27 | L 48 | 1.11 | L 50 | 1.7 | L 50 | 1.51 | L 50 | 1.49 | L 48 | 1.76 |
|  | F 49 | 0 | F 49 | 0 |  |  | F 51 | 0 |  |  |  |  |  |  |
|  | D 52 | 4.38 | D 52 | 4.23 | D 52 | 3.6 | D 54 | 5.63 | D 54 | 3.53 | D 54 | 4.42 | D 52 | 0.77 |
|  | R 55 | 0.55 | R 55 | 0.16 | R 55 | 1.37 |  |  | K 57 | -0.33 | K 57 | 0.5 |  |  |
|  |  |  |  |  |  |  |  |  | D 73 | -0.17 |  |  | D 70 | -0.2 |
|  | F 71 | 0 | F 71 | 0 | F 71 | 0 | F 74 | 0.01 | F 74 | 0 | F 74 | 0.01 | F 71 | 0.02 |
|  | K 72 | 1.14 | K 72 | 0.37 | K 72 | 1.32 | S 75 | -0.25 | S 75 | -0.3 | K 75 | -0.3 | K 72 | 1.03 |
|  | K 73 | 0.3 | K 73 | 0.63 | K 73 | 2.87 | Q 76 | 2.37 | Q 76 | 0.23 | L 76 | 0.95 | Q 73 | 0.69 |
|  |  |  |  |  | V 74 | 0 | V 77 | 0 |  |  |  |  | V 74 | 0 |
|  | D 75 | 1.94 | D 75 | 1.9 | D 75 | 4.28 |  |  |  |  |  |  | D 75 | 0.81 |
|  |  |  |  |  |  |  |  |  |  |  | S 79 | -0.18 |  |  |
|  | H 77 | 0.37 | H 77 | 0.27 | H 77 | 0.28 | S 80 | -0.01 | S 80 | -0.08 | S 80 | 0 | H 77 | 0.36 |
|  | H 79 | 1 | H 79 | 1.36 | H 79 | 1.28 | H 82 | 1.52 | H 82 | 0.96 | H 82 | 1.05 | H 79 | 1.05 |
|  | Q 80 | 4.42 | Q 80 | 0.35 | Q 80 | 5.79 | Q 83 | 3.09 | Q 83 | 4.26 | Q 83 | 4.4 | Q 80 | 1.69 |
|  | S 84 | -0.01 | S 84 | -0.02 | S 84 | -0.02 | S 87 | 2.63 | S 87 | -0.01 | S 87 | -0.01 | S 84 | 0.31 |
|  | S 86 | -0.01 | S 86 | -0.01 | S 86 | 0 |  |  | S 89 | -0.02 |  |  |  |  |
|  | S 87 | -0.05 | S 87 | -0.05 | S 87 | -0.02 | S 90 | -0.03 | S 90 | -0.08 | S 90 | -0.01 | S 87 | -0.04 |
|  | I 88 | 0 |  |  | I 88 | 0.07 | V 91 | 0.03 | V 91 | 0.05 |  |  | V 88 | 0 |
|  | R 101 | 3.89 | R 101 | 4.63 | R 101 | 6.46 | K 104 | 1.51 | K 104 | 1.28 | K 104 | 1.12 | R 101 | 3.11 |
|  | C 104 | -0.04 |  |  | C 104 | -0.03 | C 107 | -0.01 | C 107 | -0.01 |  |  | C 104 | -0.04 |
|  | E 105 | 1.62 | E 105 | 0.04 |  |  | E 108 | 0.65 | E 108 | 0.08 | D 108 | 1.95 | E 105 | 1.09 |
|  |  |  |  |  |  |  |  |  |  |  |  |  | Q 107 | 0.45 |
|  | | | | | | | | | | | | | | |
|  | AHP1-AHP1 | | AHP1-AHP2 | | AHP1-AHP3 | | AHP2-AHP2 | | AHP2-AHP3 | | AHP3-AHP3 | | StHP1a-StHP1a | |
|  | mutated residue | ΔΔG of complex (kJ/mol) | mutated residue | ΔΔG of complex (kJ/mol) | mutated residue | ΔΔG of complex (kJ/mol) | mutated residue | ΔΔG of complex (kJ/mol) | mutated residue | ΔΔG of complex (kJ/mol) | mutated residue | ΔΔG of complex (kJ/mol) | mutated residue | ΔΔG of complex (kJ/mol) |
| CHAIN B | L 30 | 0.18 | L 32 | 0.21 | L 32 | 0.2 | L 32 | 0.22 |  |  | L 32 | 0.22 |  |  |
|  | L 33 | 0.13 | L 35 | 0.31 | L 35 | 0.17 | L 35 | 0.25 | L 35 | 0.06 | L 35 | 0.1 | L 33 | 0.24 |
|  | E 36 | 0.5 | D 38 | 0.45 | E 38 | 0.17 | D 38 | -0.14 | E 38 | 0.33 |  |  | D36 | 1.44 |
|  | S 37 | -0.1 |  |  | C 39 | -0.29 |  |  | C 39 | -0.13 | C 39 | -0.27 | S37 | -0.23 |
|  | N 38 | 0.81 | S 40 | -0.02 | S 40 | 1.56 | S 40 | -0.08 | S 40 | -0.07 | S 40 | -0.02 | N 38 | 0.99 |
|  | D 40 | -0.17 | D 42 | -0.14 | D 42 | 0.54 | D 42 | 0.07 | D 42 | -0.29 | D 42 | 0.18 | D 40 | 0.77 |
|  | F 41 | 1.82 | F 43 | 1.87 | F 43 | 1.67 | F 43 | 1.78 | F 43 | 1.86 | F 43 | 1.95 | F 41 | 2.37 |
|  |  |  |  |  |  |  |  |  | V 44 | 0 |  |  |  |  |
|  |  |  | S 45 | 0 |  |  | S 45 | 0 |  |  |  |  |  |  |
|  | Q 44 | 0.43 | E 46 | 0.73 | E 46 | 3 | E 46 | 2.03 | E 46 | 0.02 | E 46 | 0.1 | E 44 | 0.64 |
|  | V 45 | 0.59 | V 47 | 0.81 | V 47 | 0.71 | V 47 | 0.81 | V 47 | 0.61 | V 47 | 0.63 | V 45 | 0.64 |
|  | L 48 | 1.14 | L 50 | 1.15 | L 50 | 1.54 | L 50 | 1.51 | L 50 | 1.47 | L 50 | 1.61 | L 48 | 1.56 |
|  |  |  |  |  |  |  | F 51 | 0.02 |  |  |  |  | F 49 | 0.02 |
|  | D 52 | 2.24 | D 54 | 1.49 | D 54 | 4.84 | D 54 | 1.96 | D 54 | 2.87 | D 54 | 4.33 | D 52 | 0.77 |
|  | R 55 | 0 | K 57 | 0.13 | K 57 | -0.18 |  |  | K 57 | 0.56 | K 57 | 0.53 |  |  |
|  |  |  |  |  |  |  |  |  |  |  | L 58 | 0.11 |  |  |
|  | D 70 | 0.8 | D 73 | 0.25 | D 73 | 0.13 |  |  |  |  | D 73 | -0.03 | D 70 | 0.42 |
|  | F 71 | 0.09 | F 74 | 0.03 | F 74 | 0.01 | F 74 | 0.01 | F 74 | 0.01 | F 74 | 0.01 | F 71 | 0.04 |
|  | K 72 | 1.38 | S 75 | 0.12 | K 75 | 2.2 | S 75 | -0.22 | K 75 | 0.78 | K 75 | 3.84 | K 72 | 2.18 |
|  | K 73 | 0.05 | Q 76 | 0.14 | L 76 | 0.38 | Q 76 | 0.79 | L 76 | 0.52 | L 76 | 0.66 | Q 73 | 1.95 |
|  | V 74 | -0.01 |  |  |  |  | V 77 | 0 |  |  |  |  |  |  |
|  | D 75 | 7.03 |  |  |  |  |  |  |  |  |  |  | D 75 | -0.16 |
|  |  |  |  |  | S 79 | -0.31 |  |  | S 79 | -0.39 | S 79 | -0.3 |  |  |
|  | H 77 | 0.45 |  |  | S 80 | -0.06 | S 80 | 0.28 |  |  | S 80 | -0.04 | H 77 | 0.63 |
|  | H 79 | 1.08 | H 82 | 1.01 | H 82 | 1.36 | H 82 | 1.11 | H 82 | 1.19 | H 82 | 1.37 | H 79 | 1.03 |
|  | Q 80 | 2.82 | Q 83 | 5.54 | Q 83 | 4.48 | Q 83 | 3.47 | Q 83 | 3.08 | Q 83 | 4.45 | Q 80 | 1 |
|  |  |  |  |  |  |  | K 85 | 0.05 |  |  |  |  |  |  |
|  | S 84 | -0.04 | S 87 | -0.01 | S 87 | 1.31 | S 87 | -0.03 | S 87 | -0.03 | S 87 | -0.01 | S 84 | 0.92 |
|  | S 86 | 0 |  |  | S 89 | 0 |  |  |  |  | S 89 | 0 |  |  |
|  | S 87 | -0.02 | S 90 | -0.02 | S 90 | -0.06 | S 90 | -0.05 | S 90 | -0.03 | S 90 | -0.04 | S 87 | -0.02 |
|  | I 88 | 0.05 |  |  | V 91 | 0.07 |  |  |  |  |  |  | V 88 | 0 |
|  | R 101 | 8.05 | K 104 | 1.65 | K 104 | 0.06 | K 104 | 1.03 | K 104 | 1.28 | K 104 | 2.12 | R 101 | 0.84 |
|  | C 104 | -0.06 | C 107 | -0.06 | C 107 | -0.07 | C 107 | -0.02 | C 107 | -0.02 | C 107 | -0.03 | C 104 | -0.02 |
|  | E 105 | 0.06 | E 108 | 0.58 | D 108 | 0.88 | E 108 | 0.13 | D 108 | 0.99 | D 108 | 0.81 | E 105 | 0.04 |
|  | Q 107 | 0.24 | K 110 | 0.58 |  |  | K 110 | 0.72 | Q 110 | 1.16 | Q 110 | 0.64 |  |  |

**Table S14**. Virtual alanine scanning for modeled HPt–HPt complexes. “Hot spots with ΔΔG > 4 kJ/mol colored yellow, hot spots with ΔΔG between 2 and 4 kJ/mol colored red.**Table S15**. Comparison of KFC-2 server hot spot prediction with Rosetta and PPCheck results for AHP2 homodimer with addition of ConSurf conservation scores.

| Chain | Residue | Number | KFC2-A | | KFC2-B | | ConSurf | | Rosetta | | PPCheck |
| --- | --- | --- | --- | --- | --- | --- | --- | --- | --- | --- | --- |
|  |  |  | Class | Confidence | Class | Confidence | Class | Value | Class | DDG | Class |
| A | GLU | 31 | ------- | -2.37 | ------- | -1.00 | Conserv | 7 | ------- | 0.22 |  |
|  | LEU | 32 | ------- | -0.15 | Hotspot | 0.10 | Conserv | 8 | ------- | 0.17 |  |
|  | LEU | 35 | ------- | -1.32 | ------- | -0.75 | Conserv | 7 | ------- | 0.2 |  |
|  | ASP | 38 | ------- | -1.11 | ------- | -0.79 | ------- | 6 | ------- | 0.23 |  |
|  | GLY | 39 | ------- | -0.46 | ------- | -0.65 | ------- | 3 | ------- | --- |  |
|  | SER | 40 | ------- | -1.04 | ------- | -0.83 | ------- | 4 | ------- | -0.14 |  |
|  | ASP | 42 | ------- | -1.63 | ------- | -0.97 | ------- | 4 | ------- | -0.13 |  |
|  | PHE | 43 | Hotspot | 1.34 | Hotspot | 0.34 | ------- | 4 | ------- | 1.86 | Hotspot |
|  | SER | 45 | ------- | --- | ------- | --- |  | 3 | ------- | 0 |  |
|  | GLU | 46 | ------- | -0.62 | ------- | -0.62 | Conserv | 7 | ------- | 0.05 |  |
|  | VAL | 47 | ------- | -0.20 | ------- | -0.35 | Conserv | 7 | ------- | 0.58 |  |
|  | SER | 49 | ------- | -2.67 | ------- | -0.87 | ------- | 5 | ------- | 0 |  |
|  | LEU | 50 | Hotspot | 0.50 | ------- | -0.24 | ------- | 6 | ------- | 1.7 | Hotspot |
|  | PHE | 51 | ------- | --- | ------- | --- | Conserv | 9 | ------- | 0 |  |
|  | ASP | 54 | Hotspot | 0.37 | ------- | -0.49 | Conserv | 9 | Hotspot | 5.63 |  |
|  | LYS | 57 | ------- | -2.09 | ------- | -0.94 | Conserv | 7 | ------- | --- |  |
|  | LEU | 58 | ------- | -1.83 | ------- | -0.91 | Conserv | 7 | ------- | --- |  |
|  | ASN | 61 | ------- | -2.63 | ------- | -0.98 | ------- | 3 | ------- | --- |  |
|  | ASP | 73 | ------- | -2.76 | ------- | -0.94 | ------- | 4 | ------- | --- |  |
|  | PHE | 74 | ------- | -2.36 | ------- | -0.88 | ------- | 5 | ------- | 0.01 |  |
|  | SER | 75 | Hotspot | 0.55 | ------- | -0.64 | ------- | 5 | ------- | -0.25 |  |
|  | GLN | 76 | ------- | -1.60 | ------- | -0.87 | ------- | 3 | Hotspot | 2.37 |  |
|  | VAL | 77 | ------- | --- | ------- | --- | ------- | 6 | ------- | 0 |  |
|  | GLY | 78 | ------- | -1.00 | ------- | -0.60 | Conserv | 8 | ------- | --- |  |
|  | ALA | 79 | Hotspot | 1.31 | ------- | -0.33 | Conserv | 8 | ------- | --- | Hotspot |
|  | SER | 80 | ------- | -1.04 | ------- | -0.75 | Conserv | 7 | ------- | -0.01 |  |
|  | HIS | 82 | Hotspot | 1.48 | Hotspot | 0.10 | Conserv | 9 | ------- | 1.52 | Hotspot |
|  | GLN | 83 | Hotspot | 0.83 | ------- | -0.07 | Conserv | 9 | Hotspot | 3.09 | Hotspot |
|  | LYS | 85 | ------- | -2.39 | ------- | -0.92 | Conserv | 9 | ------- | --- |  |
|  | GLY | 86 | ------- | -0.61 | ------- | -0.70 | Conserv | 9 | ------- | --- |  |
|  | SER | 87 | ------- | -2.10 | ------- | -0.93 | Conserv | 8 | Hotspot | 2.63 |  |
|  | SER | 90 | ------- | -0.64 | ------- | -0.76 | Conserv | 8 | ------- | -0.03 |  |
|  | VAL | 91 | ------- | --- | ------- | --- | Conserv | 8 | ------- | 0.03 |  |
|  | LYS | 104 | ------- | -0.02 | ------- | -0.33 | ------- | 6 | ------- | 1.51 |  |
|  | CYS | 107 | ------- | -0.56 | ------- | -0.60 | ------- | 6 | ------- | -0.01 |  |
|  | GLU | 108 | ------- | -1.75 | ------- | -0.97 | ------- | 6 | ------- | 0.65 |  |
|  | LYS | 110 | ------- | -2.82 | ------- | -0.93 | ------- | 1 | ------- | --- |  |
|  | | | | | | | | | | | |
| Chain | Residue | Number | KFC2-A | | KFC2-B | | ConSurf | | Rosetta | | PPCheck |
|  |  |  | Class | Confidence | Class | Confidence | Class | Value | Class | DDG | Class |
| B | LEU | 32 | ------- | -1.13 | ------- | -0.42 | Conserv | 8 | ------- | 0.22 |  |
|  | LEU | 35 | ------- | -1.45 | ------- | -0.89 | Conserv | 7 | ------- | 0.25 |  |
|  | ASP | 38 | ------- | -2.33 | ------- | -1.01 | ------- | 6 | ------- | -0.14 |  |
|  | GLY | 39 | ------- | -0.77 | ------- | -0.70 | ------- | 3 | ------- | --- |  |
|  | SER | 40 | ------- | -0.73 | ------- | -0.71 | ------- | 4 | ------- | -0.08 |  |
|  | PRO | 41 | ------- | -3.48 | ------- | -0.81 | ------- | 2 | ------- | --- |  |
|  | ASP | 42 | ------- | -2.44 | ------- | -0.99 | ------- | 4 | ------- | 0.07 |  |
|  | PHE | 43 | Hotspot | 1.01 | Hotspot | 0.31 | ------- | 4 | ------- | 1.78 | Hotspot |
|  | SER | 45 | ------- | --- | ------- | --- |  | 3 | ------- | 0 |  |
|  | GLU | 46 | Hotspot | 0.19 | ------- | -0.29 | Conserv | 7 | Hotspot | 2.03 |  |
|  | VAL | 47 | Hotspot | 0.68 | ------- | -0.02 | Conserv | 7 | ------- | 0.81 |  |
|  | SER | 49 | ------- | -3.46 | ------- | -0.80 | ------- | 5 | ------- | --- |  |
|  | LEU | 50 | Hotspot | 0.73 | ------- | -0.13 | ------- | 6 | ------- | 1.51 |  |
|  | PHE | 51 | ------- | -0.02 | Hotspot | 0.07 | Conserv | 9 | ------- | 0.02 |  |
|  | ASP | 54 | ------- | -0.53 | ------- | -0.63 | Conserv | 9 | ------- | 1.96 |  |
|  | LYS | 57 | ------- | -2.45 | ------- | -0.97 | Conserv | 7 | ------- | --- |  |
|  | LEU | 58 | ------- | -1.84 | ------- | -0.83 | ------- | 6 | ------- | --- |  |
|  | ASP | 73 | ------- | -2.43 | ------- | -0.97 | ------- | 4 | ------- | --- |  |
|  | PHE | 74 | ------- | -2.39 | ------- | -0.89 | ------- | 5 | ------- | 0.01 |  |
|  | SER | 75 | Hotspot | 0.90 | ------- | -0.43 | ------- | 5 | ------- | -0.22 | Hotspot |
|  | GLN | 76 | ------- | -1.43 | ------- | -0.84 | ------- | 3 | ------- | 0.79 |  |
|  | VAL | 77 | ------- | --- | ------- | --- |  | 6 | ------- | 0 |  |
|  | GLY | 78 | ------- | -0.50 | ------- | -0.50 | Conserv | 8 | ------- | --- |  |
|  | ALA | 79 | Hotspot | 0.93 | ------- | -0.45 | Conserv | 8 | ------- | --- |  |
|  | SER | 80 | ------- | -1.67 | ------- | -0.86 | Conserv | 7 | ------- | 0.28 |  |
|  | HIS | 82 | Hotspot | 1.21 | Hotspot | 0.13 | Conserv | 9 | ------- | 1.11 | Hotspot |
|  | GLN | 83 | Hotspot | 0.14 | ------- | -0.23 | Conserv | 9 | Hotspot | 3.47 | Hotspot |
|  | LYS | 85 | ------- | -2.02 | ------- | -0.95 | Conserv | 9 | ------- | 0.05 |  |
|  | GLY | 86 | ------- | -0.79 | ------- | -0.69 | Conserv | 9 | ------- | --- |  |
|  | SER | 87 | ------- | -1.64 | ------- | -0.91 | Conserv | 8 | ------- | -0.03 |  |
|  | SER | 90 | ------- | -1.21 | ------- | -0.87 | Conserv | 8 | ------- | -0.05 |  |
|  | VAL | 91 | ------- | -2.39 | ------- | -1.01 | Conserv | 8 | ------- | --- |  |
|  | LYS | 104 | ------- | -0.14 | ------- | -0.28 | ------- | 6 | ------- | 1.03 |  |
|  | CYS | 107 | ------- | -0.40 | ------- | -0.45 | ------- | 6 | ------- | -0.02 |  |
|  | GLU | 108 | ------- | -1.78 | ------- | -0.96 | ------- | 6 | ------- | 0.13 |  |
|  | LYS | 110 | ------- | -2.27 | ------- | -0.91 | ------- | 1 | ------- | 0.72 |  |

| Chain A | HSDC | ASA | BSA | % of BSA | ΔiG |  | Chain B | HSDC | ASA | BSA | % of BSA | ΔiG |
| --- | --- | --- | --- | --- | --- | --- | --- | --- | --- | --- | --- | --- |
| A:GLU  31 | S | 84.53 | 17.14 | 20 | -0.28 |  | B:LEU  32 |  | 10.05 | 6.43 | 64 | 0.10 |
| A:LEU  32 |  | 8.69 | 8.36 | 96 | 0.13 |  | B:LEU  35 | H | 98.43 | 35.19 | 36 | 0.47 |
| A:LEU  35 | H | 70.30 | 32.86 | 47 | 0.50 |  | B:ASP  38 | HS | 90.85 | 17.70 | 19 | -0.05 |
| A:ASP  38 | HS | 70.70 | 39.24 | 56 | 0.23 |  | B:GLY  39 | H | 80.48 | 62.08 | 77 | 0.16 |
| A:GLY  39 | H | 92.49 | 75.59 | 82 | 0.26 |  | B:SER  40 |  | 65.63 | 48.19 | 73 | 0.29 |
| A:SER  40 |  | 53.53 | 32.50 | 61 | 0.31 |  | B:PRO  41 |  | 87.80 | 0.17 | 0 | 0.00 |
| A:ASP  42 |  | 23.66 | 8.88 | 38 | 0.14 |  | B:ASP  42 |  | 63.32 | 9.21 | 15 | 0.15 |
| A:PHE  43 |  | 72.84 | 70.18 | 96 | 1.11 |  | B:PHE  43 |  | 69.35 | 63.07 | 91 | 0.95 |
| A:GLU  46 |  | 83.41 | 48.48 | 58 | 0.70 |  | B:GLU  46 | H | 102.66 | 78.93 | 77 | -0.46 |
| A:VAL  47 |  | 34.86 | 26.10 | 75 | 0.41 |  | B:VAL  47 |  | 34.14 | 33.14 | 97 | 0.50 |
| A:SER  49 |  | 36.26 | 0.67 | 2 | 0.01 |  | B:SER  49 |  | 35.60 | 2.01 | 6 | 0.03 |
| A:LEU  50 |  | 117.83 | 94.03 | 80 | 1.50 |  | B:LEU  50 | H | 130.63 | 101.04 | 77 | 1.62 |
| A:ASP  54 | H | 34.11 | 27.18 | 80 | -0.34 |  | B:PHE  51 |  | 7.65 | 7.00 | 92 | 0.08 |
| A:LYS  57 |  | 86.86 | 24.46 | 28 | 0.37 |  | B:ASP  54 | H | 50.19 | 37.29 | 74 | -0.42 |
| A:LEU  58 |  | 23.91 | 7.82 | 33 | 0.13 |  | B:LYS  57 |  | 79.06 | 11.45 | 14 | -0.10 |
| A:ASN  61 |  | 73.73 | 5.83 | 8 | -0.07 |  | B:LEU  58 |  | 22.60 | 10.38 | 46 | 0.17 |
| A:ASP  73 |  | 56.98 | 2.85 | 5 | 0.05 |  | B:ASP  73 |  | 77.06 | 4.96 | 6 | -0.06 |
| A:PHE  74 |  | 44.12 | 3.87 | 9 | 0.05 |  | B:PHE  74 |  | 24.15 | 4.33 | 18 | 0.04 |
| A:SER  75 |  | 93.52 | 74.20 | 79 | 0.71 |  | B:SER  75 |  | 88.29 | 81.36 | 92 | 0.61 |
| A:GLN  76 | H | 112.60 | 45.92 | 41 | -0.00 |  | B:GLN  76 |  | 94.12 | 39.67 | 42 | -0.38 |
| A:GLY  78 |  | 23.12 | 13.37 | 58 | 0.15 |  | B:GLY  78 |  | 12.01 | 10.60 | 88 | 0.15 |
| A:ALA  79 |  | 56.65 | 56.41 | 100 | 0.83 |  | B:ALA  79 |  | 49.23 | 48.37 | 98 | 0.67 |
| A:SER  80 |  | 15.78 | 8.29 | 53 | -0.04 |  | B:SER  80 |  | 2.93 | 1.34 | 46 | 0.02 |
| A:HIS  82 |  | 128.73 | 115.22 | 90 | 0.27 |  | B:HIS  82 |  | 103.39 | 95.47 | 92 | 0.08 |
| A:GLN  83 | H | 121.08 | 96.58 | 80 | -0.80 |  | B:GLN  83 | H | 90.70 | 66.06 | 73 | -0.26 |
| A:LYS  85 |  | 65.15 | 5.95 | 9 | -0.03 |  | B:LYS  85 | S | 67.46 | 18.13 | 27 | -0.43 |
| A:GLY  86 |  | 32.92 | 22.25 | 68 | 0.36 |  | B:GLY  86 |  | 36.72 | 24.28 | 66 | 0.39 |
| A:SER  87 |  | 22.53 | 2.65 | 12 | 0.02 |  | B:SER  87 |  | 29.57 | 8.10 | 27 | -0.01 |
| A:SER  90 |  | 68.67 | 46.94 | 68 | 0.07 |  | B:SER  90 |  | 73.43 | 41.91 | 57 | 0.32 |
| A:LYS 104 | HS | 139.03 | 95.80 | 69 | -1.03 |  | B:VAL  91 |  | 8.54 | 1.84 | 22 | 0.03 |
| A:CYS 107 |  | 13.14 | 8.45 | 64 | 0.01 |  | B:LYS 104 | HS | 111.86 | 82.06 | 73 | -1.11 |
| A:GLU 108 | H | 123.39 | 40.24 | 33 | 0.18 |  | B:CYS 107 |  | 9.90 | 8.39 | 85 | 0.07 |
| A:LYS 110 |  | 125.96 | 14.09 | 11 | 0.10 |  | B:GLU 108 |  | 110.43 | 34.64 | 31 | 0.53 |
|  |  |  |  |  |  |  | B:LYS 110 | H | 126.45 | 34.61 | 27 | -0.56 |

**Table S16**. Involvement in Hydrogen bond, Salt bridge, Disulphide bond, or Covalent link (HSDC), ASA - Accessible Surface Area (Å²), BSA - Buried Surface Area (Å²), Buried area percentage (% of BSA) and ΔiG - Solvation energy effect (kcal/mol) of interface residues of AHP2 homodimer.

|  | ARR1rd-AHP2 | | ARR2rd-AHP2 | | ARR10rd-AHP2 | | ARR11rd-AHP2 | | StRR1a(rd)-StHP1a | | StRR11rd-StHP1a | |
| --- | --- | --- | --- | --- | --- | --- | --- | --- | --- | --- | --- | --- |
|  | mutated residue | ΔΔG of complex (kJ/mol) | mutated residue | ΔΔG of complex (kJ/mol) | mutated residue | ΔΔG of complex (kJ/mol) | mutated residue | ΔΔG of complex (kJ/mol) | mutated residue | ΔΔG of complex (kJ/mol) | mutated residue | ΔΔG of complex (kJ/mol) |
|  | D 43 | 1.25 |  |  | D 23 | 1.52 |  |  |  |  |  |  |
|  | D 44 | -0.15 | D 35 | -0.18 | D 24 | -0.16 | D 18 | -0.16 | D 36 | -0.16 |  |  |
|  | D 45 | 0.49 | D 36 | 3.80 | D 25 | 2.05 | D 19 | 3.8 | D 37 | 6.56 | D 31 | 3.48 |
|  |  |  |  |  | Q 26 | 0.10 |  |  |  |  |  |  |
|  | T 47 | 4.21 | T 38 | 1.49 | T 27 | 2.13 | T 21 | 1.27 | T 39 | 4.64 | T 33 | 1.79 |
|  | C 48 | -0.06 | C 39 | -0.10 | C 28 | -0.08 | W 22 | 1.12 | C 40 | -0.03 | W 34 | 1.46 |
|  |  |  | L 40 | 0.00 |  |  |  |  |  |  |  |  |
|  | M 50 | 0.97 | M 41 | 0.96 | R 30 | 3.07 | K 24 | 1.84 | K 42 | 1.04 | K 36 | 2.34 |
|  | I 51 | 1.78 | I 42 | 1.87 | I 31 | 1.70 | I 25 | 1.93 | I 43 | 2.00 | I 37 | 1.52 |
|  | E 53 | 0.00 | E 44 | -0.01 | Q 33 | 1.83 |  |  |  |  |  |  |
|  | R 54 | 1.60 | R 45 | 2.22 | T 34 | 1.33 | K 28 | 2.57 | K 46 | 2.81 | K 40 | 1.95 |
|  | M 55 | 0.62 | M 46 | 0.69 | L 35 | 0.87 | M 29 | 0.7 | M 47 | 0.88 | M 41 | 0.86 |
|  | R 57 | 1.61 | M 48 | 0.01 | Q 37 | 0.86 | K 31 | -0.01 | R 49 | 0.74 | K 43 | 0.07 |
|  | T 58 | 0.26 | T 49 | 0.63 |  |  | K 32 | 0.44 | N 50 | 0.06 | K 44 | 0.23 |
|  | C 59 | -0.03 | C 50 | -0.04 | C 39 | -0.09 |  |  |  |  |  |  |
|  |  |  |  |  | Q 40 | 0.34 |  |  |  |  |  |  |
|  | H 91 | 0.95 |  |  | D 71 | 0.60 | N 65 | 0.16 | H 83 | 0.86 |  |  |
|  |  |  |  |  |  |  | V 91 | 0.54 |  |  | V 103 | 0.49 |
|  |  |  |  |  | H 98 | -0.03 | D 92 | 0.33 | D 110 | -0.09 | D 104 | 0.19 |
|  | D 119 | 0.62 | D 110 | 0.30 | S 99 | 1.42 |  |  | D 111 | -0.26 |  |  |
|  | S 120 | -0.02 | S 111 | -0.03 | D 100 | 1.30 | E 94 | 0.42 | S 112 | 0.45 | E 106 | 0.00 |
|  | K 138 | 2.43 | K 129 | 1.87 | K 118 | 2.71 | K 112 | 4.84 | K 130 | 3.77 | K 124 | 1.32 |
|  | V 140 | -0.01 | V 131 | -0.01 | V 120 | -0.01 | I 114 | -0.01 | V 132 | 0.00 | I 126 | 0.08 |
|  | R 141 | -0.03 | R 132 | 0.86 | R 121 | 1.20 | R 115 | 0.69 | R 133 | 0.80 | R 127 | 2.37 |
|  | M 142 | -0.02 | I 133 | 0.85 | I 122 | 0.24 | M 116 | 0.36 | I 134 | 1.11 | M 128 | 0.52 |
|  |  |  |  |  |  |  | K 117 | -0.13 |  |  |  |  |
|  | | | | | | | | | | | | |
|  |  |  |  |  |  |  |  |  |  |  | D 24 | 0.61 |
|  |  |  | D 27 | 1.71 |  |  | D 27 | 0.37 |  |  |  |  |
|  | Q 28 | 1.83 | Q 28 | 1.93 | Q 28 | 1.76 | Q 28 | 1.87 | Q 26 | 1.86 | Q 26 | 3.03 |
|  | E 31 | 1.94 | E 31 | 0.54 | E 31 | 2.04 | E 31 | 1.18 | Q 29 | 1.40 | Q 29 | 1.64 |
|  | L 32 | 0.43 | L 32 | 0.6 | L 32 | 0.76 | L 32 | 0.61 | L 30 | 0.63 | L 30 | 0.39 |
|  | K 34 | 0.93 | K 34 | 0.1 | K 34 | 0.20 | K 34 | 0.15 | Q 32 | 0.05 | Q 32 | 0.01 |
|  | L 35 | 1.94 | L 35 | 2.34 | L 35 | 2.37 | L 35 | 2.47 | L 33 | 2.29 | L 33 | 2.11 |
|  | D 37 | 1.08 | D 37 | 0.23 | D 37 | -0.36 | D 37 | 0.27 |  |  | D 35 | 0.09 |
|  |  |  |  |  |  |  |  |  |  |  | S 37 | -0.03 |
|  | S 40 | 0.51 | S 40 | -0.09 | S 40 | -0.03 | S 40 | -0.09 | N 38 | 0.41 | N 38 | 0.00 |
|  | F 43 | 1.88 | F 43 | 1.74 | F 43 | 1.37 | F 43 | 1.85 | F 41 | 1.72 | F 41 | 1.84 |
|  | E 46 | 0.48 | E 46 | 1.24 | E 46 | 1.06 | E 46 | 1.80 | E 44 | -0.03 | E 44 | 0.26 |
|  | V 47 | 0.84 | V 47 | 0.73 | V 47 | 0.63 | V 47 | 0.84 | V 45 | 0.88 | V 45 | 0.87 |
|  | L 50 | 1.30 | L 50 | 1.48 | L 50 | 1.28 | L 50 | 1.27 | L 48 | 1.34 | L 48 | 1.46 |
|  | F 51 | 0.08 | F 51 | 0.08 | F 51 | 0.08 | F 51 | 0.16 | F 49 | 0.08 | F 49 | 0.08 |
|  | D 54 | 1.07 |  |  |  |  |  |  |  |  | D 52 | 1.11 |
|  | H 82 | 1.08 | H 82 | 1.7 | H 82 | 2.30 | H 82 | 2.18 | H 79 | 2.51 | H 79 | 0.68 |
|  | Q 83 | 2.68 | Q 83 | 1.39 | Q 83 | 0.51 | Q 83 | 3.10 | Q 80 | 1.58 | Q 80 | 1.77 |
|  | K 85 | 0.10 | K 85 | 0.59 | K 85 | 1.43 | K 85 | 0.73 | K 82 | 1.84 | K 82 | 0.99 |
|  | S 87 | 3.15 | S 87 | 2.69 | S 87 | 2.44 | S 87 | 4.07 | S 84 | 5.65 | S 84 | 2.41 |
|  | S 88 | 0.00 |  |  | S 88 | 0.00 |  |  |  |  |  |  |
|  | S 89 | -0.07 | S 89 | 0.64 | S 89 | 1.58 | S 89 | -0.05 | S 86 | -0.10 | S 86 | -0.04 |
|  | S 90 | 0.59 | S 90 | 0.39 | S 90 | -0.36 | S 90 | -0.17 | S 87 | 2.62 | S 87 | -0.17 |
|  | V 91 | 0.15 | V 91 | 0.2 | V 91 | 0.09 | V 91 | 0.05 | V 88 | 0.05 | V 88 | 0.19 |
|  | K 97 | 0.68 |  |  |  |  |  |  | K 94 | -0.10 | K 94 | 0.18 |
|  | V 101 | 0.02 |  |  |  |  |  |  | V 98 | 0.21 | V 98 | 0.07 |
|  |  |  | K 104 | 1.21 | K 104 | 2.01 |  |  | R 101 | 1.27 |  |  |
|  |  |  |  |  |  |  | I 155 | 0.06 |  |  |  |  |
|  |  |  |  |  |  |  | N 156 | 0.06 |  |  |  |  |

**Table S17**. Virtual alanine scanning for modeled RR(rd) –HPt complexes. Hot spots with ΔΔG > 4 kJ/mol colored yellow, hot spots with ΔΔG between 2 and 4 kJ/mol colored red.

**Table S18**. Involvement in Hydrogen bond, Salt bridge, Disulphide bond, or Covalent link (HSDC), ASA - Accessible Surface Area (Å²), BSA - Buried Surface Area (Å²), Buried area percentage (% of BSA) and ΔiG - Solvation energy effect (kcal/mol) of interface residues of ARR1rd–AHP2.

| Chain A - ARR1rd | HSDC | ASA | BSA | % of BSA | ΔiG |  | Chain B - AHP2 | HSDC | ASA | BSA | % of BSA | ΔiG |
| --- | --- | --- | --- | --- | --- | --- | --- | --- | --- | --- | --- | --- |
| A:ASP  44 | H | 60.35 | 26.87 | 45 | -0.09 |  | B:ASP  26 |  | 39.55 | 0.37 | 1 | -0.00 |
| A:ASP  45 |  | 51.36 | 48.01 | 93 | 0.31 |  | B:ASP  27 |  | 115.88 | 9.27 | 8 | 0.00 |
| A:PRO  46 |  | 98.93 | 51.11 | 52 | 0.76 |  | B:GLN  28 | H | 72.54 | 59.51 | 82 | -0.53 |
| A:THR  47 |  | 85.76 | 85.76 | 100 | 0.76 |  | B:GLU  31 | H | 69.97 | 58.64 | 84 | -0.32 |
| A:CYS  48 | H | 28.64 | 25.96 | 91 | 1.03 |  | B:LEU  32 |  | 10.55 | 10.04 | 95 | 0.16 |
| A:MET  50 |  | 111.25 | 73.49 | 66 | 1.92 |  | B:LYS  34 |  | 86.67 | 32.17 | 37 | 0.22 |
| A:ILE  51 |  | 74.42 | 73.24 | 98 | 1.17 |  | B:LEU  35 |  | 103.33 | 100.81 | 98 | 1.34 |
| A:ARG  54 | HS | 141.54 | 90.08 | 64 | 0.38 |  | B:ASP  37 | HS | 79.56 | 37.55 | 47 | -0.18 |
| A:MET  55 |  | 43.00 | 43.00 | 100 | 0.68 |  | B:GLY  39 | H | 70.51 | 20.92 | 30 | -0.01 |
| A:ARG  57 | HS | 142.41 | 59.26 | 42 | -1.50 |  | B:SER  40 | H | 37.57 | 28.59 | 76 | 0.13 |
| A:THR  58 |  | 96.01 | 40.87 | 43 | 0.53 |  | B:PHE  43 |  | 43.58 | 43.58 | 100 | 0.70 |
| A:CYS  59 |  | 35.47 | 1.68 | 5 | 0.03 |  | B:GLU  46 | HS | 95.10 | 38.42 | 40 | -0.12 |
| A:HIS  91 |  | 118.46 | 33.66 | 28 | 0.06 |  | B:VAL  47 |  | 32.73 | 32.56 | 99 | 0.50 |
| A:ALA 117 |  | 43.98 | 30.56 | 69 | 0.49 |  | B:LEU  50 |  | 128.44 | 74.71 | 58 | 1.20 |
| A:ASP 118 | H | 75.95 | 4.57 | 6 | -0.05 |  | B:PHE  51 |  | 4.06 | 4.06 | 100 | 0.05 |
| A:ASP 119 | HS | 134.75 | 77.79 | 58 | -0.27 |  | B:ASP  54 |  | 25.94 | 16.33 | 63 | -0.06 |
| A:SER 120 |  | 72.93 | 24.25 | 33 | -0.22 |  | B:HIS  82 |  | 118.82 | 77.86 | 66 | -0.15 |
| A:LYS 138 |  | 30.31 | 11.29 | 37 | 0.11 |  | B:GLN  83 | H | 91.93 | 60.01 | 65 | -0.65 |
| A:PRO 139 |  | 122.66 | 71.56 | 58 | 0.93 |  | B:LYS  85 | HS | 53.25 | 48.94 | 92 | -1.10 |
| A:VAL 140 | H | 13.49 | 5.68 | 42 | -0.06 |  | B:GLY  86 |  | 39.03 | 27.38 | 70 | 0.43 |
| A:ARG 141 |  | 169.36 | 37.58 | 22 | 0.34 |  | B:SER  87 |  | 29.36 | 29.36 | 100 | 0.18 |
| A:MET 142 | H | 98.35 | 56.02 | 57 | 1.64 |  | B:SER  89 |  | 46.17 | 35.66 | 77 | 0.35 |
| A:GLU 143 |  | 72.01 | 1.32 | 2 | 0.02 |  | B:SER  90 | H | 71.01 | 70.15 | 99 | 0.14 |
|  |  |  |  |  |  |  | B:VAL  91 |  | 6.36 | 6.03 | 95 | 0.10 |
|  |  |  |  |  |  |  | B:LYS  97 | HS | 65.43 | 15.83 | 24 | -0.54 |
|  |  |  |  |  |  |  | B:VAL 101 |  | 72.91 | 20.25 | 28 | 0.32 |
|  |  |  |  |  |  |  | B:LYS 104 |  | 53.13 | 5.53 | 10 | 0.09 |

**Supplementary figures**


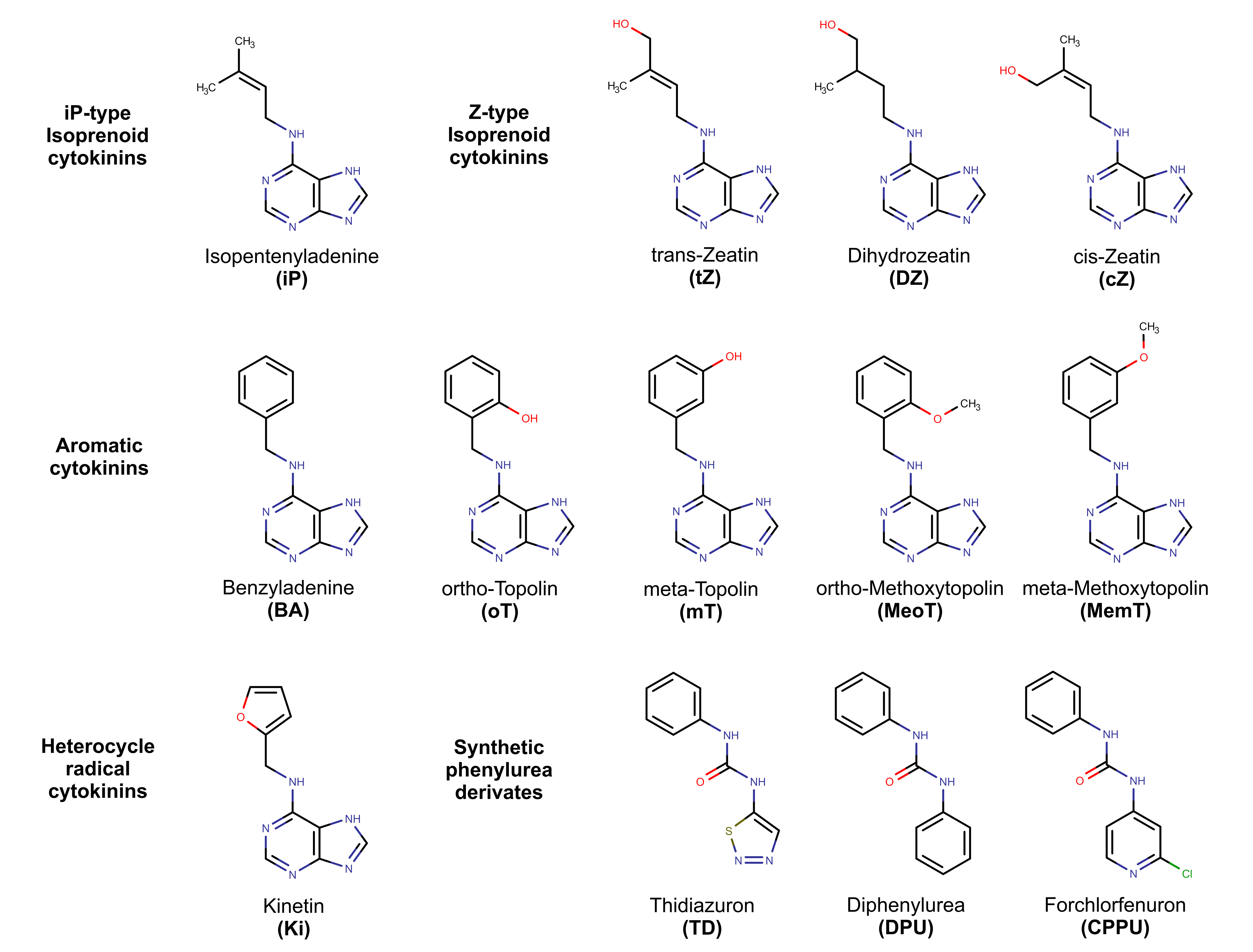


**Figure S1.** Structures of natural and synthetic cytokinins.


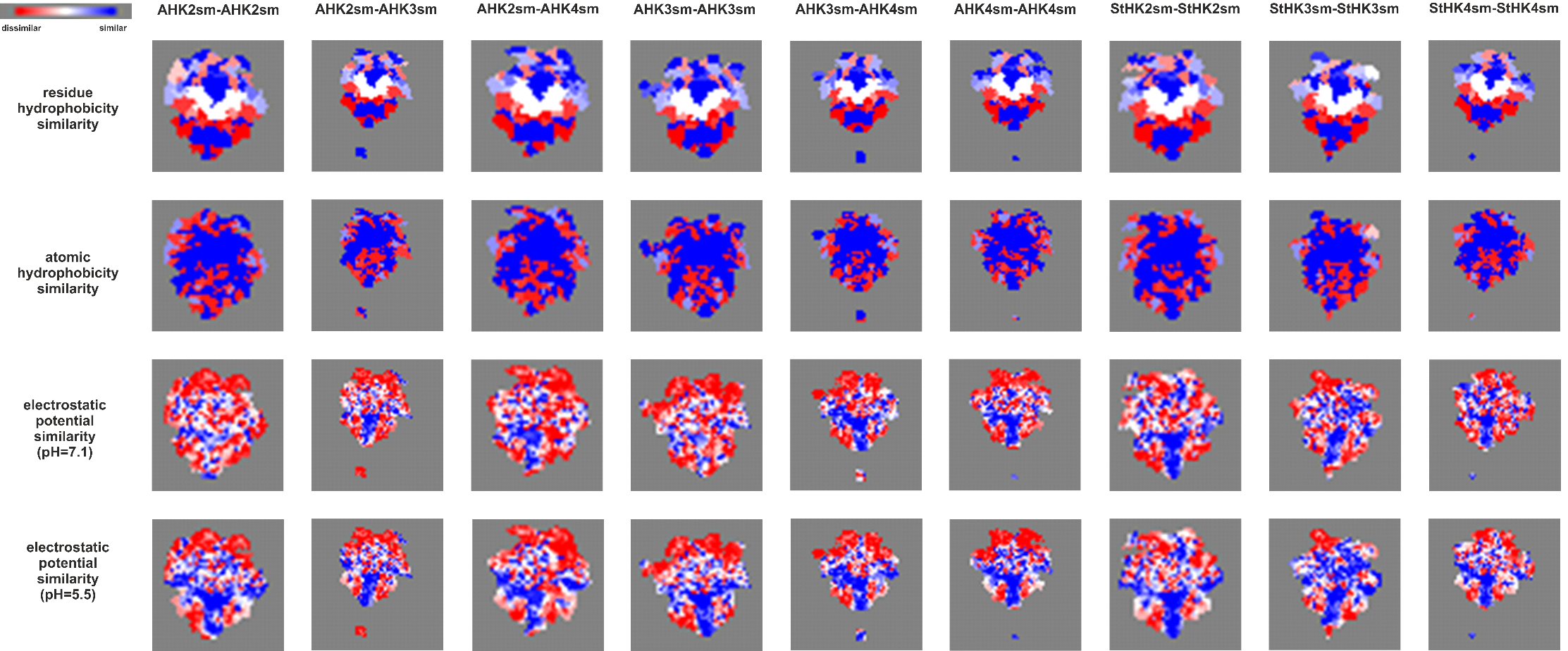


**Figure S2.** Maps of hydrophobic and electrostatic potential similarity of sensor module dimer interfaces, calculated with Molsurfer. Blue – most similar zones, red – most dissimilar zones. Similarity means complementarity for hydrophobicity. Dissimilarity means complementarity for electrostatic potential.


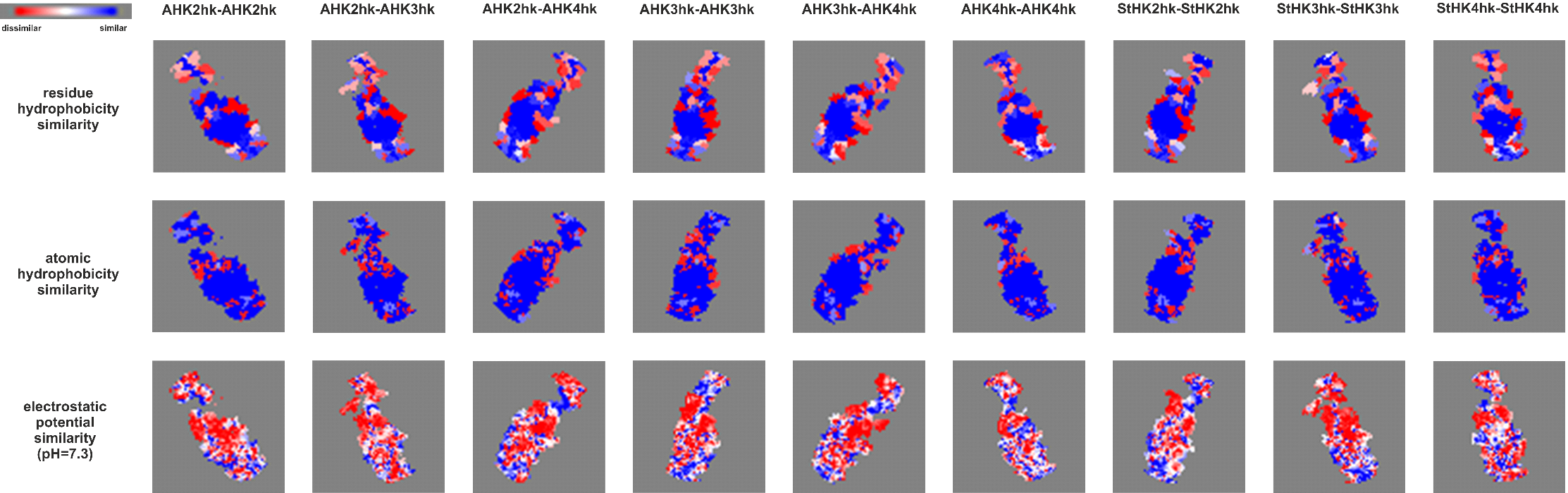


**Figure S3.** Maps of hydrophobic and electrostatic potential similarity of HisKA domains dimer interfaces, calculated with Molsurfer. Blue – most similar zones, red – most dissimilar zones. Similarity means complementarity for hydrophobicity. Dissimilarity means complementarity for electrostatic potential.


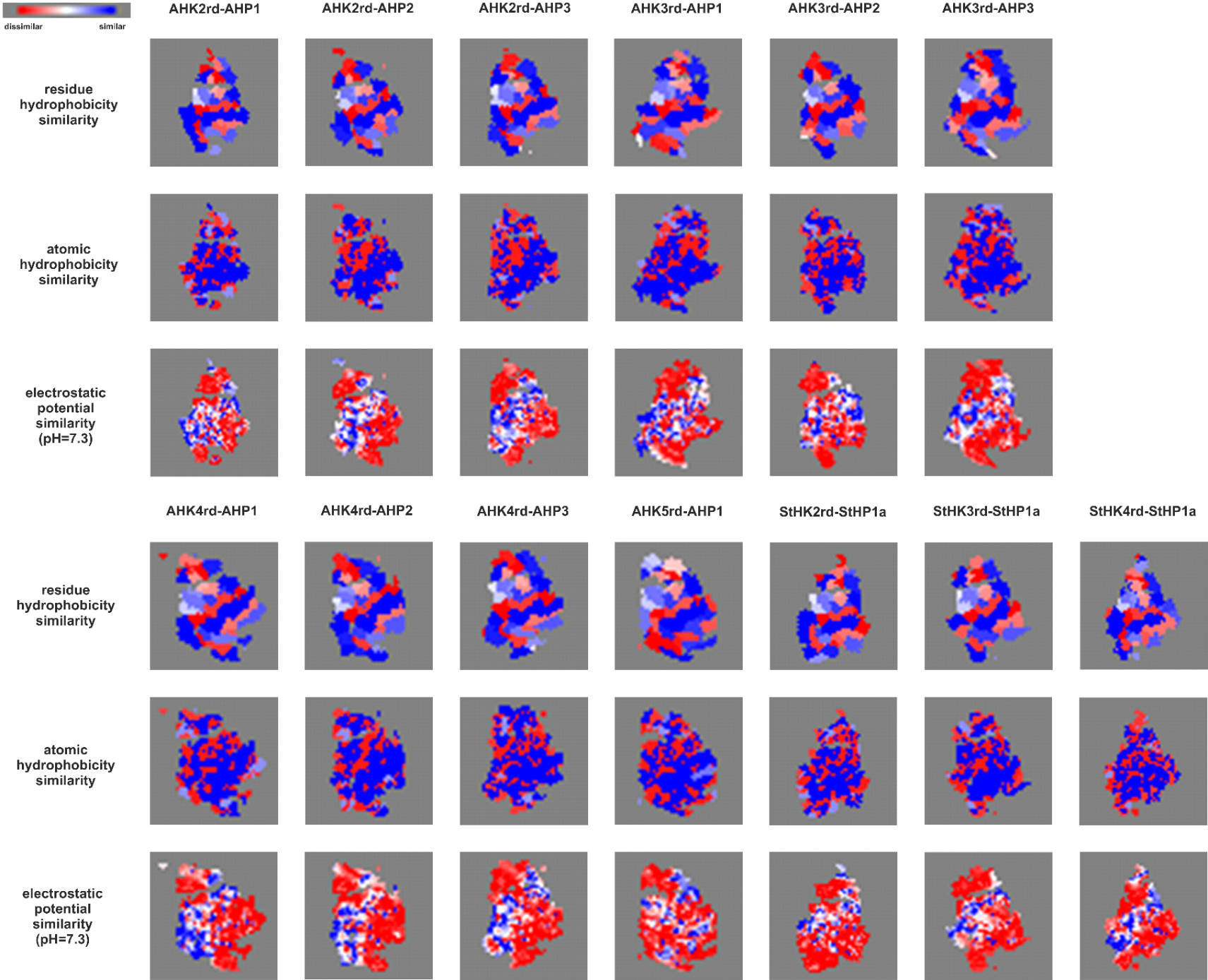


**Figure S4.** Maps of hydrophobic and electrostatic potential similarity of HKrd-HPt complex interfaces, calculated with Molsurfer. Blue – most similar zones, red – most dissimilar zones. Similarity means complementarity for hydrophobicity. Dissimilarity means complementarity for electrostatic potential.


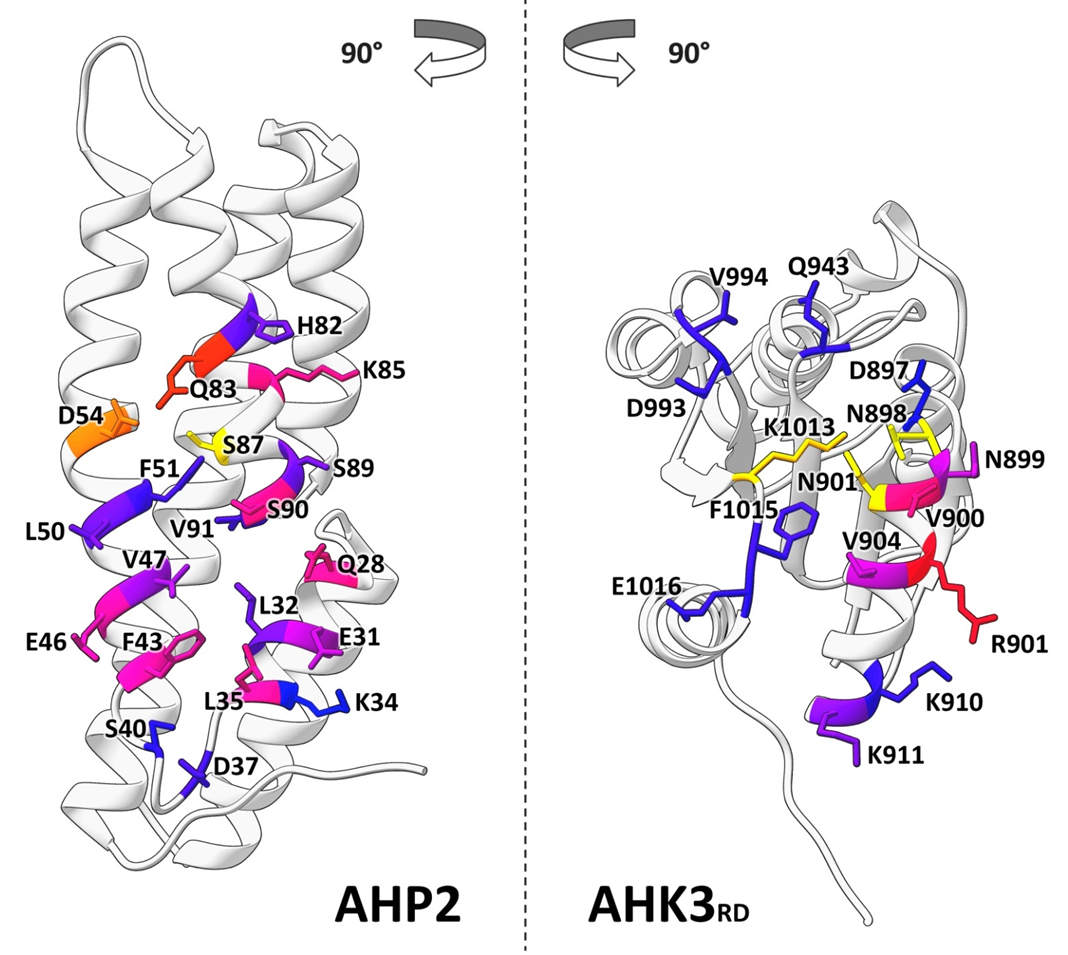


**Figure S5.** Alanine scanning results for AHK3rd-AHP2 complex model. Amino acid residues are colored by relative ΔΔG(complex) values - yellow is the highest ΔΔG, red – middle value, blue is the lowest ΔΔG. Color range is specific for each chain.


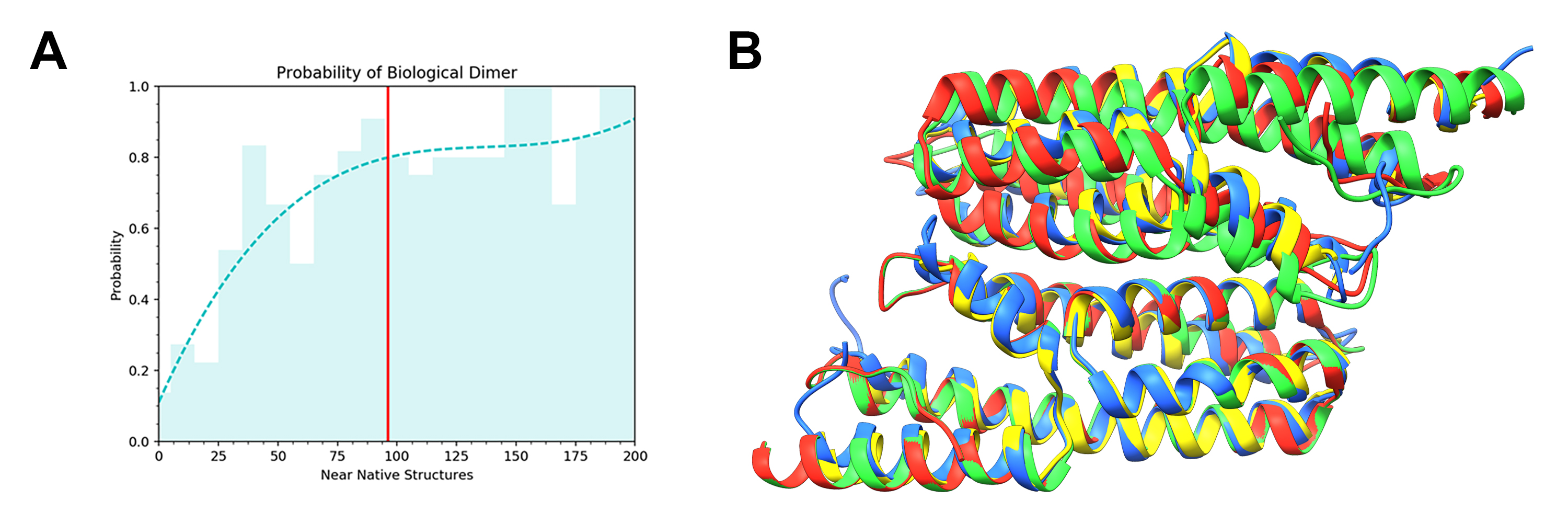


**Figure S6.** Biological relevance of OsHP1 (PDB ID: 1YVI) crystal structure dimer conformation. A - Probability of being a biological dimer for OsHP1 structure according to the Dimer Classification results in ClusPro. B - Comparison of the structures: yellow - OsHP1 crystal structure, blue - homology model of AHP2 dimer, red – the best ClusPro AHP2-AHP2 docking solution, green – the best PatchDock AHP2-AHP2 docking solution.


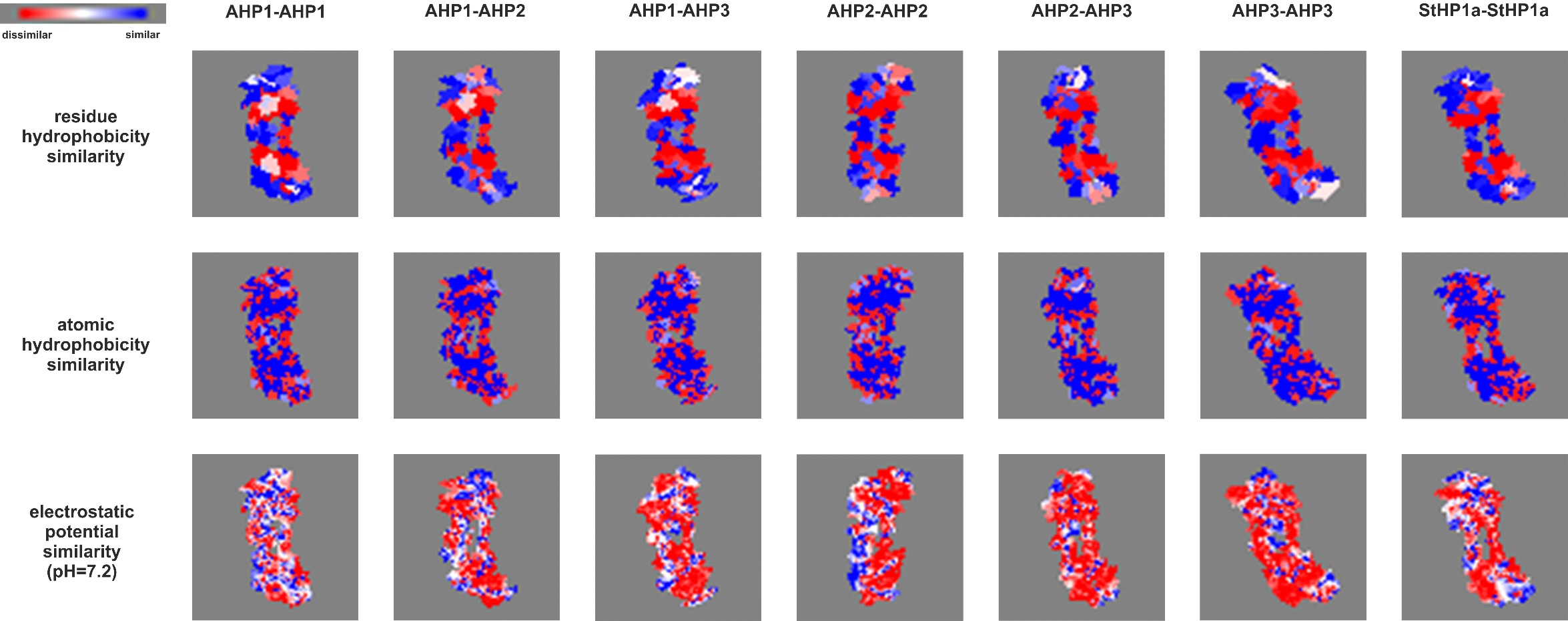


**Figure S7.** Maps of hydrophobic and electrostatic potential similarity for HPt-HPt dimer interfaces, calculated with Molsurfer. Blue – most similar zones, red – most dissimilar zones. Similarity means complementarity for hydrophobicity. Dissimilarity means complementarity for electrostatic potential.


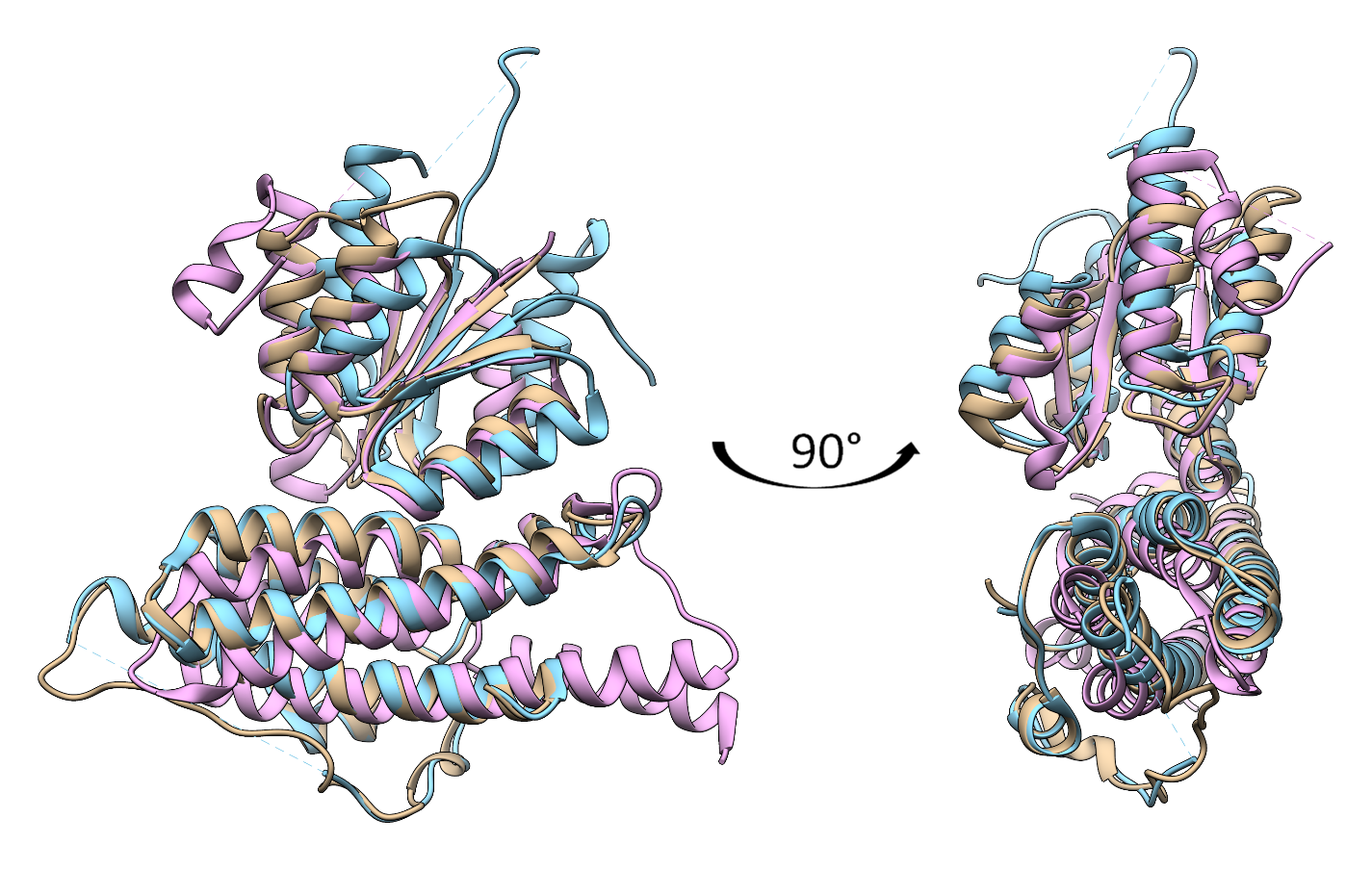


**Figure S8.** Alignment of X-ray structures: SLN1-YPD1 (PDB ID: 1OXB) – hazel, SSK1-YPD1 (PDB ID: 5KBX) – cyan, and AHK5(CKI2)-AHP1 (PDB ID: 4EUK) – magenta.


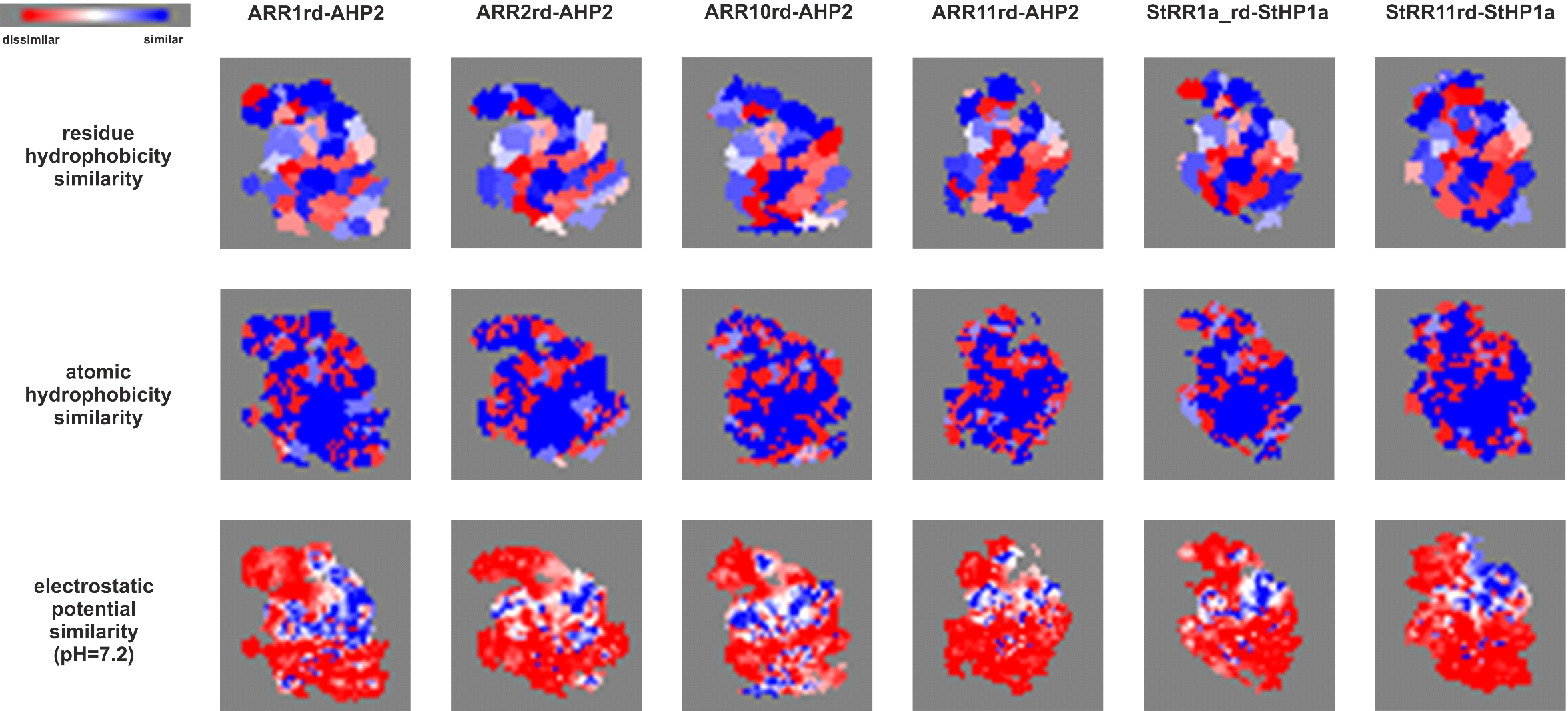


**Figure S9.** Maps of hydrophobic and electrostatic potential similarity for RRrd-HPt complex interfaces, calculated with Molsurfer. Blue – most similar zones, red – most dissimilar zones. Similarity means complementarity for hydrophobicity. Dissimilarity means complementarity for electrostatic potential.


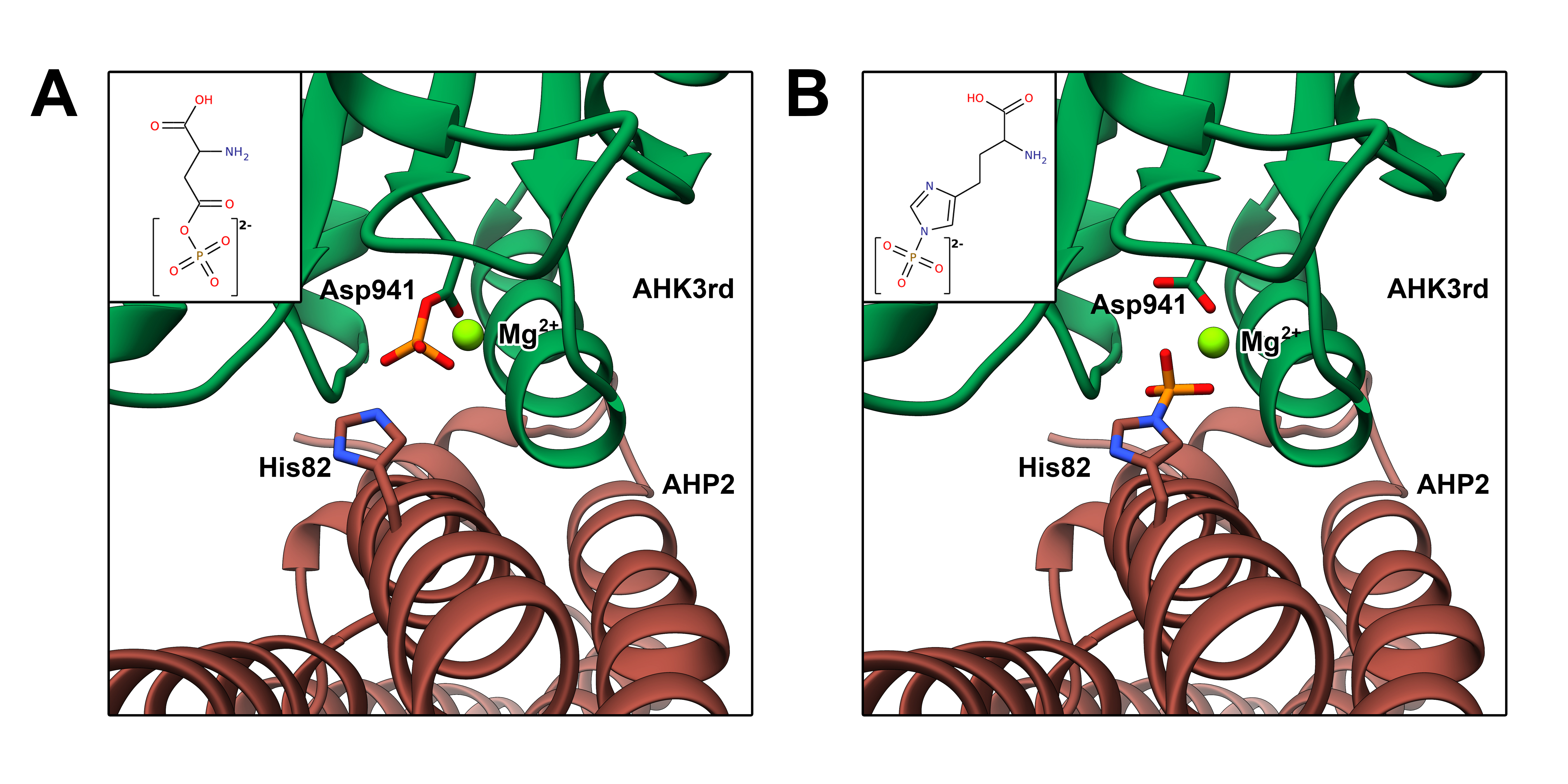


**Figure S10.** Models of AHK3rd-AHP2 complex in different phosphorylation states: A – phosphoaspartate in AHK3rd; B – phosphohistidine in AHP2.


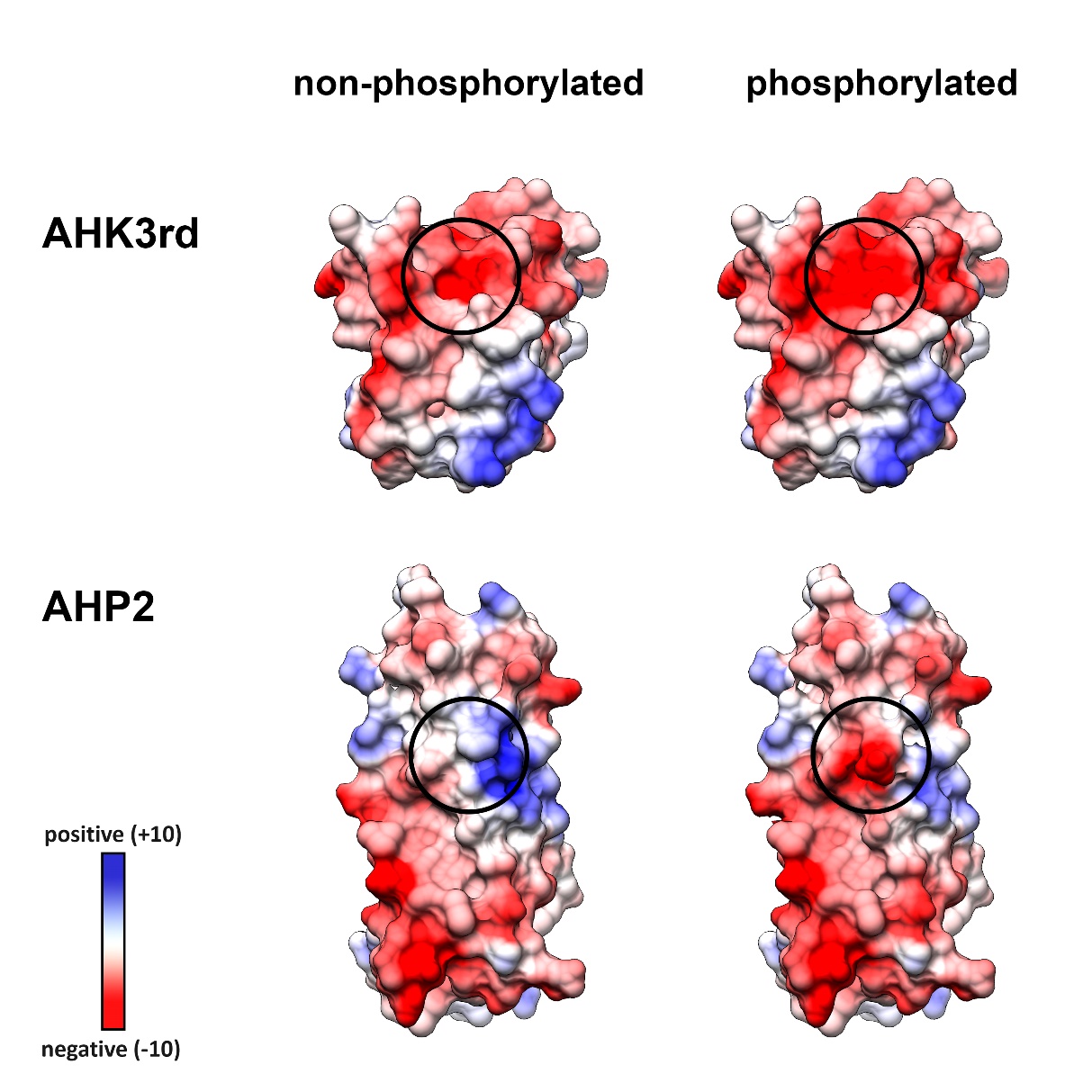


**Figure S11.** effect of phosphorylation on electrostatic potential of AHK3rd and AHP2 surfaces. Blue – positive electrostatic potential (+10), white – neutral (0), red – negative (-10).
